# Six New Diterpene Glycosides from the Soft Coral *Lemnalia* *bournei*

**DOI:** 10.3390/md19060339

**Published:** 2021-06-14

**Authors:** Xia Yan, Han Ouyang, Te Li, Yutong Shi, Bin Wu, Xiaojun Yan, Shan He

**Affiliations:** 1 Li Dak Sum Yip Yio Chin Kenneth Li Marine Biopharmaceutical Research Center, Department of Marine Pharmacy, College of Food and Pharmaceutical Sciences, Ningbo University, Ningbo 315800, China; yanxia@nbu.edu.cn (X.Y.); telinbu@163.com (T.L.); shiyutong@nbu.edu.cn (Y.S.); yanxiaojun@nbu.edu.cn (X.Y.); 2 Institute of Drug Discovery Technology, Ningbo University, Ningbo 315211, China; 3 Ocean College, Zhejiang University, Hangzhou 310058, China; wubin@zju.edu.cn

**Keywords:** soft coral, diterpene glycosides, *Lemnalia bournei*, antimicrobial activity

## Abstract

A chemical study on the extracts of soft coral *Lemnalia bournei* resulted in the isolation and identification of six new bicyclic diterpene glycosides including three new lemnaboursides E–G (**1**–**3**), and three new lemnadiolboursides A–C (**4**–**6**), along with three known lemnaboursides (**7**–**9**). Their structures were elucidated by detailed spectroscopic analysis, ECD analysis, chemical methods, and comparison with the literature data. Lemnadiolboursides A–C (**4**–**6**) contained a lemnal-1(10)-ene-7,12-diol moiety compared with the lemnaboursides. All these compounds were evaluated for antibacterial activity; cell growth inhibition of A549, Hela, HepG2, and CCRF-CEM cancer cell lines; and inhibition of LPS-induced NO production in RAW264.7 macrophages. The results indicated that compounds **1**, **2**, and **4**–**6** exhibited antibacterial activity against *Staphylococcus aureus* and *Bacillus subtilis* (MIC 4–16 μg/mL); compounds **1**–**9** displayed low cytotoxicity on the CCRF-CEM cell lines (IC_50_ 10.44–27.40 µM); and compounds **1**, **2,** and **5** showed weak inhibition against LPS-induced NO production (IC_50_ 21.56–28.06 μM).

## 1. Introduction

In recent years, marine invertebrates have afforded numerous structurally diverse and biologically active secondary metabolites. Soft corals of the genus *Lemnalia* (Coelenterata, Octocorallia, Alcyonacea, and Nephtheidae) consist of more than 30 species, which are widely distributed in the South China Sea, Taiwan, and off the coast of Australia and Kenya [1]. About 15 species of *Lemnalia* have been chemically investigated including *Lemnalia africana*, *Lemnalia flava*, *Lemnalia philippinensis*, *Lemnalia cervicornis*, *Lemnalia bournei*, *Lemnalia tenuis*, *Lemnalia laevis*, *Lemnalia humesi*, *Lemnalia carnosa*, and other unidentified *Lemnalia* sp. [1]. More than 120 terpenoids with diverse chemical structures have been isolated and identified from the extracts of *Lemnalia* sp., which exhibit broad biological properties including cytotoxic, antiviral, antimicrobial, and anti-inflammatory activities [1,2,3,4,5,6].

The diterpene glycosides from genus *Lemnalia* are all biflorane-type glycosides such as lemnaboursides, lemnaflavosides, lemnalosides, and their acetate derivatives [7,8,9,10]. Previous chemical investigations of *Lemnalia bournei* only provided four diterpene glycosides including lemnabourside and lemnaboursides B–D [9,10,11]. Lemnabourside was characterized with a D-glucose attached to a diterpene aldehyde through an acetal linkage, and lemnaboursides B and C were two analogs of lemnaboursides with monoacetylation of the sugar residue at different hydroxyl groups [1]. Biologically, these three metabolites only showed weak cytotoxicity.

**Figure 1:**
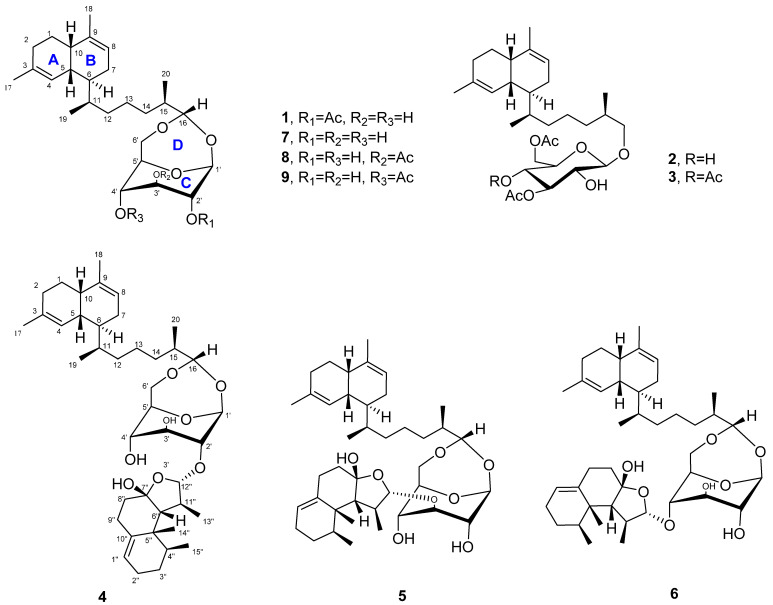
Structures of compounds **1**–**9**.

To explore the bioactive secondary metabolites from marine organisms, soft coral *L. bournei* were collected from the coast of Xisha Island (7 m deep) by SCUBA diving. The acetone extracts were chemically investigated, resulting in the discovery of six new bicyclic diterpene glycosides (**1**–**6**) and three known lemnaboursides (**7**–**9**) (Figure 1). Lemnabourside E (**1**) was 2′-O-acetate of lemnabourside (**7**), and lemnabourside F (**2**) and G (**3**) were ring D (Figure 1) opened derivatives of lemnabourside acetates. Moreover, lemnadiolboursides A−C (**4**−**6**) contained lemnal-1(10)-ene-7,12-diol moiety compared with lemnabourside (**7**). In this paper, we report the isolation, structure determination, and bioactivities of these compounds.

## 2. Results and Discussion

**Figure 2:**
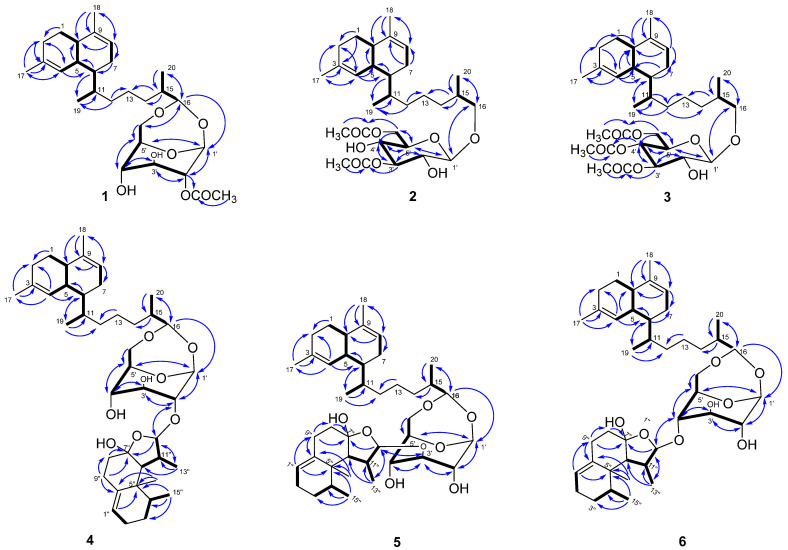
Key COSY (bold) and HMBC (blue) correlations of compounds **1**–**6**.

Lemnabourside E (**1**) was isolated as an amorphous solid. Its molecular formula was determined to be C_28_H_44_O_7_ by HRESIMS (*m/z* 515.2977, calcd for [M + Na]^+^ 515.2980), requiring seven degrees of unsaturation. The ^1^H NMR and HSQC spectra disclosed two trisubstituted olefinic bonds (*δ*_C_/*δ*_H_ 134.6, 124.0/5.48 and *δ*_C_/*δ*_H_ 136.7, 121.6/5.40), one sugar unit (*δ*_C_/*δ*_H_ 98.2/4.93, 79.7/4.56, 74.5/3.75, 66.2/4.32, 79.6/3.88, 67.9/3.96/3.55), one acetyl (*δ*_C_/*δ*_H_ 172.2, 21.1/2.16), another four methyls, six methylenes, and six methines. The spectral data of **1** were similar to those of diterpene glycosides isolated from the soft coral *Lemnalia bournei* (Table 1 and Table 2) with the differences reflecting the mono-acetylate position of the sugar residue [9]. Analysis of the COSY spectrum of **1** rapidly identified the sugar unit connection. In the HMBC experiment, the correlations from H-17 to C-3, and H-4 to C-17 suggested that C-3 is connected to C-17. The HMBC correlation between H-18 and C-10 indicated C-18 was located at C-9. In addition, the HMBC experiment showed correlations between H-10 and C-9, H-17 and C-2, H-20 and C-15, H-15 and C-1′, H-16 and C-6′, which indicated that C-9 was bonded to C-10, C-2 to C-3, C-20 to C-15, C-15 to C-16, and the presence of two acetal bonds. This evidence proved that **1** was a lemnabourside derivative, and the HMBC correlations from H-2′ to acetyl carbon *δ*_C_ 172.2 positioned the acetate group at C-2′ (Figure 2).

The configuration of the sugar unit was assigned after the hydrolysis of **1** with H_2_SO_4_. The hydrolysate reaction mixture was partitioned between CH_2_Cl_2_ and H_2_O. The CH_2_Cl_2_ yielded the decalin-type bicyclic diterpene aldehyde (see ^1^HNMR in Figure S56) according to the literature data [9]. The aqueous part was conducted with l-cysteine methyl ester and o-tolyl isothiocyanate and yielded methyl 2-(polyhydroxyalkyl)-3-(o-tolylthiocarbamoyl)-thiazolidine-4(*R*)-carboxylates, according to the reported method [12]. The HPLC retention time (Rt = 7.8 min) of the sugar derivative was compared with the standard sample prepared in the same manner (Figure S57). In this way, the sugar unit was determined to be D-glucose.

A *cis*-decalin configuration in the diterpene portion was deduced from the ^13^C chemical shifts in C-5 (*δ*_C_ 36.6) and C-10 (*δ*_C_ 39.7) as well as the small coupling constant (∼2 Hz) between H-1 and H-6, which were very similar to those of the separated known lemnaboursides (**7**–**9**). Moreover, H_3_-19/H-5 showed NOE interactions, but there were no NOE correlations between H_3_-19 and H-6, which suggested H_3_-19 and H-5 were positioned on the same orientation. The absolute configurations of the decalin part could also be deduced from the DFT/ECD calculations. The experimental ECD spectrum exhibited a negative Cotton effect (CE) at 202 nm, which was in good agreement with the calculated ECD spectra of (5*R*,6*S*,10*S*,11*S*)-**1** (Figure S1). The β-configuration of the glucose was confirmed by the 1D-NOE method as the reported method, and it was in the boat form [10]. The torsion angle of H-1′ and H-2′ was close to 90°, and the magnitude of the coupling was generally the smallest (close to 0). Moreover, H_3_-20/H-16 and H_3_-20/H-1′ also showed NOE interactions, which positioned H_3_-20, H-16, and H-1′ on the same face.

Lemnabourside F (**2**) was also a white solid with the chemical formula of C_30_H_48_O_8_ as revealed by the HRESIMS ion peak, indicating seven degrees of unsaturation. Acid hydrolysis of **2** also yielded D-glucose. The ^1^H NMR spectra of **2** exhibited most of the structural features found in **1**, with the major difference of ring D opened, and two acetyl groups (*δ*_C_/*δ*_H_ 172.3, 21.1/2.17 and *δ*_C_/*δ*_H_ 171.6, 20.9/2.11) and oxygenated methylene (*δ*_C_/*δ*_H_ 75.7/3.67/3.38) rather than oxygenated methine were present. The HMBC correlation of H-3′/3′-OAc and H-6′/6′-OAc suggested that the C-3′ and C-6′ hydroxyl groups of the glucose were acetylated (Figure 2). The ^1^H-^1^H COSY, HSQC, and HMBC experiments allowed for the complete assignment for structure **2**. Acid hydrolysis of **2** was discriminated in the same manner as **1.** The CH_2_Cl_2_ part yielded the decalin-type bicyclic diterpene alcohol (see ^1^HNMR in Figure S56). The absolute configurations of **2** were also proposed to be the same as that of **1** based on their identical ^13^C chemical shifts of C-5, C-6, C-10, and C-11 as well as on biosynthetic considerations. The coupling constant of the anomeric proton was about 7 Hz, which suggested that ring C was in the chair form, and the torsion angle of H-1′ and H-2′ was close to 180°.

Compound **3**, a white solid, had the molecular formula C_32_H_50_O_9_ as determined by HRESIMS. The ^1^H NMR spectra of **3** showed a high similarity to those of **2** except for the presence of three acetyl groups instead of two. These three acetyl groups were located at 3′-OH, 4′-OH, and 6′-OH, as confirmed by the HMBC correlation from H-3′ to 3′-OAc (*δ*_C_ 170.7), from H-4′ to 4′-OAc (*δ*_C_ 169.7), and from H-6′ to 6′-OAc (*δ*_C_ 170.7). The acid hydrolysis of **3** also yields D-glucose. ^1^H-^1^H COSY, HSQC, HMBC, and 1D-NOE experiments allowed for the complete assignment of the structure of **3**. Compound **3** was given the name lemnabourside G.

Compound **4**, a white solid named lemnadiolbourside A, had the molecular formula C_41_H_64_O_8_, established by HRESIMS and NMR data. The ^13^C and DEPT spectra exhibited a total of 41 carbon resonances (Table 2). Overall, the comparison of ^1^H and ^13^C NMR data of **4** and lemnabourside (**7**) revealed that **4** contained the structural unit of lemnabourside with 26 carbons [9]. The remaining 15 carbons belonged to a nardosinane-type sesquiterpenoid [13]. The analysis of the COSY spectrum of compound **4** revealed the presence of the lemnabourside unit and the nardosinane unit (Figure 2): (a) H-4/H-5/H-10/H_2_-1/H_2_-2, H-5/H-6/H_2_-7/H-8, H-6/H-11/H_2_-12/H_2_-13/H_2_-14/H-15/H-16, H-15/H_3_-20, H-11/H_3_-19, H-1′/H-2′/H-3′/H-4′/H-5′/H_2_-6′ and (b) H_2_-2″/H_2_-3″/H-4″/H_3_-15″, H_2_-8″/H_2_-9″, H-6″/H-11″/H-12″; H-11″/H_3_-13″. The HMBC correlations from H-2′ to C-12″ and H-12″ to C-2′ allowed us to identify the nardosinane located at 2′-OH of the lemnabourside unit (Figure 2). The acid hydrolysis of **4** was conducted following the above method, which yielded D-glucose and diterpene aldehyde, but failed to reveal the nardosinane moiety due to the inherent instability of lemnal-1(10)-ene-7,12-diol under strong acid and heating conditions.

**Figure 3:**
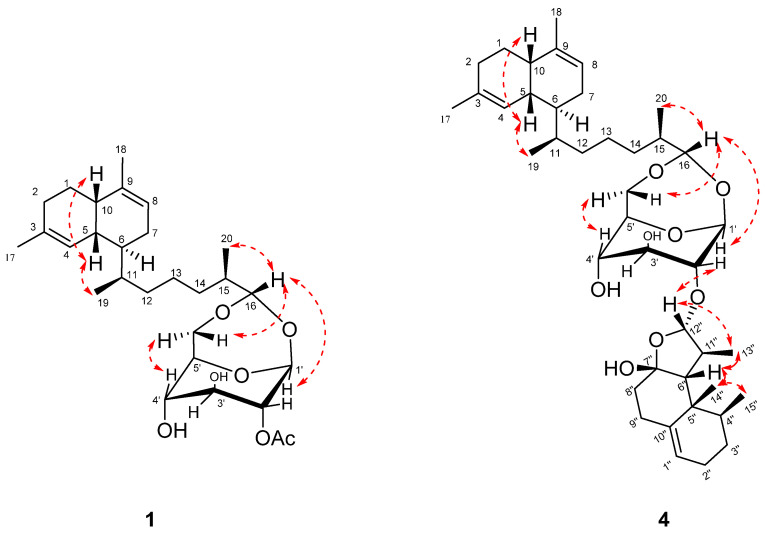
Key NOE correlations (red) of compounds **1** and **4**.

The relative configuration of **4** was deduced based onNOE correlations (Figure 3). For the nardosinane unit, the NOE correlations of H_3_-15″/H_3_-14″, H_3_-15″/H_3_-13″, H_3_-15″/H-6″, H-12″/H_3_-13″, and H-12″/H-2′ suggested H-2′, H-6″, H-12″, H_3_-13″, H_3_-14″, and H_3_-15″ were assigned as the *β*-configuration. The chiral center at C-7″, as it is a hemiketal linkage, and no NOE could be detected. The ^13^C chemical shifts in C-6″ (δ_C_ 59.1) and C-8″ (δ_C_ 36.3), which were very similar to the chemical shifts C-6 and C-8 of the reported compound lemnal-1(10)-ene-7,12-diol (58.2 and 35.8, respectively) [13], revealed that the 7″S* relative configuration corresponded to 6″R*. The absolute configurations of the nardosinane unit were also the same as lemnal-1(10)-ene-7,12-diol for biogenetic consideration [13]. The *cis*-configuration for H-5 and H-10 in the diterpene portion was deduced as **1**. The NOE correlations of H_3_-19/H-5, H-16/H_3_-20, and H-16/H-1′ were also observed. Given the same structural features of the lemnabourside, the absolute configurations of the lemnabourside unit in **4** werededuced to be lemnabourside (**7**), also on a biogenetic consideration.

The HRESIMS spectra and NMR data of compounds **5** and **6** were very similar to **4** (Table 1 and Table 2), thus, suggesting they were an isomer of **4**. The acid hydrolysis of **5** and **6** was conducted similar to **4** and the same hydrolysates were obtained. The major difference was that C-3′ (*δ*_C_ 86.9) of the glucose in **5** was shifted to a lower field, and C-2′ (*δ*_C_ 75.5) was shifted to an upper field compared with those in **4** (C-3′ (*δ*_C_ 86.9), C-2′ (*δ*_C_ 85.5)), which suggested that the C-3′ hydroxyl group of the glucose was substituted. The HMBC correlation of H-3′ (*δ*_H_ 3.38) to C-12″ (*δ*_C_ 110.6) and H-12″ (*δ*_H_ 4.78) to C-3′ further supported the above deduction. For compound **6**, C-4′ (*δ*_C_ 75.8) of the glucose was shifted to a lower field, and C-2′ (*δ*_C_ 73.9) was shifted to an upper field compared with those in **4** (C-4′ (*δ*_C_ 65.4) and C-2′ (*δ*_C_ 85.5)), suggesting that the C-4′ hydroxyl group of the glucose was substituted. The HMBC correlation of H-4′ (*δ*_H_ 3.98) to C-12″ (*δ*_C_ 110.6) and H-12″ (*δ*_H_ 4.85) to C-4′ also supports this deduction. The ^1^H-^1^H COSY, HSQC, and HMBC experiments allowed for the complete assignment for structures **5** and **6,** respectively (Figure 2).

All of the isolated compounds (**1**–**9**) were evaluated for antibacterial activity (against *Bacillus subtilis*, *Staphylococcus aureus*, methicillin-resistant *Staphylococcus aureus* (MRSA), *Pseudomonas aeruginosa*, and *Salmonella paratyphi*), cell growth inhibition (against A549, Hela, HepG2, and CCRF-CEM cancer cell lines), and inhibition of LPS-induced NO production in RAW264.7 macrophages. As shown in Table 3, compounds **1**, **2**, and **7**–**9** exhibited antibacterial activity against *S. aureus* and *B. subtilis* (MIC 4−16 μg/mL), compounds **1**–**9** displayed low cytotoxicity on the CCRF-CEM cell lines (IC_50_ 10.44–27.40 µM); and compounds **1**, **2**, and **5** showed weak inhibition against LPS-induced NO production (IC_50_ 21.56–28.06 μM). The antibacterial activity of lemnaboursides disappeared if the glucose was condensed with the nardosinane moiety. Moreover, lemnabourside G (tri-acetylation of the sugar at different hydroxyl groups) was also inactive. It can be concluded that steric hindrance may decrease the antibacterial activity of lemnaboursides, however, it seems to not affect the weak cytotoxic activity (against CCRF-CEM cell lines).

## 3. Materials and Methods

### 3.1. General Experimental Procedures

Optical rotations were measured on a Jasco P-1010 Polarimeter (JASCO, Tokyo, Japan) in MeOH at 20 °C and UV spectra were measured on a ThermoFisher Evolution 201/220 spectrophotometer (Thermo Scientific, Waltham, MA, USA). NMR spectra were recorded on a Bruker AVANCE NEO 600 spectrometer (BrukerBiospin AG, Fällanden, Germany). ^1^H chemical shifts were referenced to the residual CDCl_3_ (7.26 ppm) and ^13^C chemical shifts were referenced to the CDCl_3_ (77.2 ppm) solvent peaks. High-resolution electrospray ionization mass spectra (HRESIMS) were performed on an Agilent 6230 TOF LC/MS system (Agilent Technologies Inc., Palo Alto, CA, USA) and Thermo Scientific TM Q Exactive PlusTM (Thermo Scientific, Waltham, MA, USA). Reversed-phase HPLC purifications were performed on a Waters 1525 binary HPLC pump attached to a Waters 2998 photodiode array detector (Waters) using a preparative Cosmosil ODS column (250 mm × 20.0 mm i.d., 5 µm, Cosmosil, Nakalai Tesque Co. Ltd., Kyoto, Japan). Column chromatography was performed on silica gel (Qingdao Haiyang Chemical Co. Ltd., Qingdao, China) and YMC reversed-phase silica gel (50 μm, YMC Co. Ltd., Kyoto, Japan). Precoated silica gel plates (HSGF-254, Qingdao Haiyang Chemical Co. Ltd., Qingdao, China) were used for analytical thin-layer chromatography (TLC). Spots were detected on TLC by heating after spraying with a sulfuric acid reagent.

### 3.2. Animal Material

Specimens of the soft coral *Lemnalia bournei* were collected from the coast of Xisha Island in the South China Sea by SCUBA diving at a depth of 7m, and frozen immediately after collection. The specimen was identified by Prof. Ping-Jyun Sung (National Museum of Marine Biology and Aquarium). The fresh sample was shown in Figure S58. The spicules from the cortex of the basal part of the stem, the cortex of the distal part of the stem, and the tentacles were extracted, then observed under a microscope (Figure S59). A voucher specimen (XSSC201915) was deposited at Li Dak Sum Yip Yio Chin Kenneth Li Marine Biopharmaceutical Research Center, Department of Marine Pharmacy, College of Food and Pharmaceutical Sciences, Ningbo University, Ningbo, China.

### 3.3. Extraction and Isolation

The frozen soft coral (wet mass 2.5 Kg) was cut into pieces and freeze-dried, then extracted with acetone six times under ultrasound condition. The acetone extract was concentrated, and then partitioned between Et_2_O and H_2_O. Evaporation of Et_2_O in vacuo yielded a residue of 60.3 g. The Et_2_O fraction (60.3 g) was subjected to silica gel column, eluting with a gradient of petroleum ether/EtOAc (from 50:0 to 1:1, *v:v*) to obtain five fractions (Fr.1–Fr.5). Fr.4 was mainly compound **7** (14.0 g). Fr.3 (30.0 g) was subjected to a ODS CC (MeOH/H_2_O, from 40:60 to 100:0, *v/v*) to give eight fractions. Fr.3.5 (6.0 g) was further separated on a silica gel CC (petroleum ether/EtOAc, from 10:1 to 1:1, *v/v*) to give nine fractions (Fr.3.5.1–Fr.3.5.9.). Fr.3.5.2 was purified by semipreparative HPLC (MeOH/H_2_O, 96:4, *v/v*) to yield **8** (16.0 mg) and **9** (13 mg). Fr.3.5.6 (124.0 mg) was purified by preparative HPLC (MeOH/H_2_O, 94:6) to afford **3** (33.0 mg). Fr.3.5.7 (75.0 mg) was purified by HPLC (MeOH/H_2_O, 95:5, *v/v*) to afford **2** (45.0 mg). Fr.3.5.8 (31.0 mg) was purified by semipreparative HPLC (MeOH/H_2_O, 94:6, *v/v*) to afford **1** (19.0 mg). Fr.3.7 (1.2 g) was subjected to a silica gel CC (petroleum ether/EtOAc, from 20:1 to 2:1, *v/v*) to give seven fractions, Fr.3.7.1–Fr.3.7.7. The subfraction Fr.3.7.4 (320.0 mg) was purified by preparative HPLC (MeCN/H_2_O, 97:3, *v/v*) to yield **3** (54.5 mg), **4** (20.0 mg), and **5** (32.0 mg).

Lemnabourside E (**1**): amorphous solid; {${\left[\alpha \right]}_{D}^{25}$ +33.0 (*c* 0.10, MeOH)}; UV (MeOH) *λ*_max_ (log ε) 204 (1.85) nm; ^1^H and ^13^C NMR spectroscopic data, see Table 1 and Table 2; HRESIMS *m/z* 515.2977 [M + Na]^+^ (calcd for C_28_H_44_O_7_Na, 515.2980).

Lemnabourside F (**2**): amorphous solid; {${\left[\alpha \right]}_{D}^{25}$ +20.0 (*c* 0.10, MeOH)}; UV (MeOH) *λ*_max_ (log ε) 204 (2.06) nm; ^1^H and ^13^C NMR spectroscopic data, Table 1 and Table 2; HRESIMS *m/z* 559.3246 [M + Na]^+^ (calcd for C_30_H_48_O_8_Na, 559.3242).

Lemnabourside G (**3**): amorphous solid; {${\left[\alpha \right]}_{D}^{25}$ +13.0 (*c* 0.10, MeOH)}; UV (MeOH) *λ*_max_ (log ε) 203 (2.13) nm; ^1^H and ^13^C NMR spectroscopic data, Table 1 and Table 2; HRESIMS *m/z* 601.3336 [M + Na]^+^ (calcd for C_32_H_50_O_9_Na, 601.3348).

Lemnadiolbourside A (**4**): amorphous solid; {${\left[\alpha \right]}_{D}^{25}$ +46.0 (*c* 0.10, MeOH)}; UV (MeOH) *λ*_max_ (log ε) 204 (2.53) nm; for ^1^H and ^13^C NMR spectroscopic data, see Table 1 and Table 2; HRESIMS *m/z* 707.4481 [M + Na]^+^ (calcd for C_41_H_64_O_8_Na, 707.4494).

Lemnadiolbourside B (**5**): amorphous solid; {${\left[\alpha \right]}_{D}^{25}$ +76.0 (*c* 0.10, MeOH)}; UV (MeOH) *λ*_max_ (log ε) 204 (2.44) nm; ^1^H and ^13^C NMR spectroscopic data, Table 1 and Table 2; HRESIMS *m/z* 707.4483 [M + Na]^+^ (calcd for C_41_H_64_O_8_Na, 707.4494).

Lemnadiolbourside C (**6**): amorphous solid; {${\left[\alpha \right]}_{D}^{25}$ +25.0 (*c* 0.10, MeOH)}; UV (MeOH) *λ*_max_ (log ε) 203 (2.28) nm; ^1^H and ^13^C NMR spectroscopic data, Table 1 and Table 2; HRESIMS *m/z* 707.4478 [M + Na]^+^ (calcd for C_41_H_64_O_8_Na, 707.4494).

Lemnabourside (**7**): white solid; {${\left[\alpha \right]}_{D}^{25}$ +35.0 (*c* 0.10, MeOH)}; this structure was deduced by comparison with ^1^H and ^13^C NMR literature data of the known compound as well as the sign of its specific rotation [9].

Lemnabourside B (**8**): white solid; {${\left[\alpha \right]}_{D}^{25}$ +27.0 (*c* 0.10, MeOH)}; this structure was deduced by comparison with ^1^H and ^13^C NMR literature data of the known compound as well as the sign of its specific rotation [9].

Lemnabourside C (**9**): white solid; {${\left[\alpha \right]}_{D}^{25}$ +33.0 (*c* 0.10, MeOH)}; this structure was deduced by comparison with the ^1^H and ^13^C NMR literature data of the known compound as well as the sign of its specific rotation [9].

### 3.4. Acid Hydrolysis of Compounds ***1**–**6***

A solution of compound (3.0 mg) dissolved in dioxane (0.5 mL) and in 1 N H_2_SO_4_ (0.5 mL) was refluxed (4 h), cooled, and extracted with CH_2_Cl_2_ (3 × 2 mL). The CH_2_Cl_2_ layer was condensed and washed with 5% NaHCO_3_ and H_2_O five times respectively, and then purified on Si gel preparative thin layer chromatography [petroleum ether/EtOAc, 30:1, *v/v*] to give a diterpene aldehyde (for **1**, **4**–**6**) or diterpene alcohol (for **2** and **3**). The aqueous layer was neutralized with 5% NaOH to pH 7 and then condensed as white solids to yield D-glucose. The D-glucose product and L-cysteine methyl ester hydrochloride (5 mg) was dissolved in pyridine (0.5 mL) and heated at 60 °C for 60 min, and then phenylisothiocyanate (5 µL) was added to the mixture and heated at 60 °C for 60 min. The reaction mixture was analyzed by UPLC and detected at 250 nm, 25 °C, using a Waters BEH C_18_ (1.7 μm, 2.1 × 100 mm) column, eluting with MeCN/H_2_O (from 10:90 to 0:100, *v/v*, 0–10 min). Peaks of the glucose derivatives were detected by comparison with retention time. Standard d-glucose was treated in the same way as the sample.

### 3.5. Computational Methods

The theoretical electronic circular dichroism (ECD) spectra of the isolated compounds were calculated with the Gaussian09 program package based on the relative configurations determined by their 1D-NOE spectra (Gaussian Inc., Wallingford, CT, USA). Conformational analyses and density functional theory (DFT) calculations were used to generate and optimize the conformers at the B3LYP/6–31+G(d,p) level of theory as the method described in a reported article [14].

### 3.6. Antibacterial Assays

The antibacterial activities were evaluated with the broth dilution assay [15]. Five bacterial strains, *Bacillus subtilis* [CMCC (B) 63501], *Staphylococcus aureus* [CMCC (B) 26003], MRSA [ATCC43300]), *Pseudomonas aeruginosa* [CMCC (B) 10104], and S*almonella paratyphi* A [CMCC (B) 50071] were used, and gentamicin as a positive control.

### 3.7. Cytotoxic Activity Assay

Cell lines were purchased from the American Type Cultural Collection (ATCC, Manassas, VA, USA). The cytotoxicity of the compounds was evaluated against the A549, HepG2, Hela, and CCRF-CEM with the Cell Counting Kit-8 (CCK-8) as the reported method [16]. Chidamide was used as the positive control.

### 3.8. Inhibition of Nitric Oxide Production Assay

The inhibition of the nitric oxide production assay was conducted as a previously reported protocol [17]. Dexamethasone in DMSO was used as the positive control.

## 4. Conclusions

In the course of the exploration of bioactive secondary metabolites from marine organisms, six new bicyclic diterpene glycosides (**1**–**6**) and three known lemnaboursides (**7**–**9**) were isolated and characterized from soft coral *L. bournei*. Based on the ECD spectroscopic data and the biogenetic consideration, the absolute configuration of the new compounds could be determined. In bioassay, compounds **1**, **2**, and **4**–**6** exhibited antibacterial activity against *S. aureus* and *B. subtilis*; compounds **1**–**9** displayed low cytotoxicity on CCRF-CEM cell lines; and compounds **1**, **2**, and **5** showed weak inhibition against LPS-induced NO production. The antibacterial activity of lemnabourside G (tri-acetylated glucose) and lemnadiolboursides disappeared, which indicated the steric hindrance may decrease the antibacterial activity of lemnaboursides. It is worth noting that the discovery of compounds **1**–**6**, once again, enriched the chemical diversity and complexity of diterpene glycosides from marine organisms, and will stimulate further pharmacological studies due to their intriguing structural features and potent biological activities. As the main component of *L. bournei*, further pharmacological investigations of lemnabourside are still worth pursuing. 

## Figures and Tables

**Table 1:** ^1^H NMR spectroscopic data (600 MHz, CDCl_3_) for compounds **1**–**6**.

Position	1	2	3	4	5	6
	*δ*_H_, (*J* in Hz)	*δ*_H_, (*J* in Hz)	*δ*_H_, (*J* in Hz)	*δ*_H_, (*J* in Hz)	*δ*_H_, (*J* in Hz)	*δ*_H_, (*J* in Hz)
1a	1.37, m	1.38, m ^a^	1.36, m ^a^	1.36, m ^a^	1.35, m ^a^	1.35, m
1b	1.17, m ^a^	1.21, m ^a^	1.20, m ^a^	1.36, m ^a^	1.35, m ^a^	1.16, m ^a^
2a	1.98, m ^a^	1.98, dd (11.8, 6.3)	1.98, m	2.00, m ^a^	1.97, m ^a^	1.97, m
2b	1.90, brd (5.1)	1.91, dd (17.0, 4.6)	1.92, m ^a^	1.92, m ^a^	1.93, m ^a^	1.92, m ^a^
4	5.48, brd (3.7)	5.48, m	5.48, m	5.48, m	5.48, brd (3.0)	5.49, brd (3.6)
5	2.04, m	2.02, dt (10.4, 4.5)	2.04, m ^a^	2.02, m ^a^	2.03, m ^a^	2.03, m ^a^
6	1.50, m ^a^	1.49, m	1.49, m	1.50, m	1.50, m	1.49, dt (10.0, 5.8)
7a	1.85, m	1.84, m	1.84, m	1.83, m	1.84, m	1.84, m
7b	1.77, m ^a^	1.77, m ^a^	1.77, m ^a^	1.76, m ^a^	1.78, m ^a^	1.76, m ^a^
8	5.40, brt (3.6)	5.40, brt (3.6)	5.40, brt (3.6)	5.40, brs	5.41, brs	5.41, brs
10	1.93, m ^a^	1.93, m ^a^	1.93, m ^a^	1.93, m ^a^	1.92, m ^a^	1.93, m ^a^
11	1.77, m ^a^	1.77, m ^a^	1.77, m ^a^	1.77, m ^a^	1.77, m ^a^	1.76, m ^a^
12a	1.21, m ^a^	1.20, m ^a^	1.21, m ^a^	1.22, m ^a^	1.21, m ^a^	1.20, m ^a^
12b	1.15, m ^a^	1.14, m	1.15, m	1.14, m ^a^	1.14, m ^a^	1.13, m ^a^
13	1.36, m ^a^	1.37, m ^a^	1.36, m ^a^	1.35, m ^a^	1.36, m	1.35, m
14a	1.48, m ^a^	1.37, m ^a^	1.37, m ^a^	1.45, m ^a^	1.47, m	1.45, dt (11.4, 5.6)
14b	1.09, m	1.08, brq (8.5)	1.09, m	1.08, m	1.08, m	1.09, m
15	1.69, m	1.75, m ^a^	1.76, m ^a^	1.65, m	1.70, m	1.68, m ^a^
16a	4.60, d (4.9)	3.68, dd (9.4, 6.2)	3.68, dd ^a^	4.59, d (4.6)	4.58, d (5.1)	4.60, d (5.1)
16b		3.38, dd (9.4, 6.2)	3.38, dd(9.4, 7.9)			
17	1.69, s	1.67, s	1.69, s	1.69, s	1.69, s	1.68, s
18	1.68, s	1.69, s	1.68, s	1.68, s	1.69, s	1.68, s
19	0.81, d (6.8)	0.81, d (6.8)	0.81, d (6.8)	0.81, d (6.8)	0.81, d (6.7)	0.80, d (6.8)
20	0.92, d (6.8)	0.91, d (6.7)	0.91, d (6.7)	0.91, d (6.8)	0.91, d (6.6)	0.90, d (6.8)
1′	4.93, s	4.31, d (7.7)	4.34, d (7.8)	4.88, s	4.94, s	5.01, s
2′	4.56, d (4.9)	3.49, dd ^a^	3.57, dd (9.4, 7.9)	3.36, d (5.8)	3.62, d (5.7)	3.66, m
3′	3.75, dd (10.2, 5.3)	4.93, t (9.0)	5.12, t (9.5)	3.71, dd (10.6, 5.8)	3.38, dd (10.5, 5.8)	3.74, m
4′	4.32, dd (10.1, 7.7)	3.52, dd ^a^	5.03, t (9.7)	4.20, dd (10.3, 8.2)	4.22, t (9.1)	3.97, dd (8.1, 5.8)
5′	3.89, d (7.7)	3.51, m ^a^	3.67, m ^a^	3.86, d (7.7)	3.85, d (7.7)	4.04, m
6′a	3.95, d (12.5)	4.43, dd (12.1, 4.2)	4.27, dd (12.1, 4.9)	3.94, d (12.1)	3.92, d (12.6)	4.15, d (12.6)
6′b	3.55, dd (12.6, 1.9)	4.33, dd (11.4, 1.9)	4.12, dd (12.2, 2.4)	3.52, d (11.1)	3.55, dd (12.5)	3.53, dd (12.7, 2.3)
1”				5.53, brd (5.1)	5.56, brd (4.5)	5.53, d (4.9)
2”a				2.00, m ^a^	2.01, m ^a^	2.02, m ^a^
2”b				1.93, m ^a^	1.96, m ^a^	1.93, m ^a^
3”a				1.42, m ^a^	1.41, m	1.41, m
3”b				1.39, m ^a^	1.41, m	1.39, m ^a^
4”				1.70, m ^a^	1.70, m	1.67, m ^a^
6”				1.81, d (11.1)	1.83, d (11.1)	1.74, d (10.6)
8”a				2.08, m	2.03, m ^a^	2.03, m ^a^
8”b				1.89, m ^a^	1.90, m ^a^	1.92, m ^a^
9”a				2.46, brt (11.5)	2.45, brt (14.2)	2.46, brt (12.8)
9”b				2.05, m ^a^	2.07, m ^a^	2.03, m ^a^
11”				2.03, m ^a^	2.06, m	1.94, m ^a^
12”				4.75, d (4.7)	4.78, d (4.5)	4.85, d (4.8)
13”				1.25, d (6.6)	1.28, d (6.7)	1.24, d (6.7)
14”				1.13, s	1.13, s	1.13, s
15”				0.85, d (6.5)	0.86, d (6.5)	0.85, d (6.6)
2′-OAc	2.16, s					
3′-OAc		2.17, s	2.08, s			
4′-OAc			2.03, s			
6′-OAc		2.11, s	2.08, s			

^a^ Overlapped signals.

**Table 2:** ^13^C NMR (150 MHz, CDCl_3_) spectroscopic data for compounds **1**–**6**.

Position	1	2	3	4	5	6
	*δ*C, Type	*δ*C, Type	*δ*C, Type	*δ*c, Type	*δ*C, Type	*δ*C, Type
1	25.2, CH_2_	25.0, CH_2_	24.9, CH_2_	25.1, CH_2_	25.1, CH_2_	25.1, CH_2_
2	30.9, CH_2_	30.9, CH_2_	30.8, CH_2_	30.7, CH_2_	30.8, CH_2_	30.8, CH_2_
3	134.6, C	134.6, C	136.6, C	134.3, C	134.5, C	134.5, C
4	124.0, CH	123.9, CH	123.8, CH	123.7, CH	123.9, CH	123.9, CH
5	36.5, CH	36.4, CH	36.4, CH	36.3, CH	36.4, CH	36.4, CH
6	39.1, CH	39.0, CH	39.0, CH	38.9, CH	38.9, CH	38.9, CH
7	24.7, CH_2_	24.7, CH_2_	24.7, CH_2_	24.5, CH_2_	24.6, CH_2_	24.6, CH_2_
8	121.6, CH	121.4 CH	121.4 CH	121.3, CH	121.5, CH	121.5, CH
9	136.7, C	136.8, C	136.6, C	136.5, C	136.6, C	136.6, C
10	39.7, CH	39.6, CH	39.6, CH	39.4, CH	39.6, CH	39.6, CH
11	32.0, CH	31.8, CH	31.8, CH	31.7, CH	31.8, CH	31.8, CH
12	36.0, CH_2_	35.9, CH_2_	35.9, CH_2_	35.8, CH_2_	35.9, CH_2_	35.9, CH_2_
13	24.7, CH_2_	24.7, CH_2_	24.7, CH_2_	25.1, CH_2_	24.6, CH_2_	24.7, CH_2_
14	31.9, CH_2_	33.9, CH_2_	33.8, CH_2_	31.9, CH_2_	31.8, CH_2_	31.9, CH_2_
15	38.1, CH	33.3, CH	33.3, CH	37.9, CH	35.3, CH	37.9, CH
16	102.0, CH	75.7, CH_2_	75.8, CH_2_	101.3, CH	101.6, CH	102.2, CH
17	24.0, CH_3_	23.9, CH_3_	24.0, CH_3_	23.8, CH_3_	23.9, CH_3_	24.0, CH_3_
18	21.9, CH_3_	21.7, CH_3_	21.8, CH_3_	21.6, CH_3_	21.8, CH_3_	21.8, CH_3_
19	13.5, CH_3_	13.4, CH_3_	13.4, CH_3_	13.2, CH_3_	13.4, CH_3_	13.4, CH_3_
20	14.4, CH_3_	16.9, CH_3_	16.9, CH_3_	14.0, CH_3_	14.5, CH_3_	14.3, CH_3_
1′	98.2, CH	103.0, CH	103.0, CH	100.0, CH	100.4, CH	100.0, CH
2′	79.7, CH	72.1, CH	72.2, CH	85.5, CH	75.5, CH	73.9, CH
3′	74.5, CH	77.4, CH	74.4, CH	74.8, CH	86.9, CH	72.8, CH
4′	66.2, CH	69.1, CH	68.6, CH	65.4, CH	64.7, CH	75.8, CH
5′	79.6, CH	74.2, CH	71.8, CH	79.8, CH	79.8, CH	78.9, CH
6′	67.9, CH_2_	63.2, CH_2_	62.2, CH_2_	67.6, CH_2_	67.6, CH_2_	68.1, CH_2_
1”				123.8, CH	124.0, CH	123.9, CH
2”				25.6, CH_2_	25.6, CH_2_	25.7, CH_2_
3”				26.8, CH_2_	26.7, CH_2_	26.7, CH_2_
4”				35.1, CH	35.3, CH	35.3, CH
5”				40.4, C	40.6, C	40.5, C
6”				59.1, CH	59.3, CH	59.5, CH
7”				107.2, C	107.5, C	106.9, C
8”				36.3, CH_2_	36.4, CH_2_	36.4, CH_2_
9”				28.7, CH_2_	28.9, CH_2_	28.8, CH_2_
10”				138.5, C	138.7, C	138.8, C
11”				42.1, CH	42.2, CH	42.6, CH
12”				109.7, CH	110.6, CH	109.4, CH
13”				19.7, CH_3_	20.2, CH_3_	19.8, CH_3_
14”				20.6, CH_3_	20.7, CH_3_	20.8, CH_3_
15”				16.3, CH_3_	16.5, CH_3_	16.5, CH_3_
2′-OAc	172.2, C					
	21.1, CH_3_					
3′-OAc		172.5, C	170.7, C			
		21.1, CH_3_	20.8, CH_3_			
4′-OAc			169.7, C			
			20.7, CH_3_			
6′-OAc		171.7, C	170.7, C			
		20.9, CH_3_	20.8, CH_3_			

**Table 3:** Antibacterial and antiviral activities of the isolated compounds.

No.	MIC (Against *Staphylococcus aureus*, µg/mL)	MIC (Against *Bacillus subtilis*, µg/mL)	IC_50_ (Against CCRF-CEM Cell Lines, µM)	IC_50_ (Against LPS-Induced NO Production; µM)
**1**	8	4	21.04	24.25
**2**	4	4	12.27	21.56
**3**	>128	64	17.74	>30
**4**	>128	64	17.21	>30
**5**	>128	>128	10.44	28.06
**6**	>128	64	22.56	>30
**7**	8	4	17.59	>30
**8**	4	4	21.04	>30
**9**	8	8	27.40	>30
Gentamicin *^c^*	4	4	—	—
Chidamide *^c^*	—	—	0.83	—
Dexamethasone *^c^*	—	—	—	<0.1

*^c^* Positive control.

## Data Availability

The data presented in this study are available in the Supplementary Materials file associated with this article.

## Supplementary Materials

The following are available online https://www.mdpi.com/article/10.3390/md19060339/s1. Figure S1: Calculated and experimental ECD spectra of compound **1**; Figures S2–S48: HRESIMS, 1D and 2D NMR spectra of all new compounds **1**–**6**; Figures S49–S54: 1D NMR spectra of known compounds **7**–**9**. Figures S55–S56: ^1^H NMR data for the bicyclic diterpene aldehyde aglycon and bicyclic diterpene alcohol aglycon. Figure S57: HPLC chromatograms of the sugar derivatives of compounds 1–6 and the standard D-glucose. Figures S58 and S59: Photos of *Lemnalia bournei.*
