# Supplementary material for: Six New Diterpene Glycosides from the Soft Coral Lemnalia bournei
Source: Mar Drugs. 2021 Jun 14;19(6):339. doi: 10.3390/md19060339 (PMC8231804; doi:10.3390/md19060339)
Supplement: Supplementary file 1 [file marinedrugs-19-00339-s001.zip › marinedrugs-1252891-supplementary.pdf]

Supplementary Material

Six New Diterpene Glycosides from the Soft Coral *Lemnalia bournei*

Xia Yan [1], Han Ouyang [2,*], Te Li [1], Yutong Shi [1], Bin Wu [3], Xiaojun Yan [1] and Shan He [1,*]

[1] Li Dak Sum Yip Yio Chin Kenneth Li Marine Biopharmaceutical Research Center, Department of Marine Pharmacy, College of Food and Pharmaceutical Sciences, Ningbo University, Ningbo 315800, Zhejiang, China; yanxia@nbu.edu.cn (X.Y.); telinbu@163.com (T.L.); shiyutong@nbu.edu.cn (X.Y.); yanxiaojun@nbu.edu.cn (X.Y.)

[2] Institute of Drug Discovery Technology, Ningbo University, Ningbo 315211, China

[3] Ocean College, Zhejiang University, Hangzhou 310058, China; wubin@zju.edu.cn

* Correspondence: ouyanghan@nbu.edu.cn (H.O.); heshan@nbu.edu.cn (S.H.); Tel.: +86-574-876-09771 (H.O.); +86-574-876-04382 (S.H.)

Table of Contents

Table of Contents 2

1. Experimental ECD spectrum of 1 and the calculated one 3

2. 1D, 2D NMR and HRMS spectra of the new isolated compounds 3

  2.1 Spectroscopic data for Lemnabourside E (1) 3

  3.2 Spectroscopic data for Lemnabourside F (2).. 10

  3.3 Spectroscopic data for Lemnabourside G (3) 14

  3.4 Spectroscopic data for Lemnadiolbourside H (4).. 19

  3.5 Spectroscopic data for Lemnadiolbourside B (5) 25

  3.6 Spectroscopic data for Lemnadiolbourside C (6) 30

4. NMR spectrums of the known isolated compounds... 36

  4.1 Spectroscopic data for compound 7 36

  4.2 Spectroscopic data for compound 8 37

  4.3 Spectroscopic data for compound 9 39

2. ^{1}H NMR data for bicyclic diterpene aldehyde aglycon and bicyclic diterpene alcohol aglycon 40

6. HPLC chromatograms of the sugar derivatives of compounds 1-6 and the standard D-glucose.. 43

7. Photos of *Lemnalia bournei* underwater and fresh sample after collected...................... 45

8. Photomicrographs of the spicules extracted from *Lemnalia bournei* 45

1. Experimental ECD spectrum of 1 and the calculated one

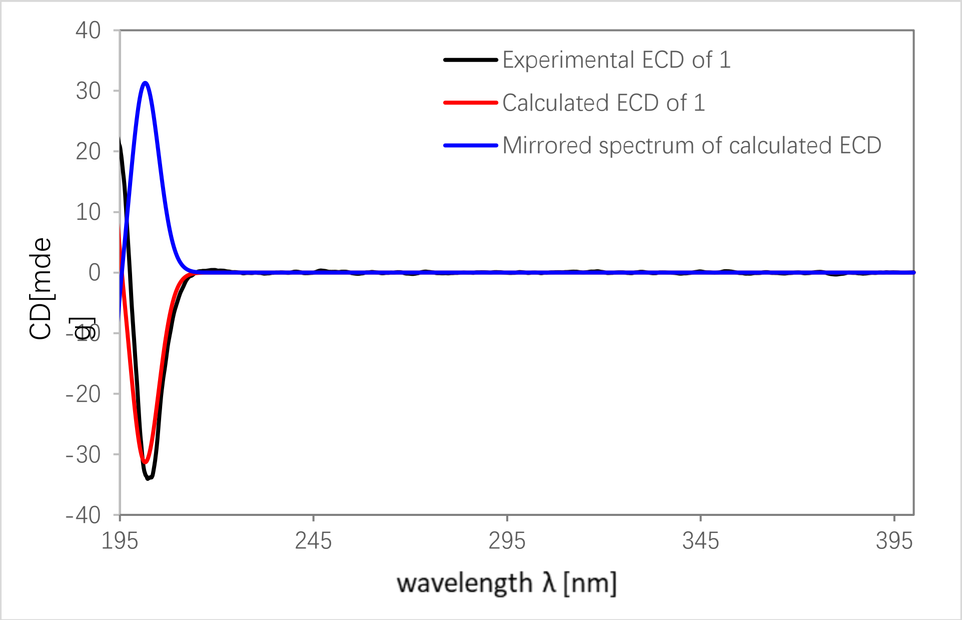

Figure S1 Experimental ECD spectrum of 1 and the calculated ECD spectra of (5*R*,6*S*,10*S*,11*S*)-1 (red) and (5*S*,6*R*,10*R*,11*R*)-1 (blue)

2. 1D, 2D NMR and HRMS spectra of the new isolated compounds

2.1 Spectroscopic data for Lemnabourside E (1)

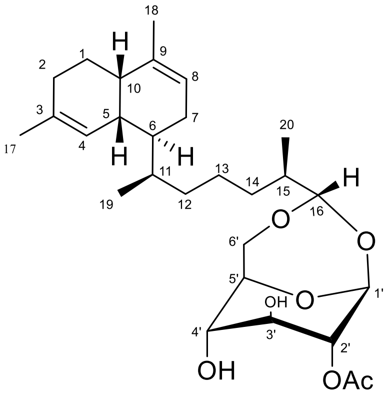

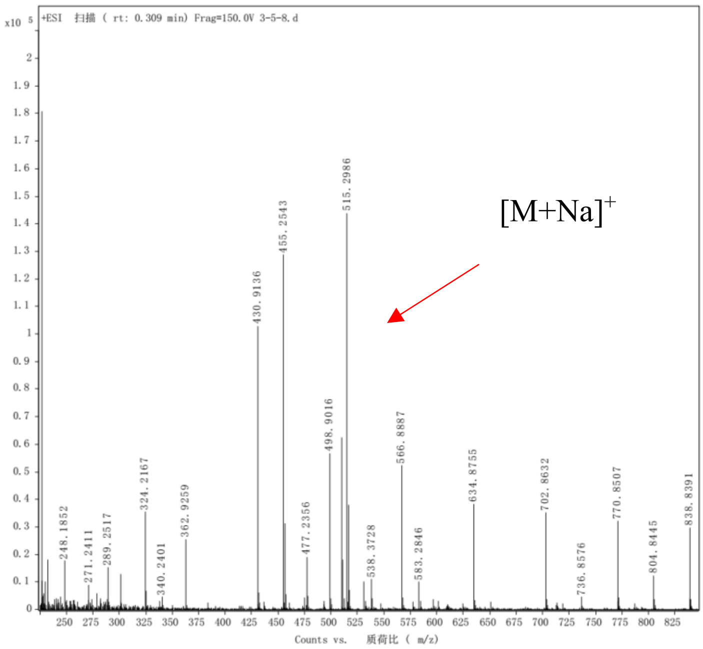

Figure S2 HRESIMS Spectrum of Lemnabourside E (1)

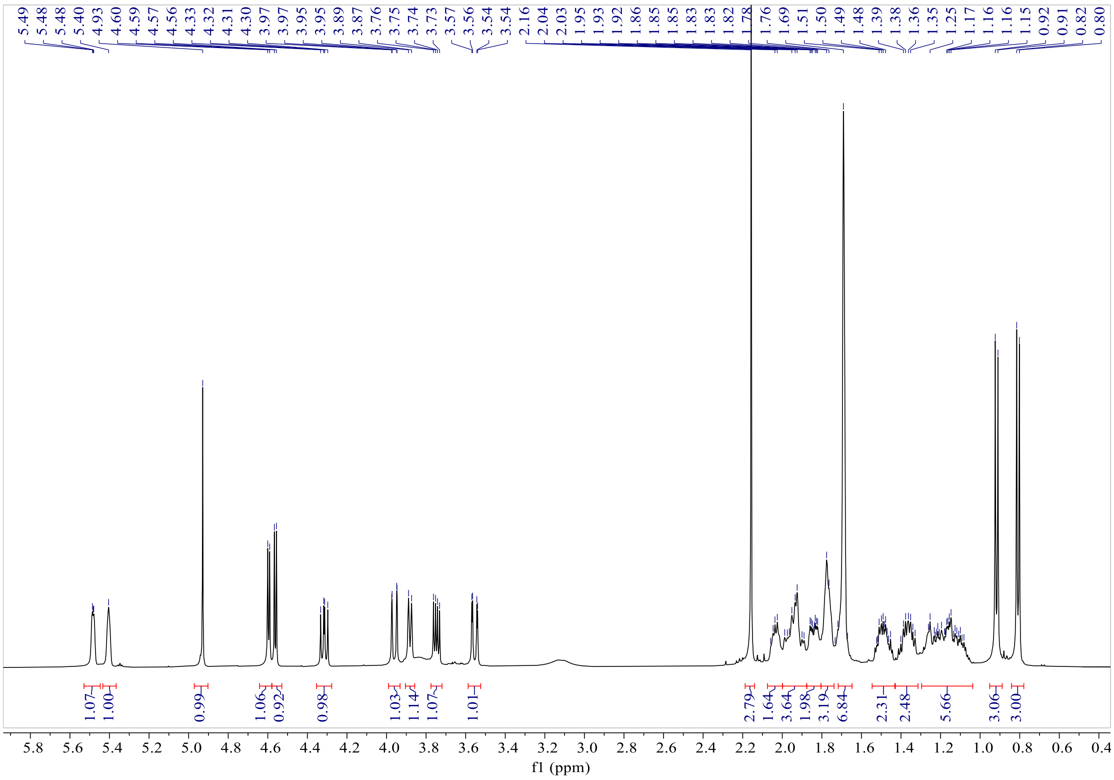

Figure S3 ^{1}H NMR Spectrum of Lemnabourside E (1) (600 MHz; $CDCl_3$)

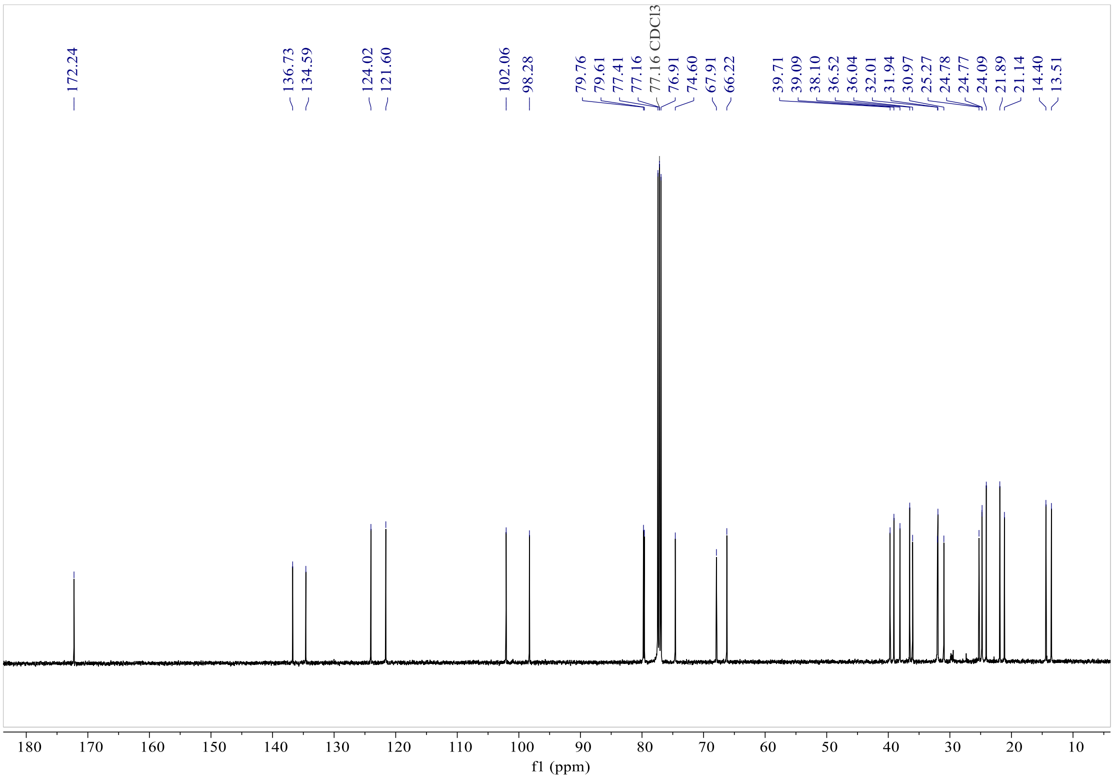

Figure S4 ^{13}C NMR Spectrum of Lemnabourside E (1) (150 MHz; $CDCl_3$)

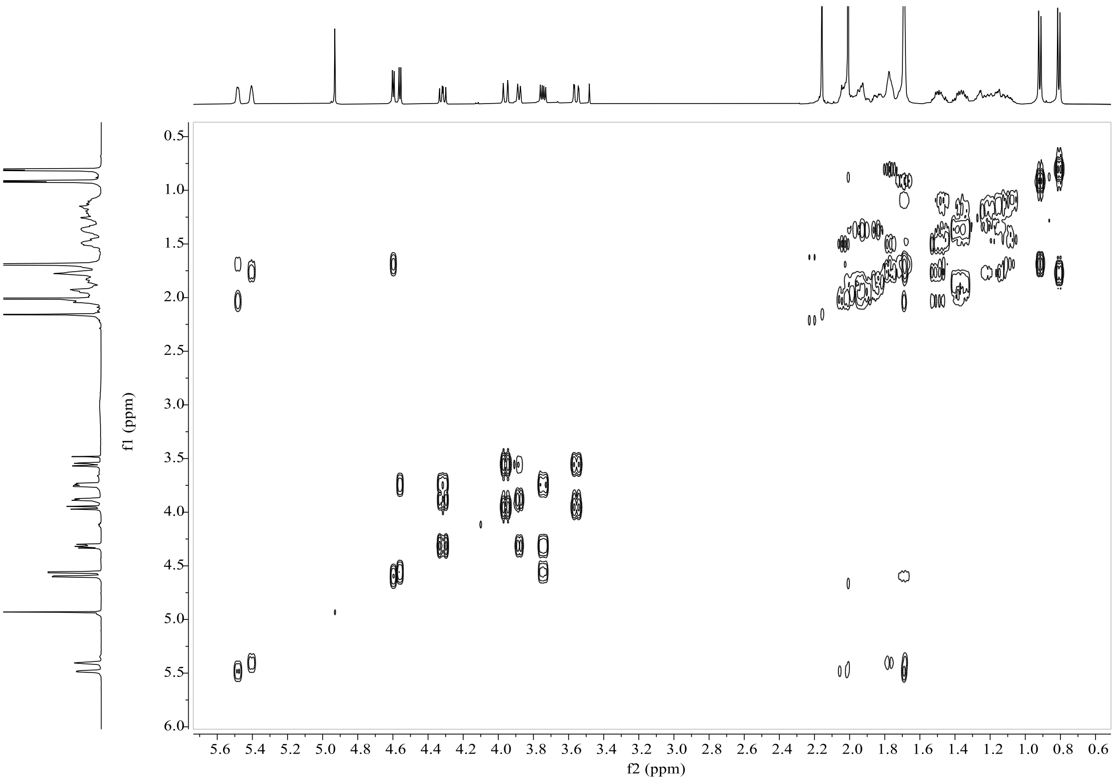

Figure S5 ^{1}H-^{1}H COSY spectrum of Lemnabourside E (1) (600 MHz; $CDCl_3$)

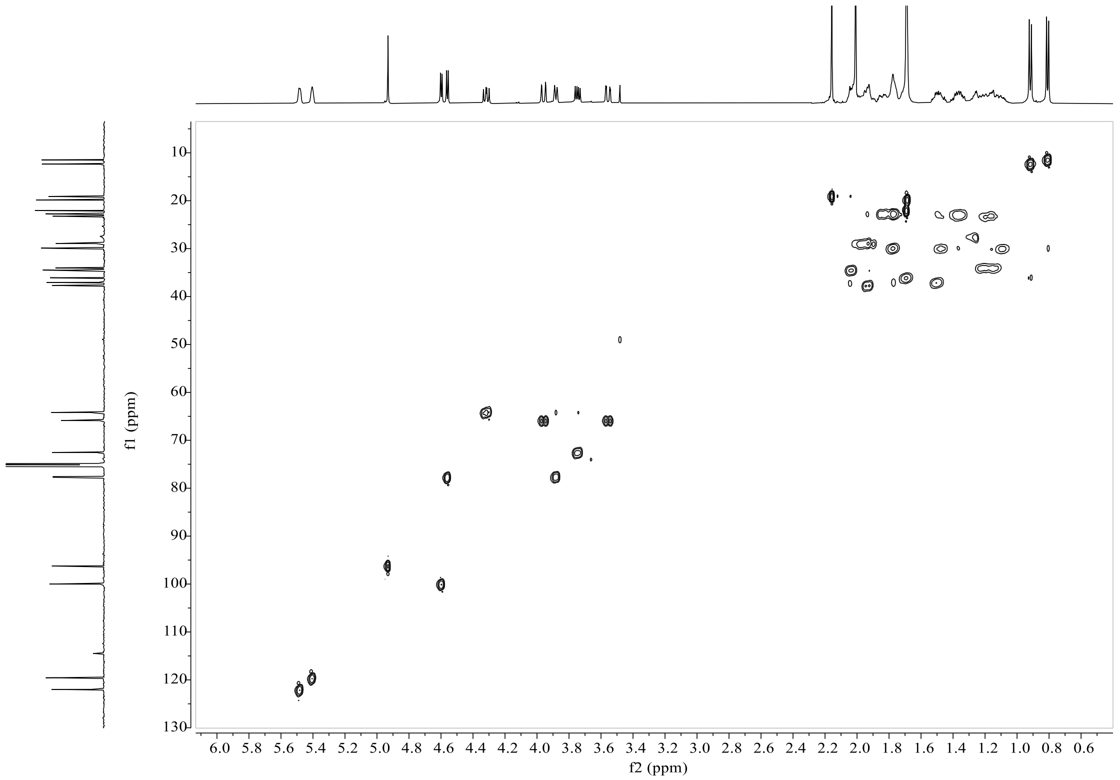

Figure S6 HSQC spectrum of Lemnabourside E (1) (600 MHz; $CDCl_3$)

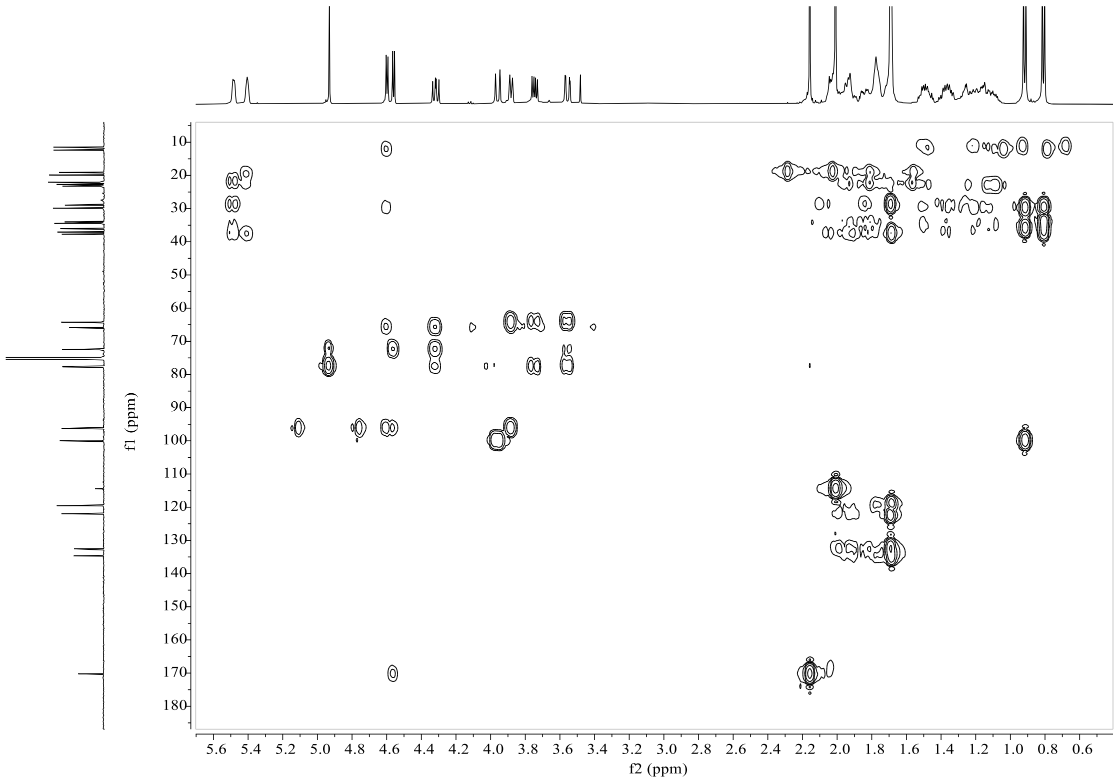

Figure S7 HMBC spectrum of Lemnabourside E (1) (600 MHz; $CDCl_3$)

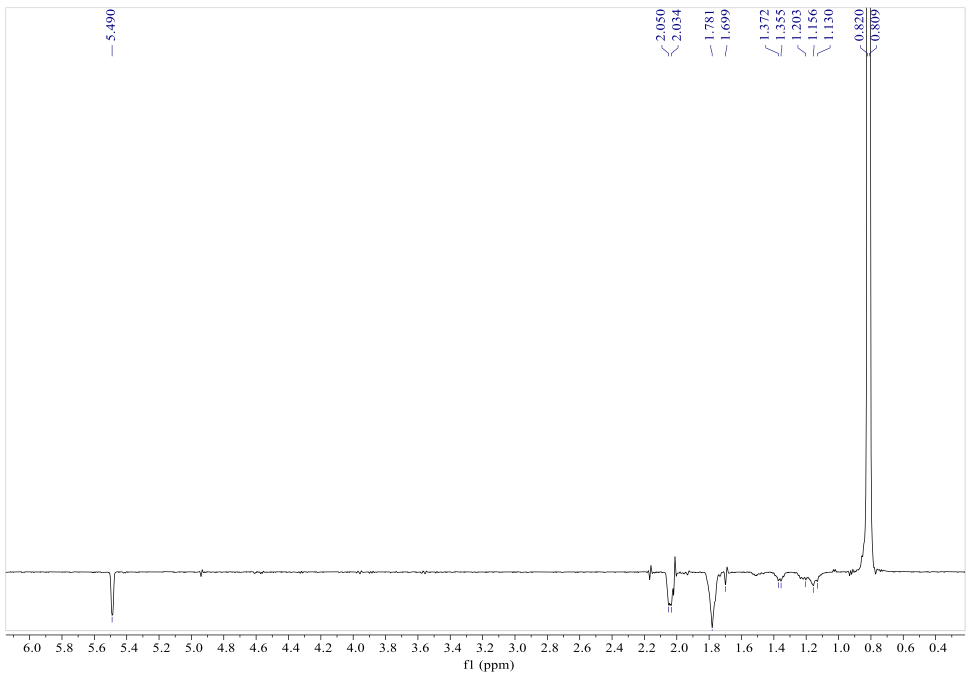
5.490
2.050
2.034
1.781
1.699
1.372
1.355
1.203
1.156
1.130
0.820
0.809
6.0 5.8 5.6 5.4 5.2 5.0 4.8 4.6 4.4 4.2 4.0 3.8 3.6 3.4 3.2 3.0 2.8 2.6 2.4 2.2 2.0 1.8 1.6 1.4 1.2 1.0 0.8 0.6 0.4
f1 (ppm)

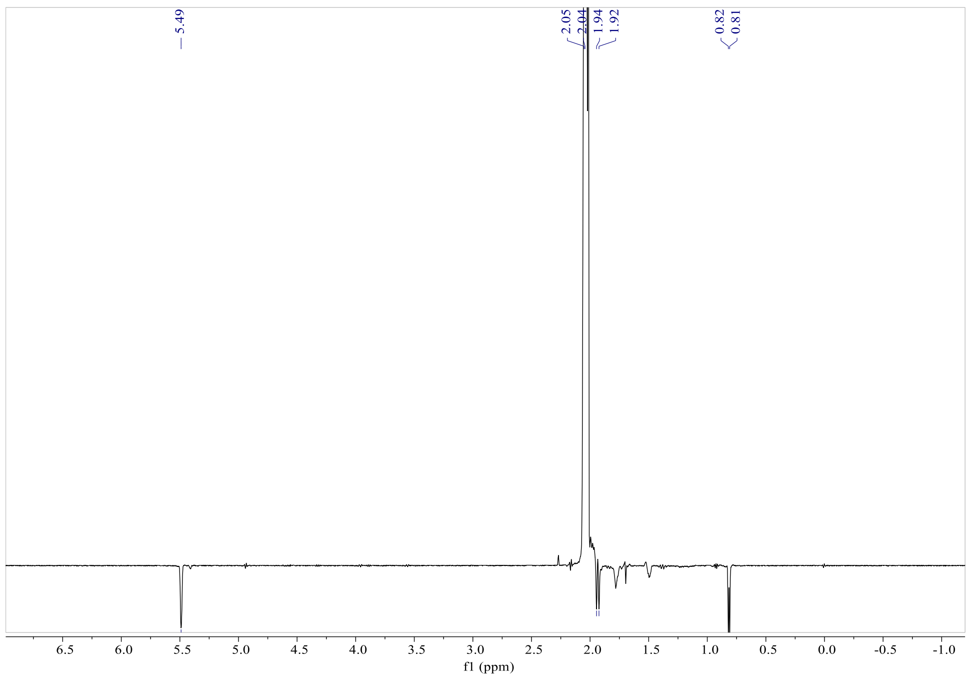
5.49
2.05
2.04
1.94
1.92
0.82
0.81
6.5 6.0 5.5 5.0 4.5 4.0 3.5 3.0 2.5 2.0 1.5 1.0 0.5 0.0 -0.5 -1.0
f1 (ppm)

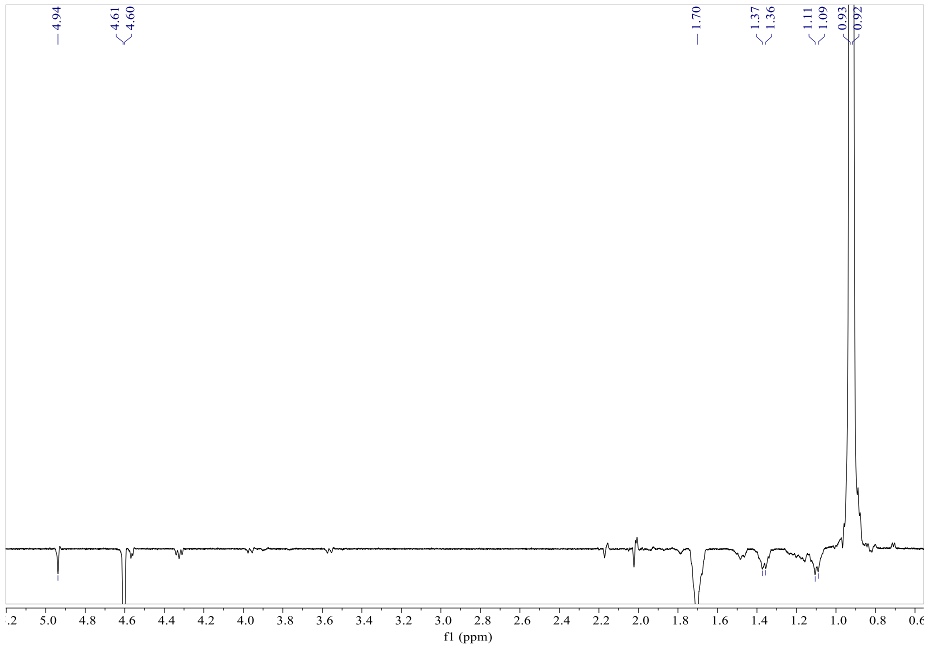
4.94
4.61
4.60
1.70
1.37
1.36
1.11
1.09
0.93
0.92
f1 (ppm)

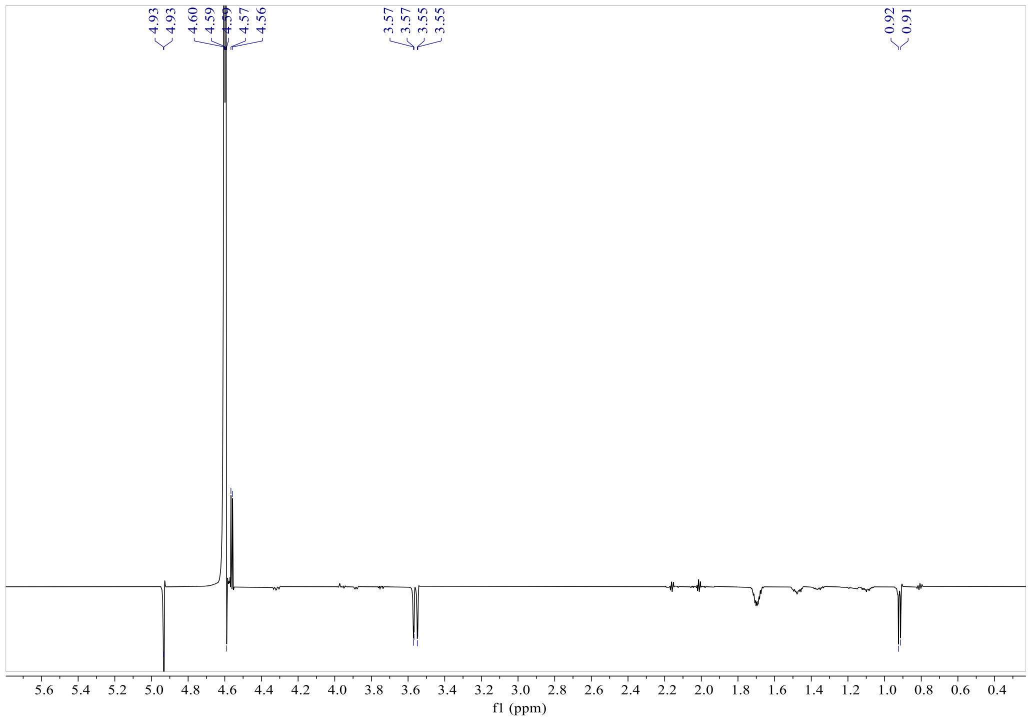
4.93
4.93
4.60
4.59
4.59
4.57
4.56
3.57
3.57
3.55
3.55
0.92
0.91
f1 (ppm)

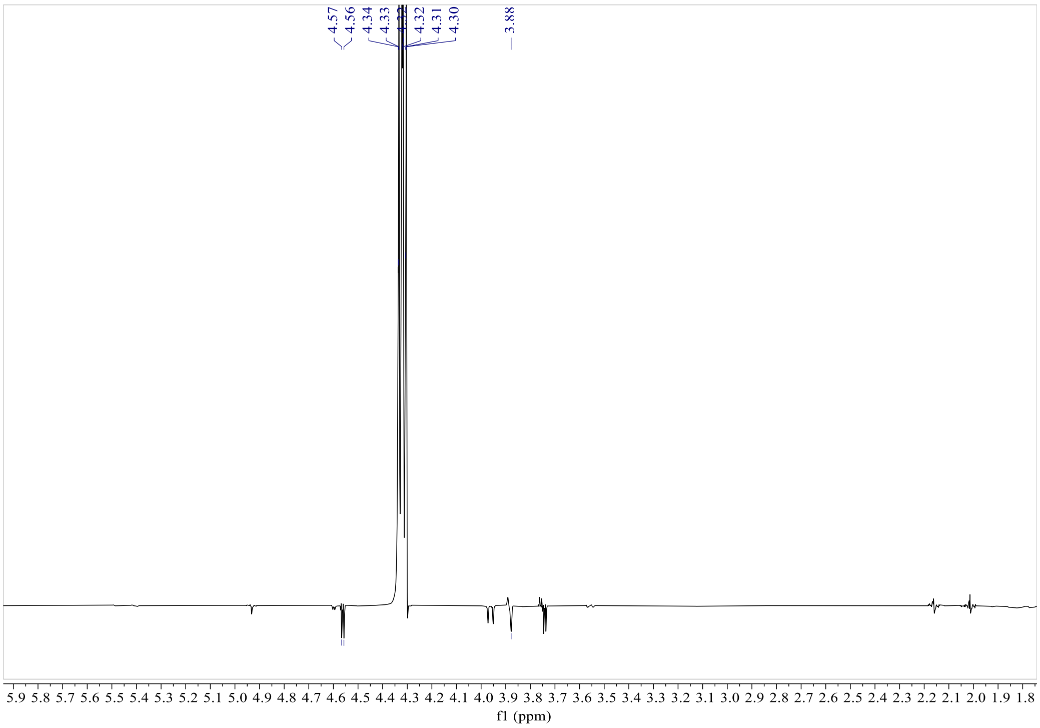

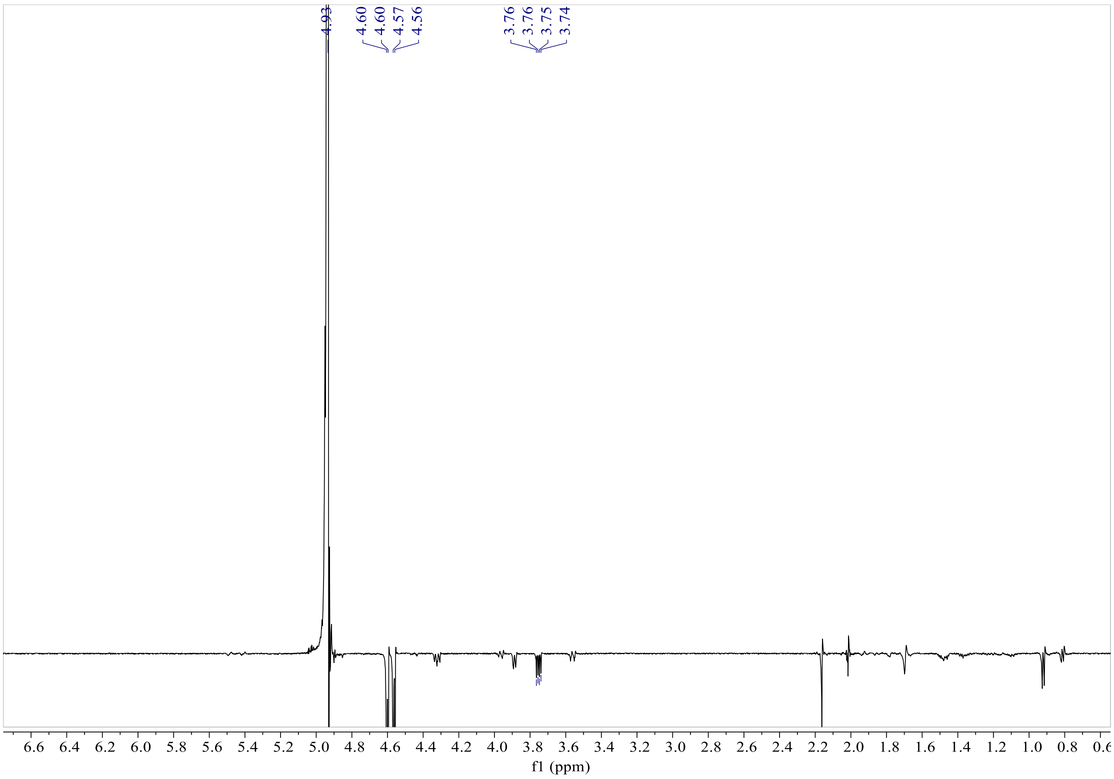

Figure S8 1D-NOE spectrum of Lemnabourside E (1) (600 MHz; $CDCl_3$)

3.2 Spectroscopic data for Lemnabourside F (2)

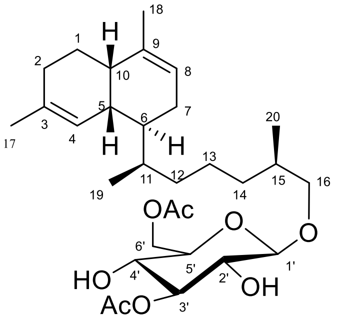

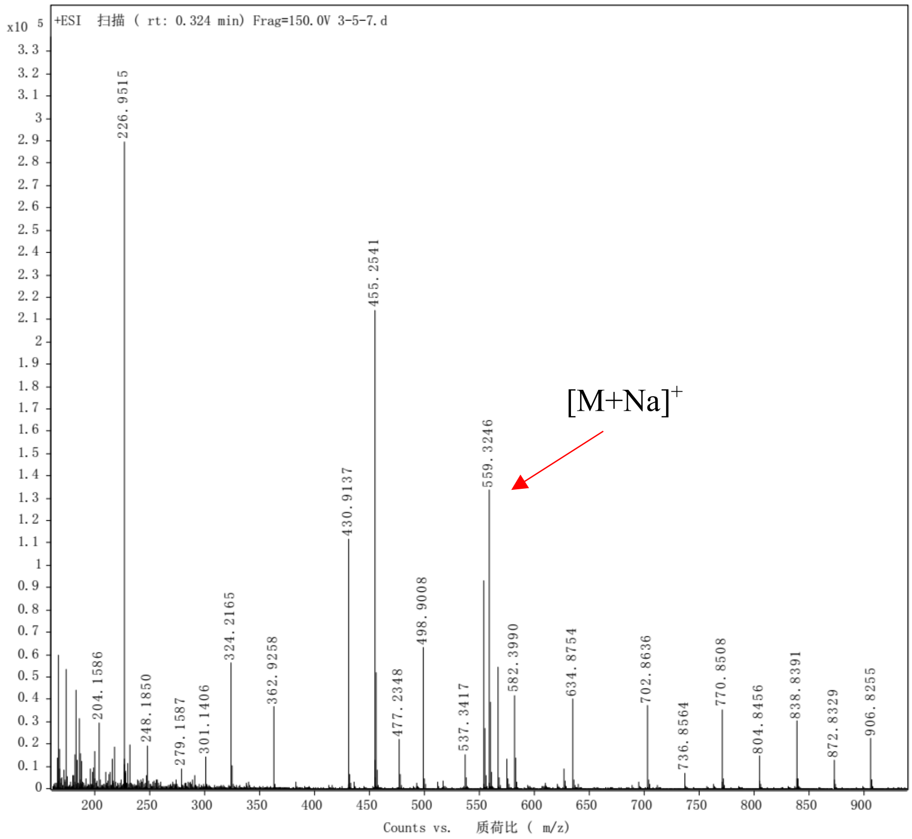

Figure S9 HRESIMS Spectrum of Lemnabourside F (2)

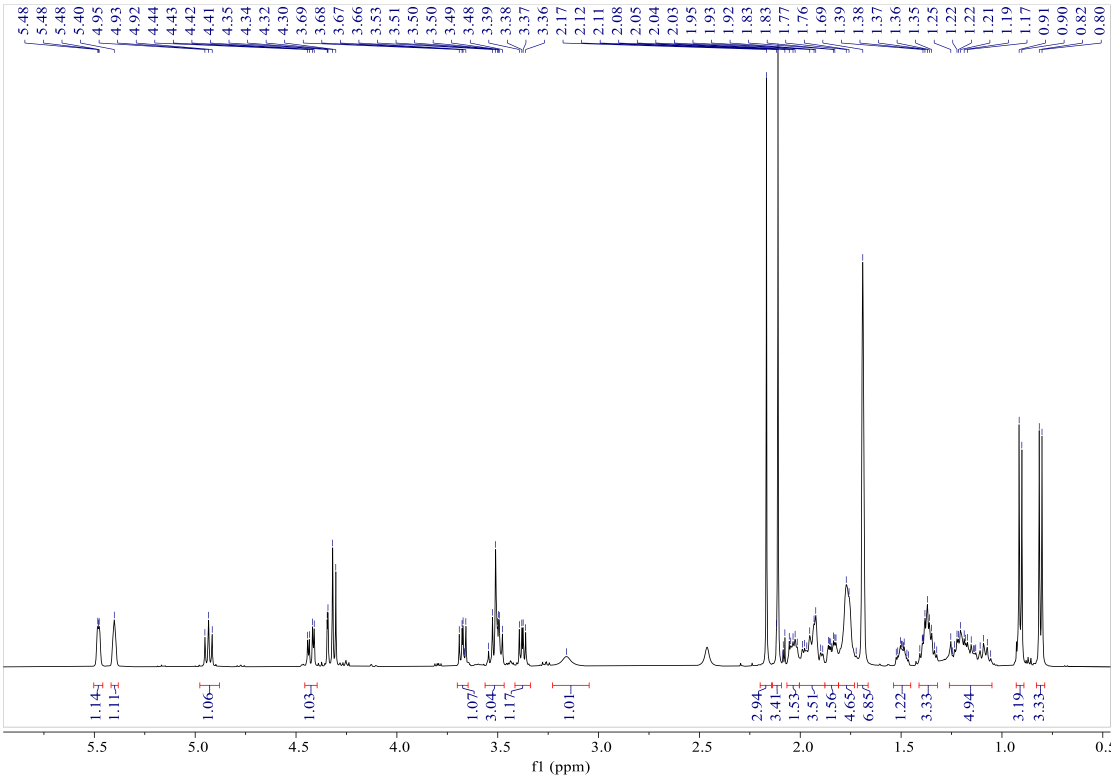

Figure S10 ^{1}H NMR Spectrum of Lemnabourside F (2) (600 MHz; $CDCl_3$)

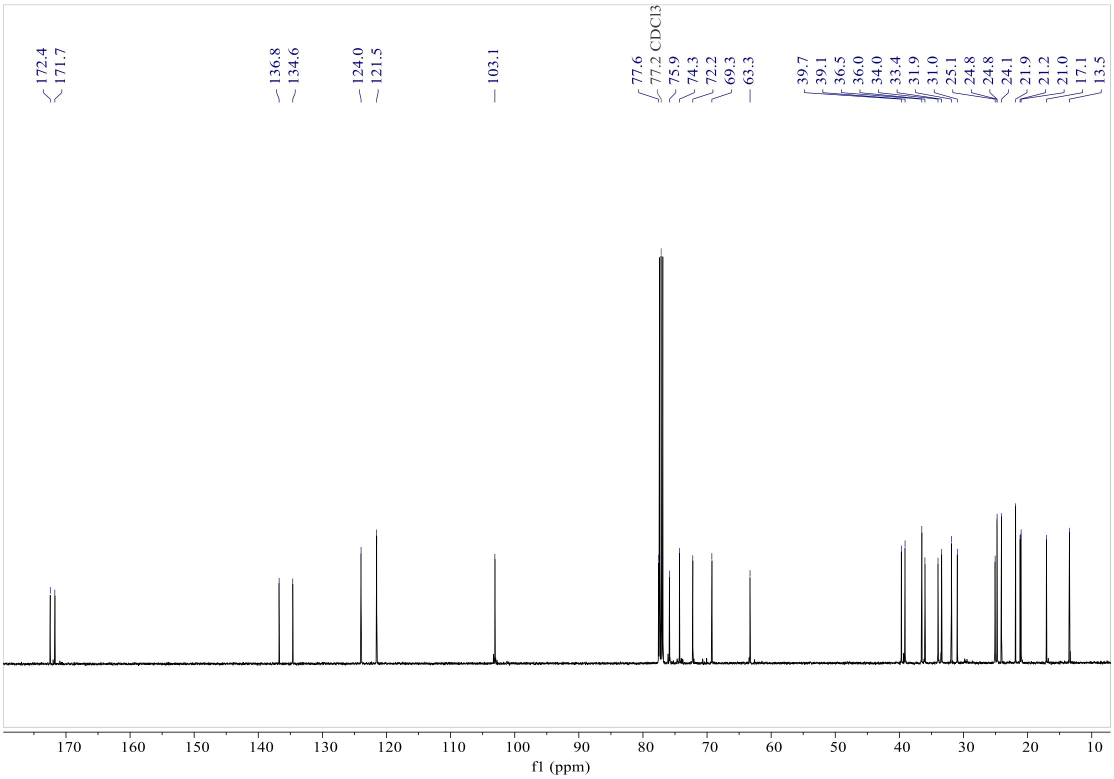

Figure S11 ^{13}C NMR Spectrum of Lemnabourside F (2) (150 MHz; $CDCl_3$)

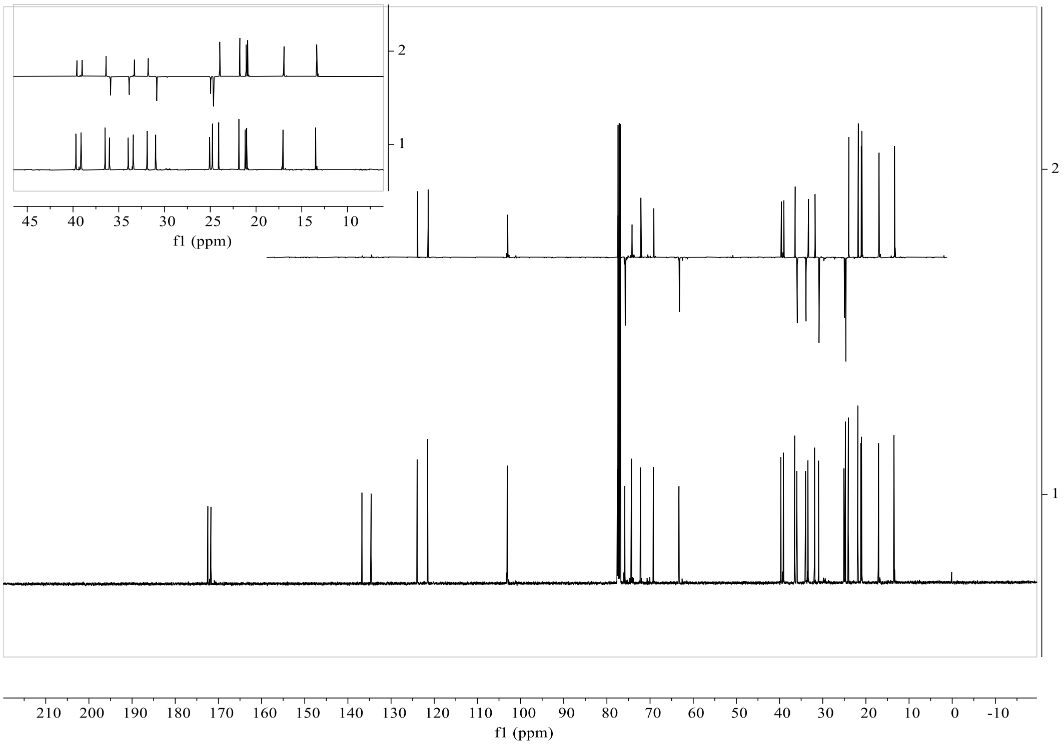

Figure S12 Dept 135° NMR Spectrum of Lemnabourside F (2) (150 MHz; $CDCl_3$)

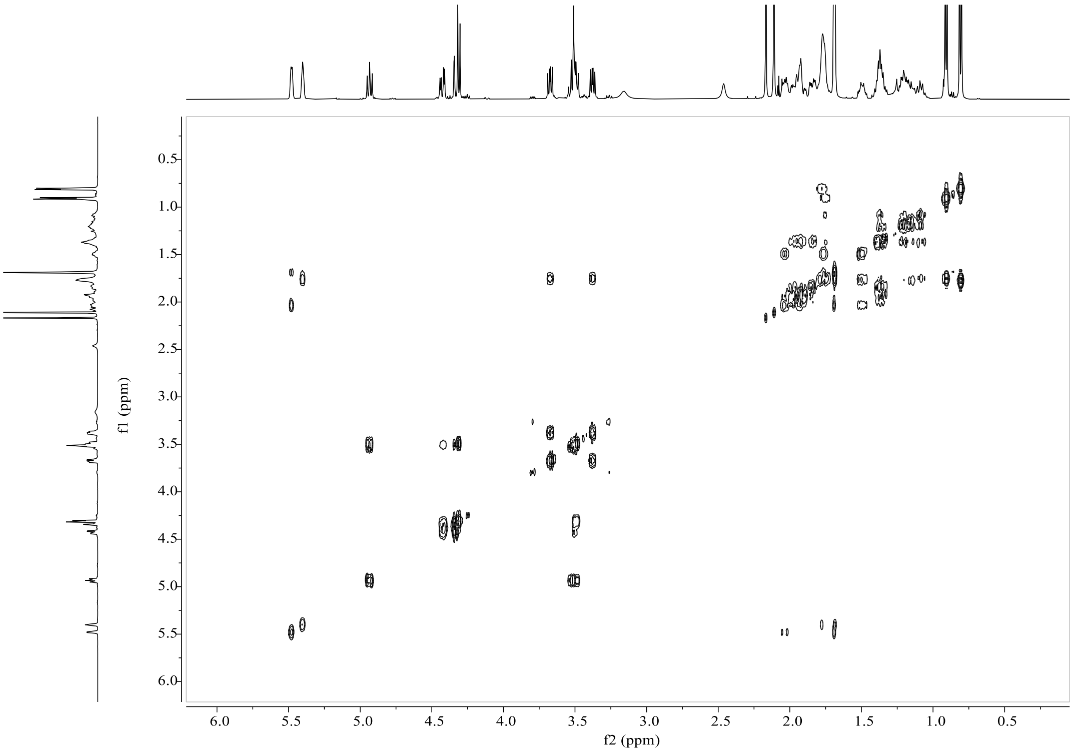

Figure S13 ^{1}H-^{1}H COSY spectrum of Lemnabourside F (2) (600 MHz; $CDCl_3$)

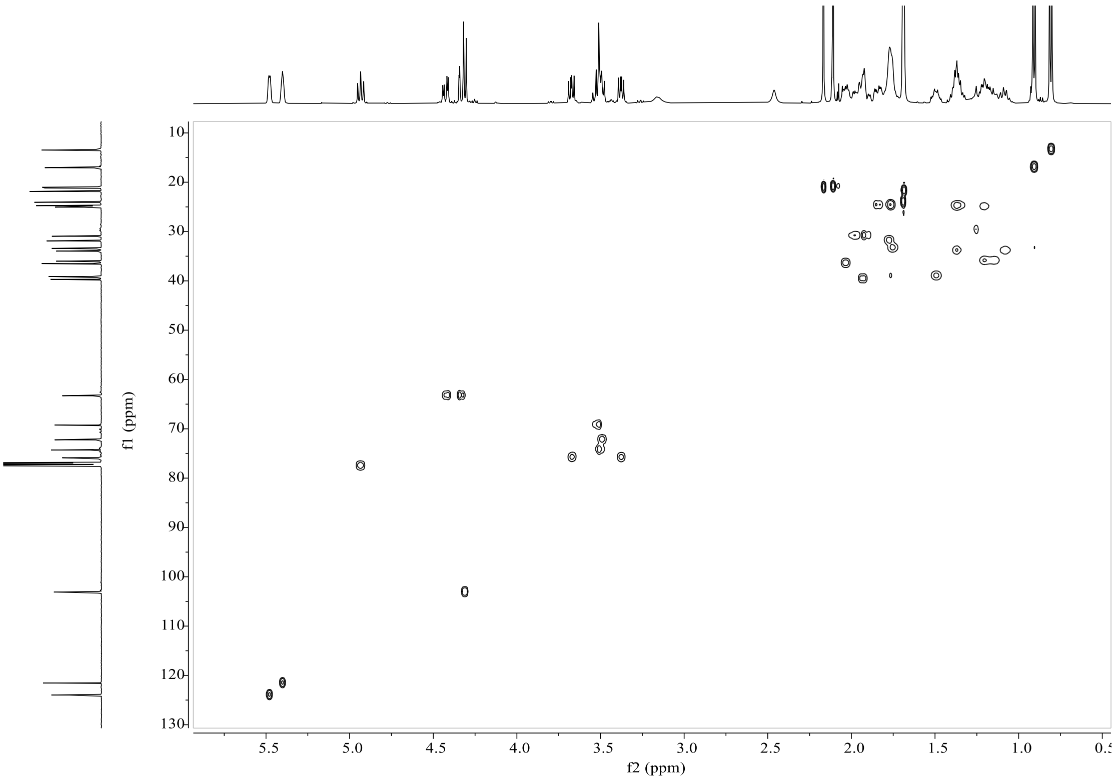

Figure S14 HSQC spectrum of Lemnabourside F (2) (600 MHz; $CDCl_3$)

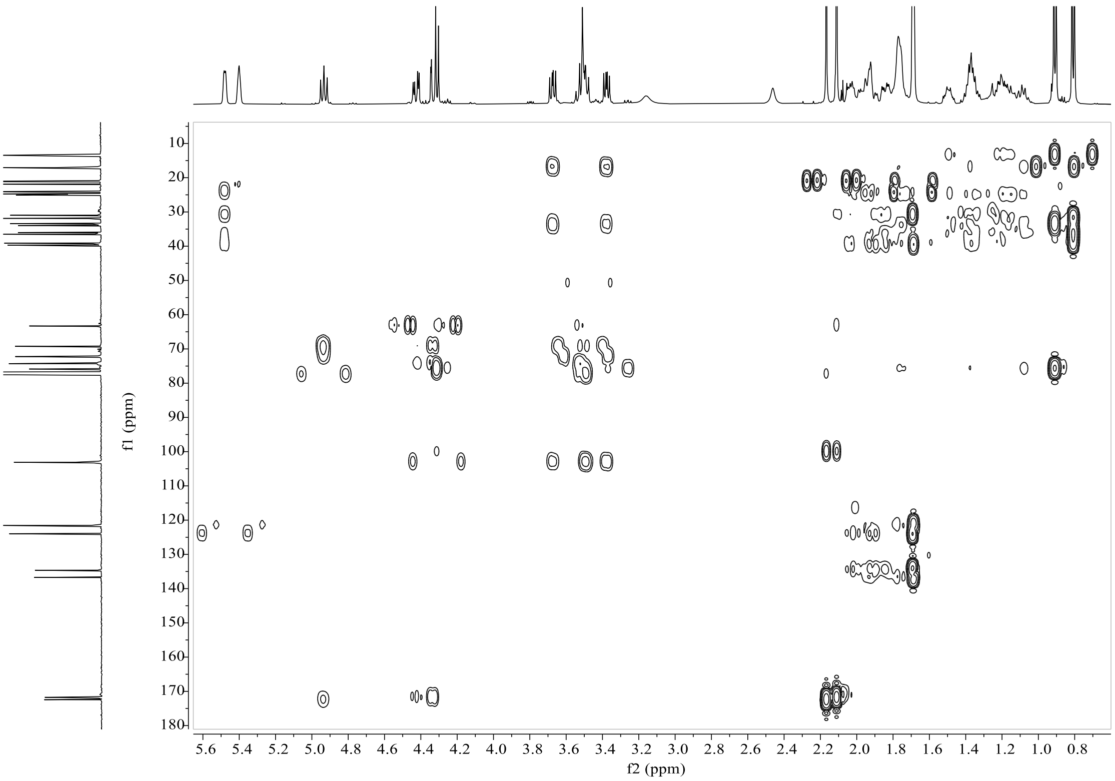

Figure S15 HMBC spectrum of Lemnabourside F (2) (600 MHz; $CDCl_3$)

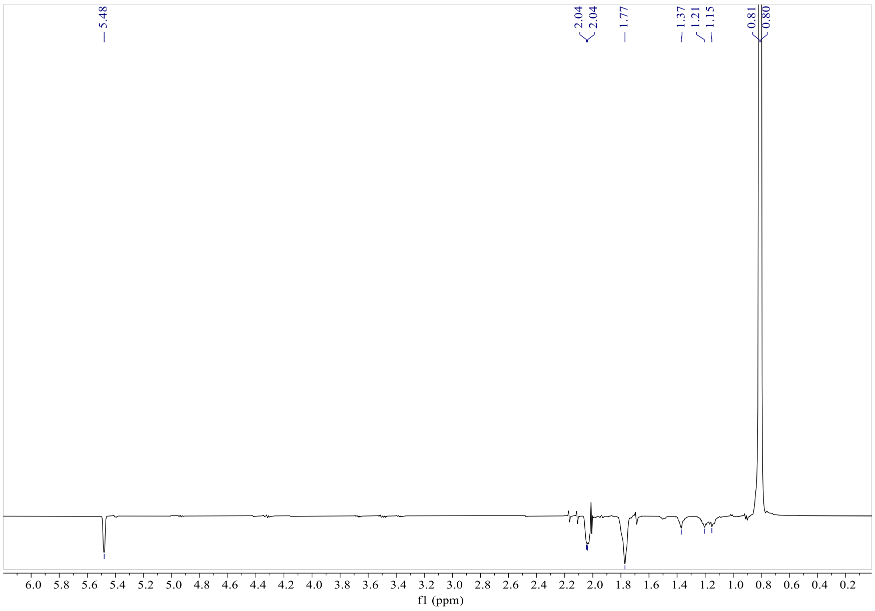

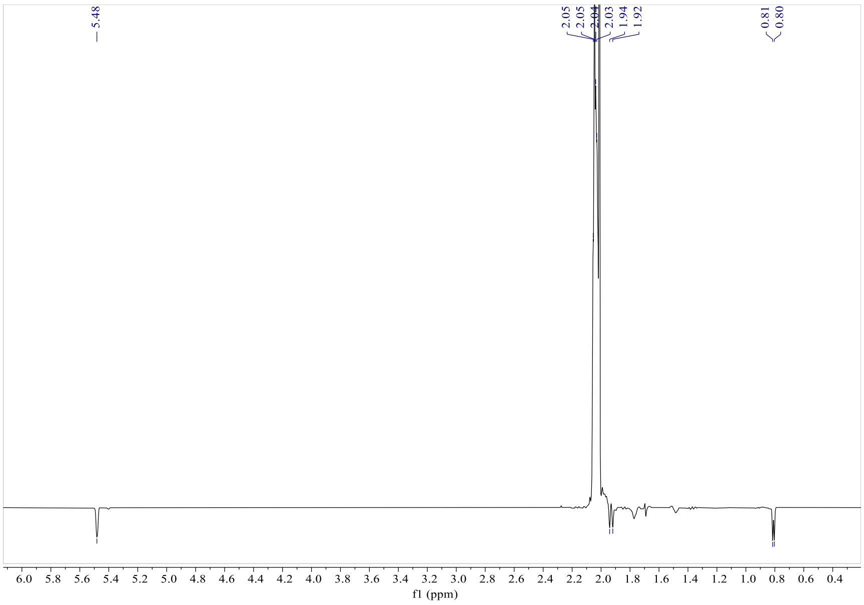

Figure S16 1D-NOE spectrum of Lemnabourside F (2) (600 MHz; $CDCl_3$)

3.3 Spectroscopic data for Lemnabourside G (3)

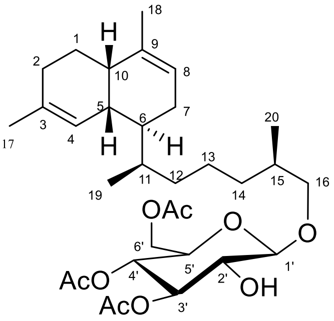

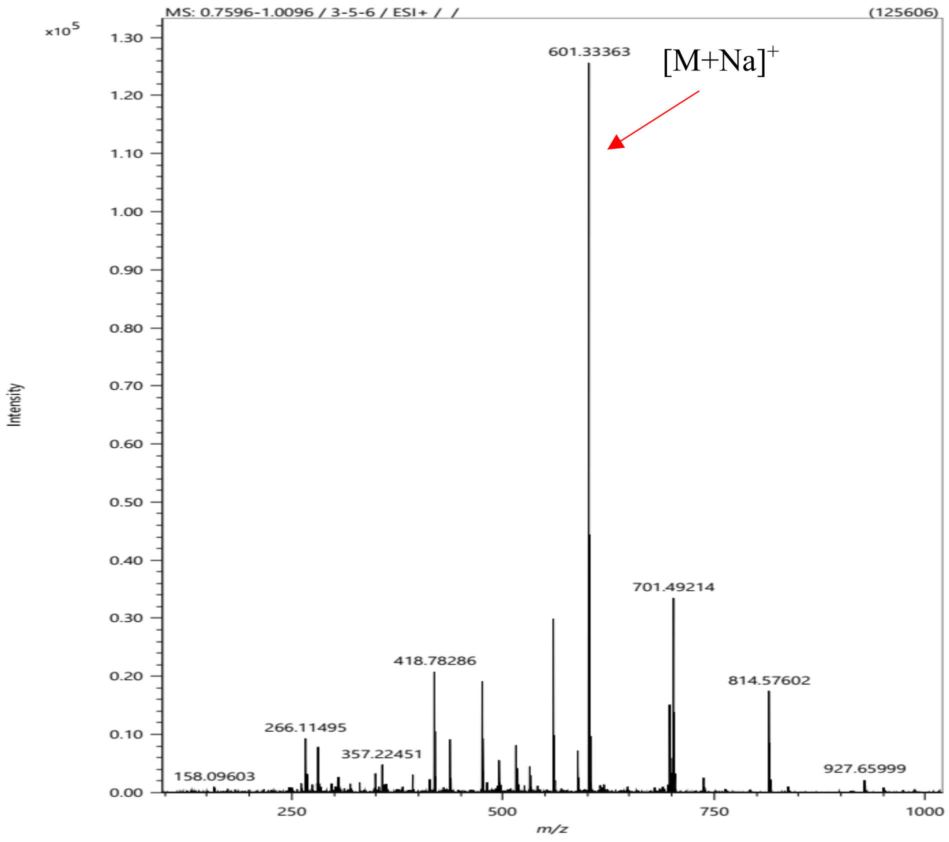

Figure S17 HRESIMS Spectrum of Lemnabourside G (3)

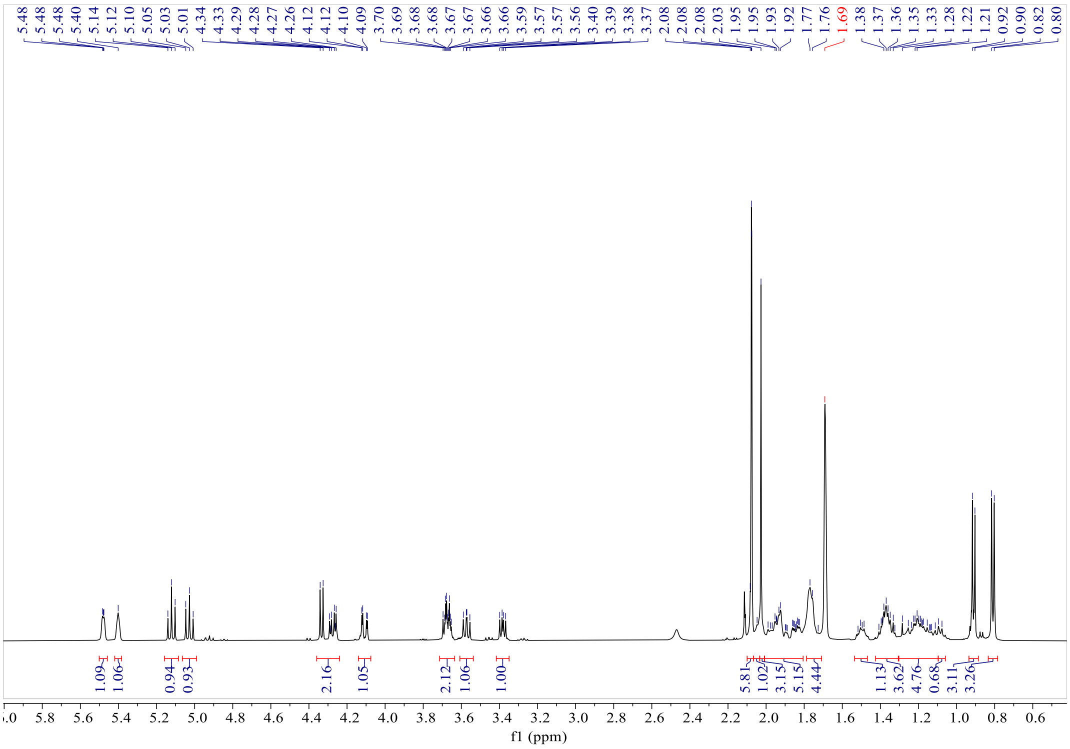

Figure S18 ^{1}H NMR Spectrum of Lemnabourside G (3) (600 MHz; $CDCl_3$)

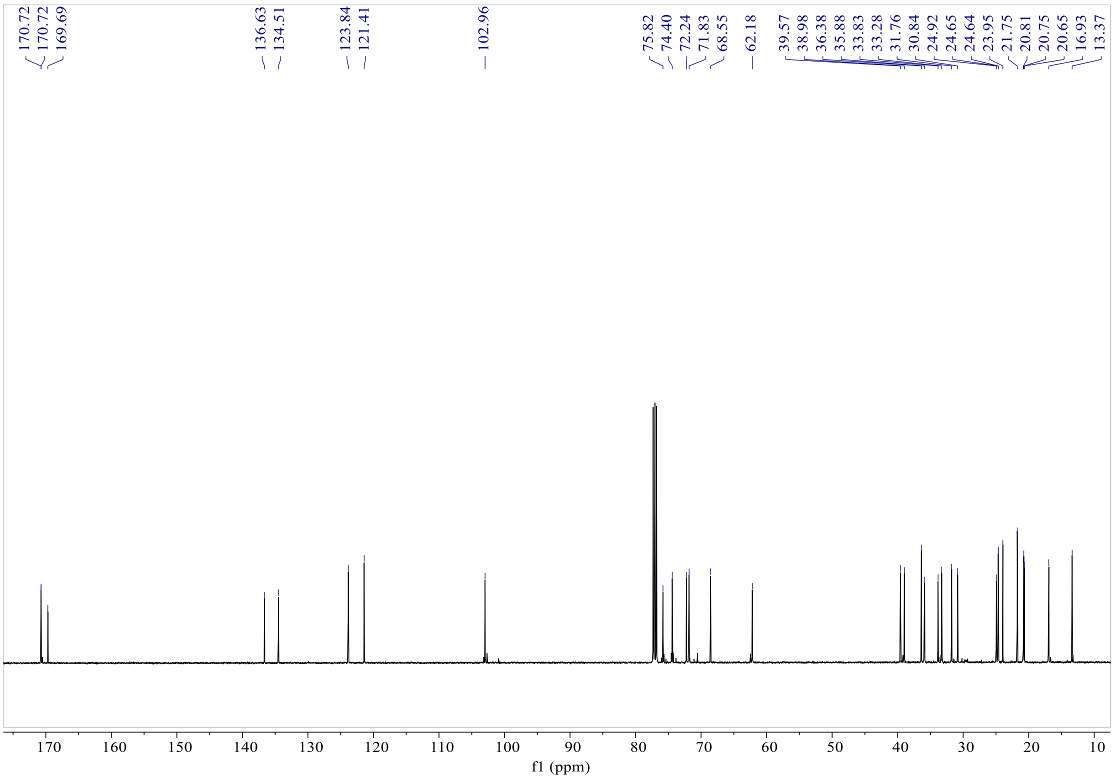

Figure S19 ^{13}C NMR Spectrum of Lemnabourside G (3) (150 MHz; $CDCl_3$)

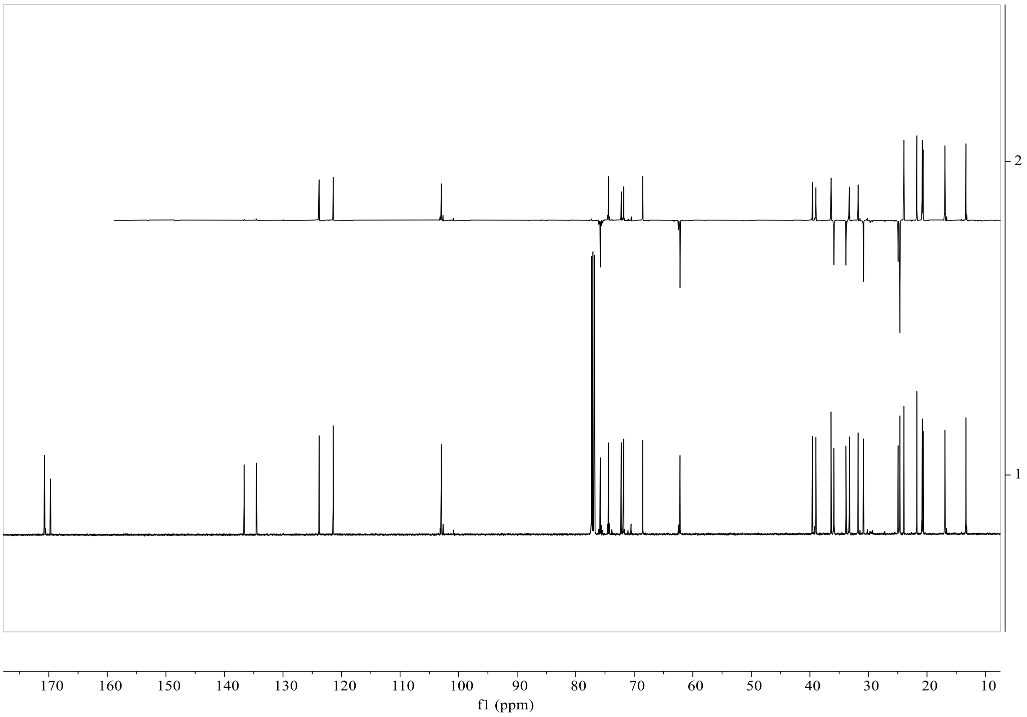

Figure S20 Dept 135° NMR Spectrum of Lemnabourside G (3) (150 MHz; $CDCl_3$)

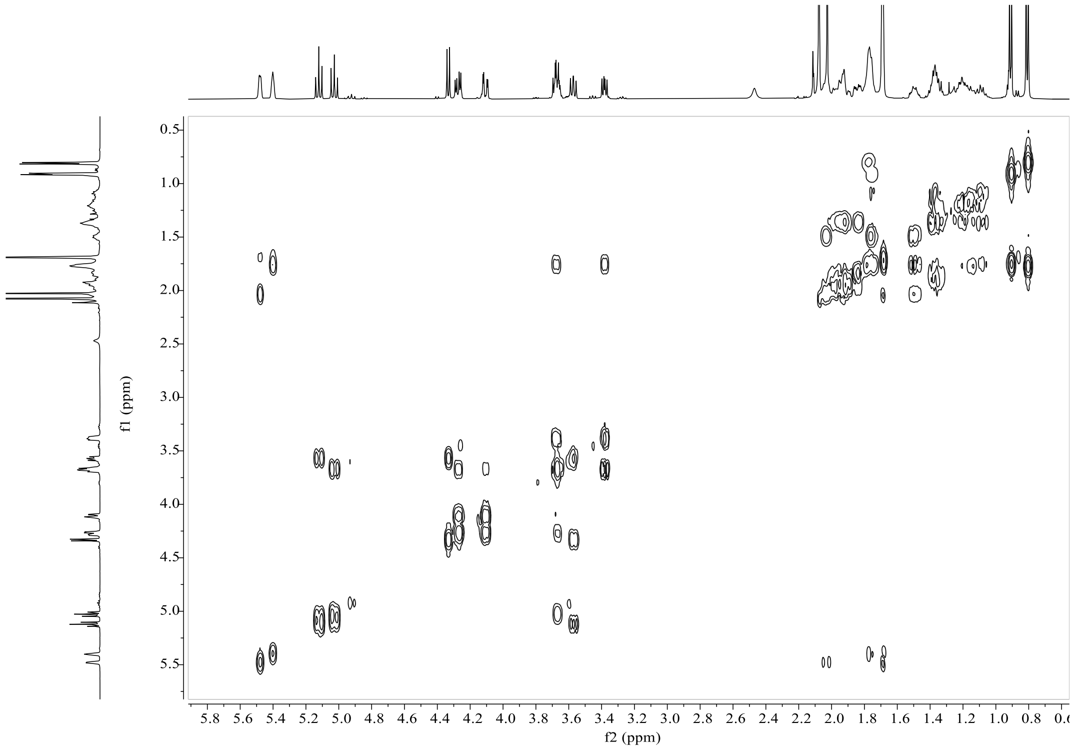

Figure S21 ^{1}H-^{1}H COSY spectrum of Lemnabourside G (3) (600 MHz; $CDCl_3$)

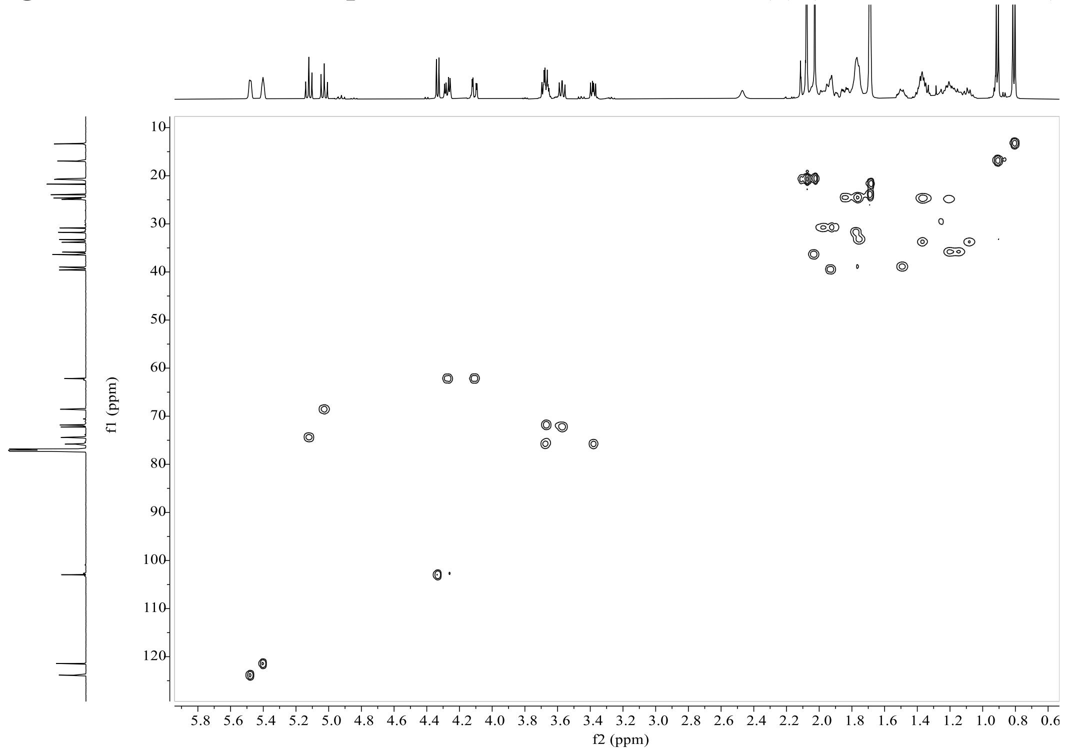

Figure S22 HSQC spectrum of Lemnabourside G (3) (600 MHz; $CDCl_3$)

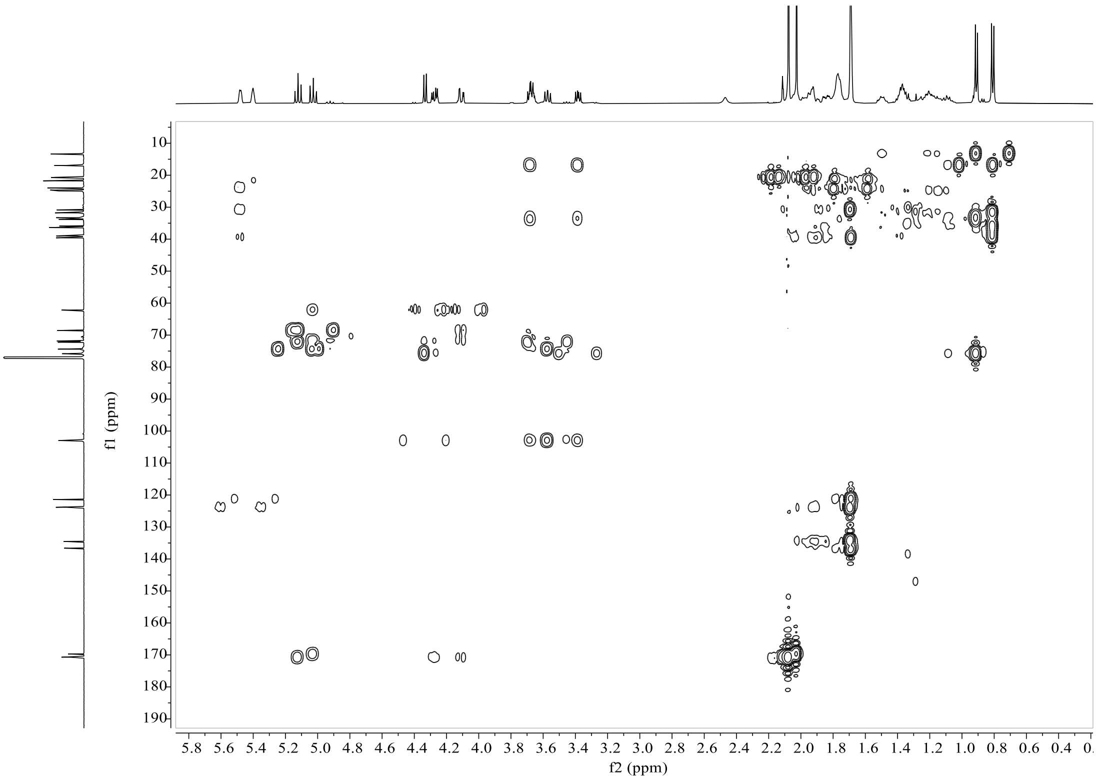

Figure S23 HMBC spectrum of Lemnabourside G (3) (600 MHz; CDCl$_3$)

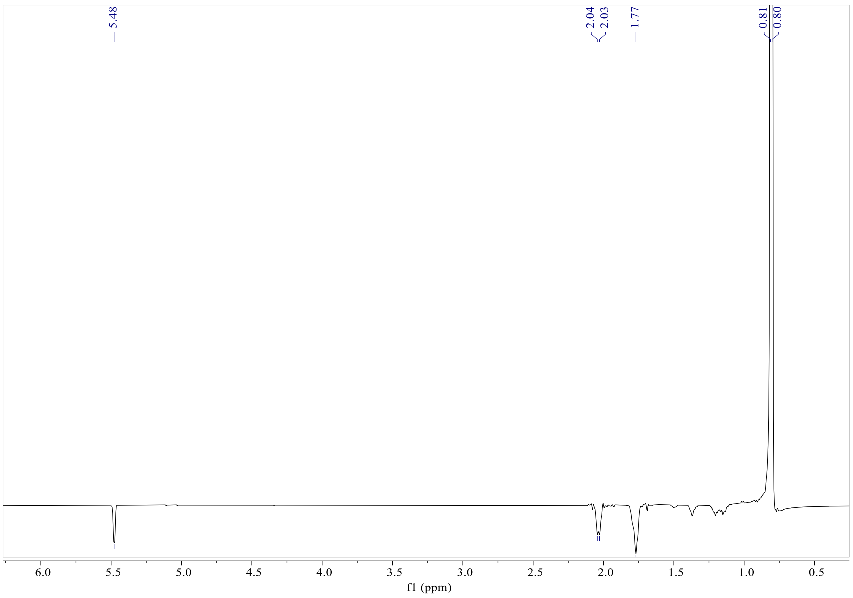

Figure S24 1D-NOE spectrum of Lemnabourside G (3) (600 MHz; CDCl$_3$)

3.4 Spectroscopic data for Lemnadiolbourside H (4)

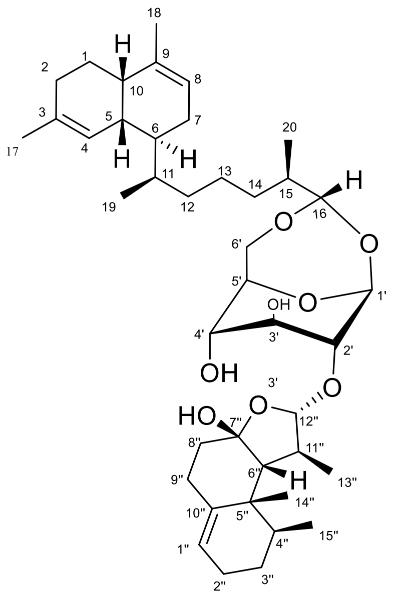

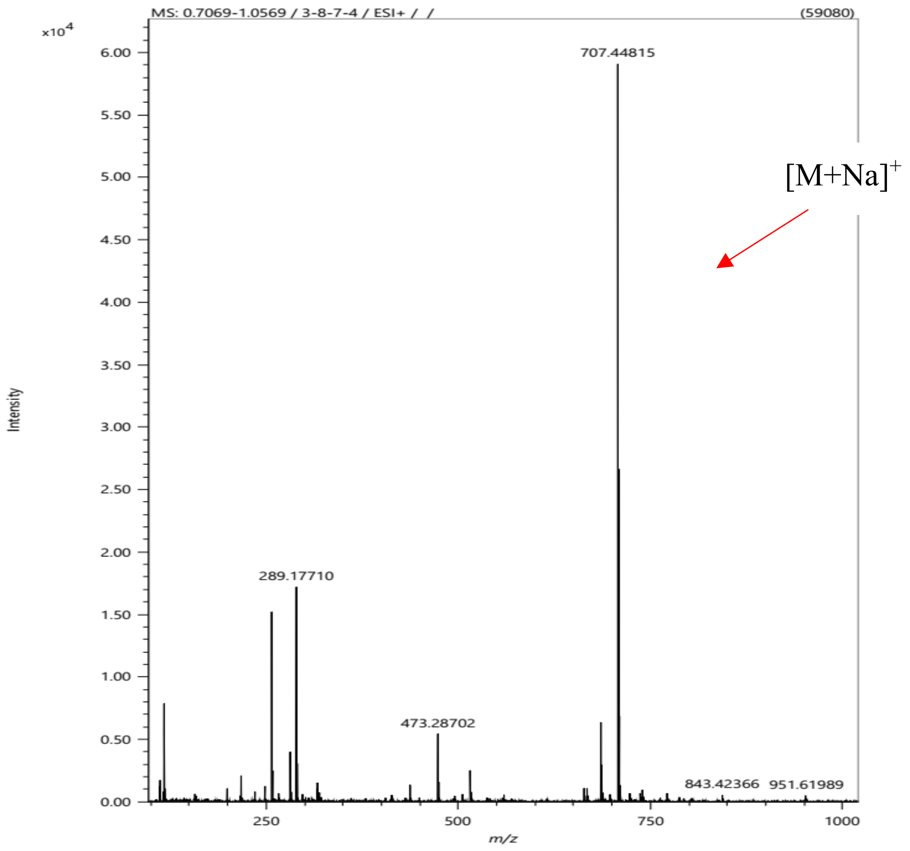

Figure S25 HRESIMS Spectrum of Lemnadiolbourside A (4)

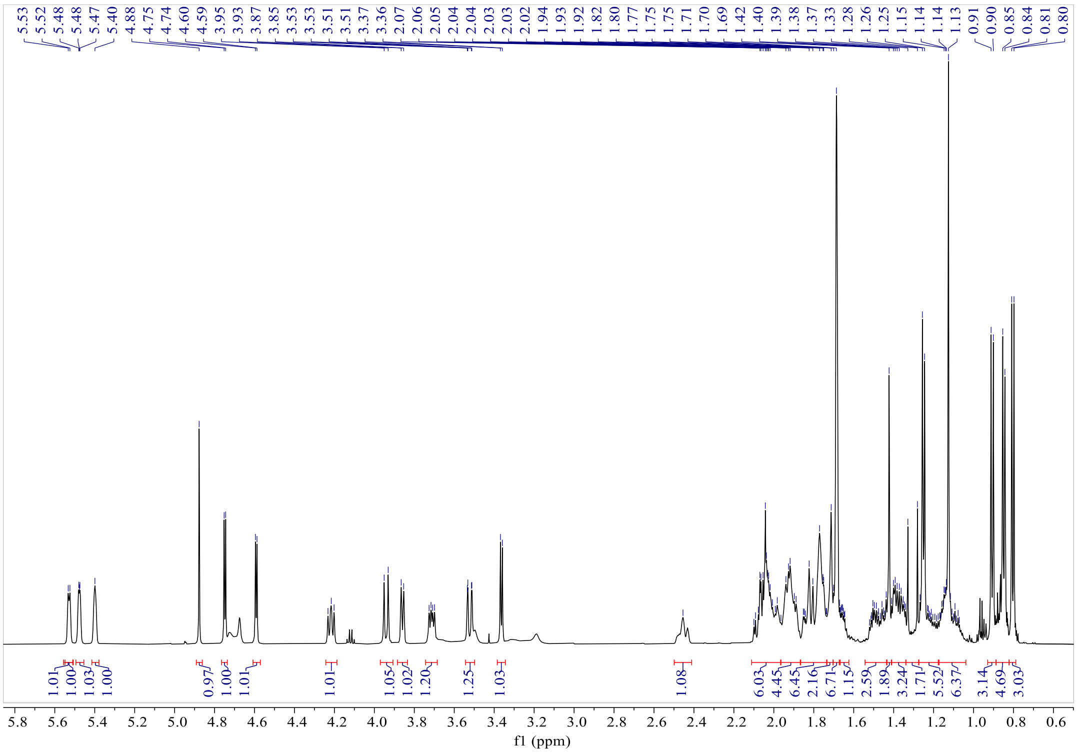

Figure S26 ^{1}H NMR Spectrum of Lemnadiolbourside A (4) (600 MHz; $CDCl_3$)

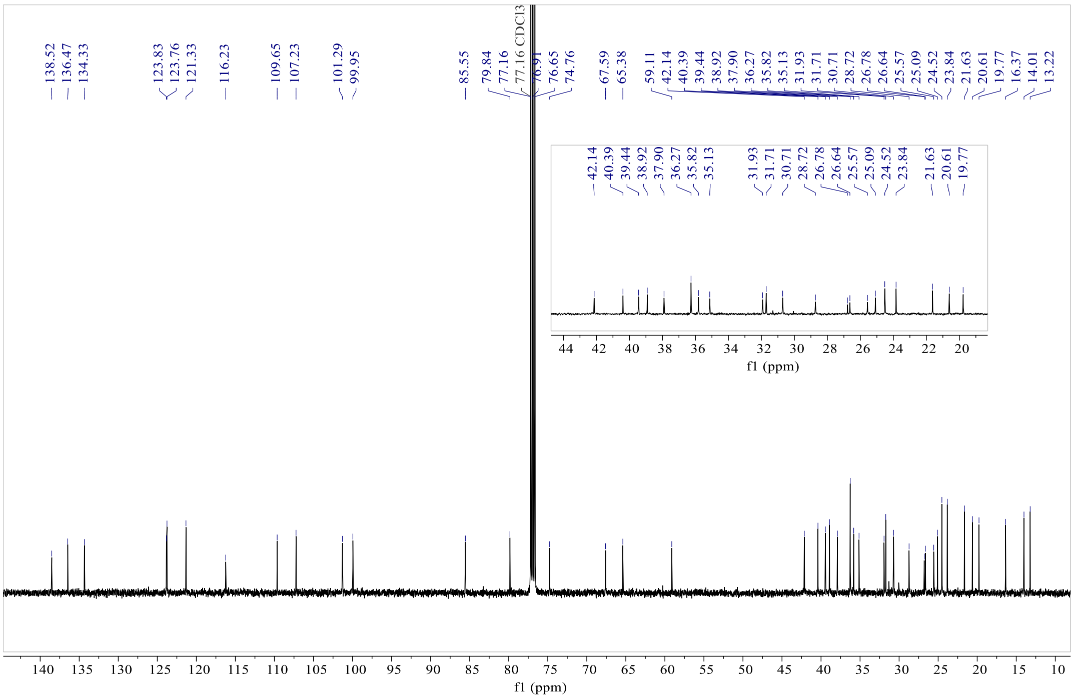

Figure S27 ^{13}C NMR Spectrum of Lemnadiolbourside A (4) (150 MHz; $CDCl_3$)

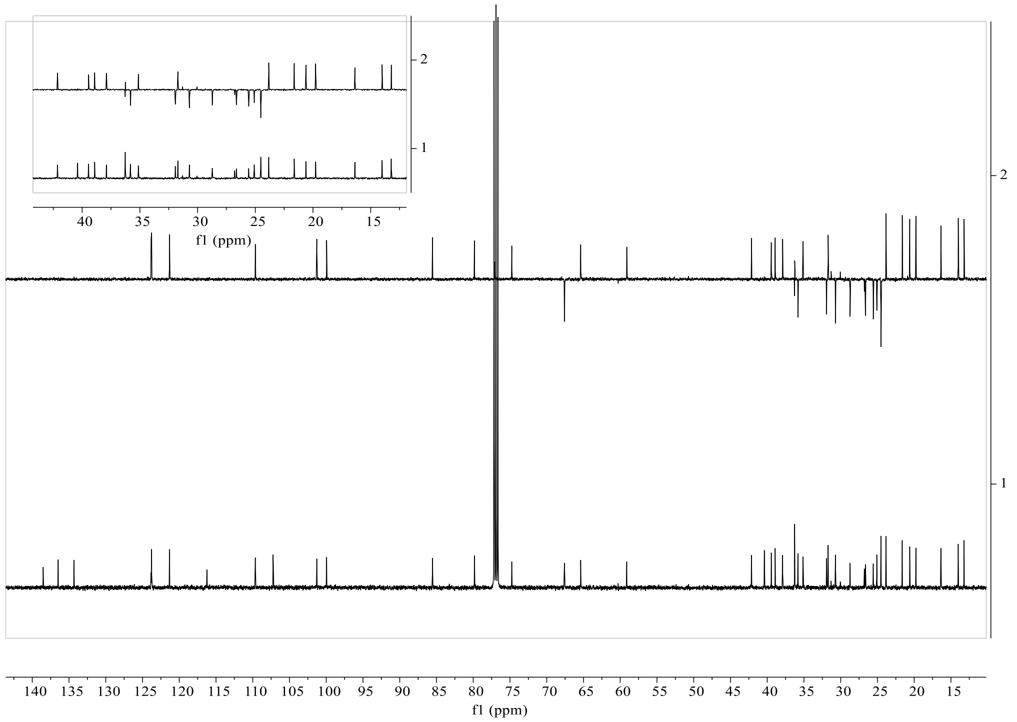

Figure S28 Dept 135° NMR Spectrum of Lemnadiolbourside A (4) (150 MHz; $CDCl_3$)

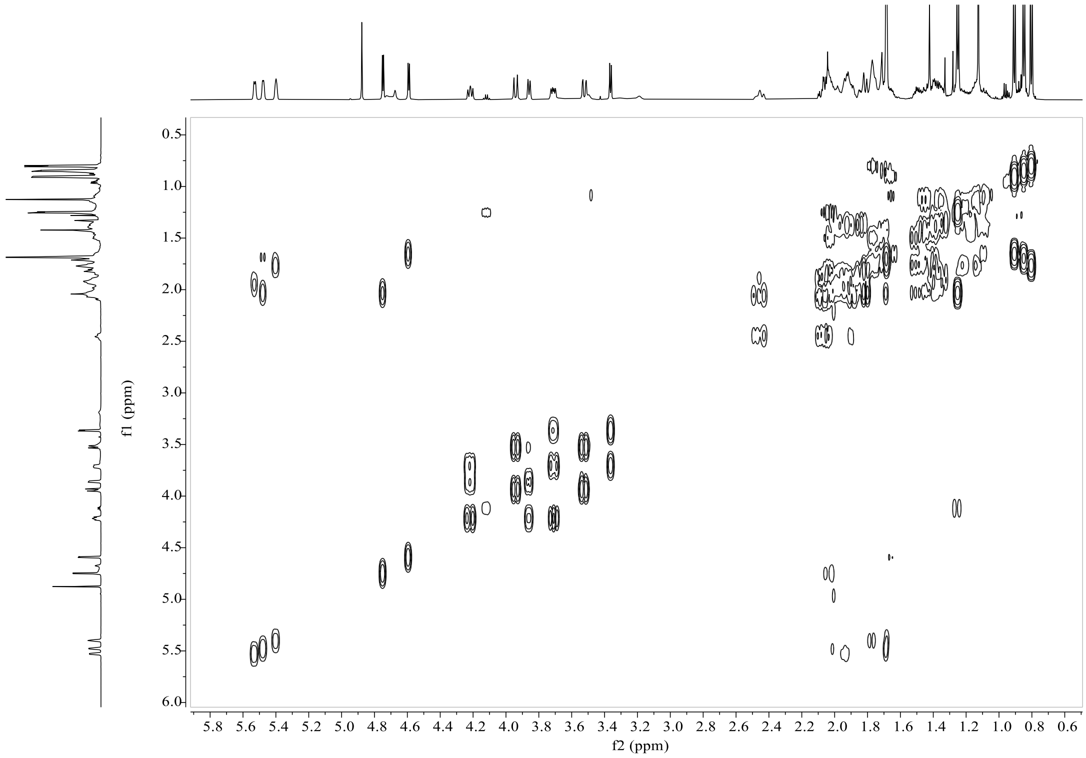

Figure S29 ^1H-^1H COSY spectrum of Lemnadiolbourside A (4) (600 MHz; $CDCl_3$)

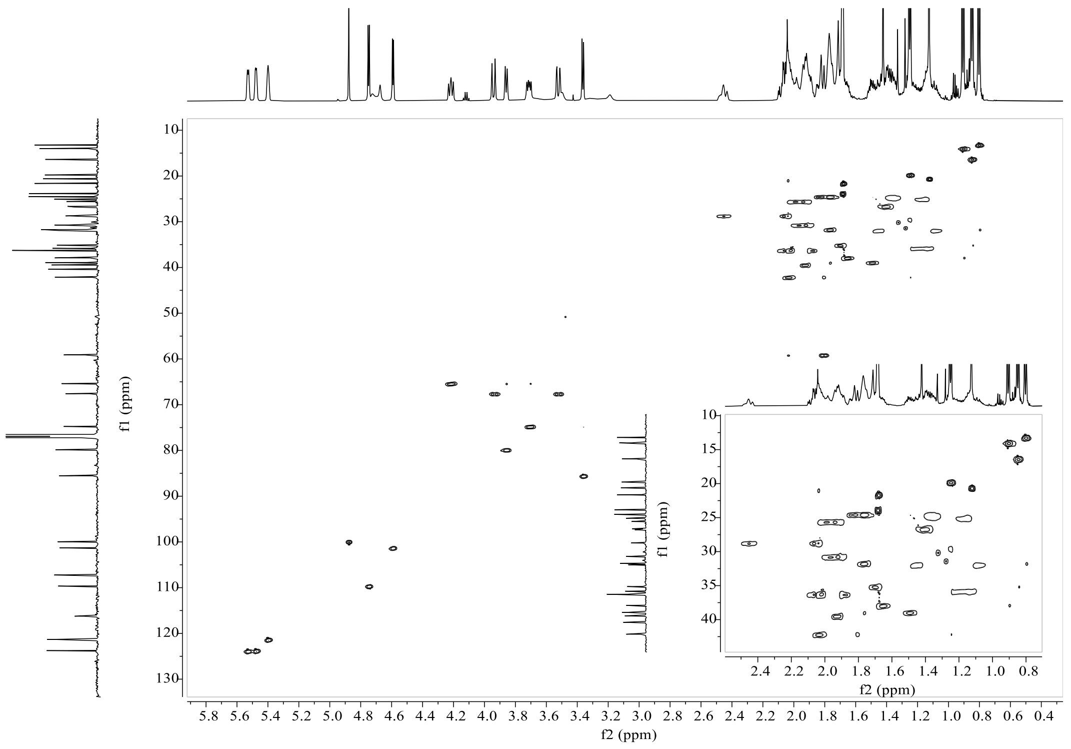

Figure S30 HSQC spectrum of Lemnadiolbourside A (4) (600 MHz; $CDCl_3$)

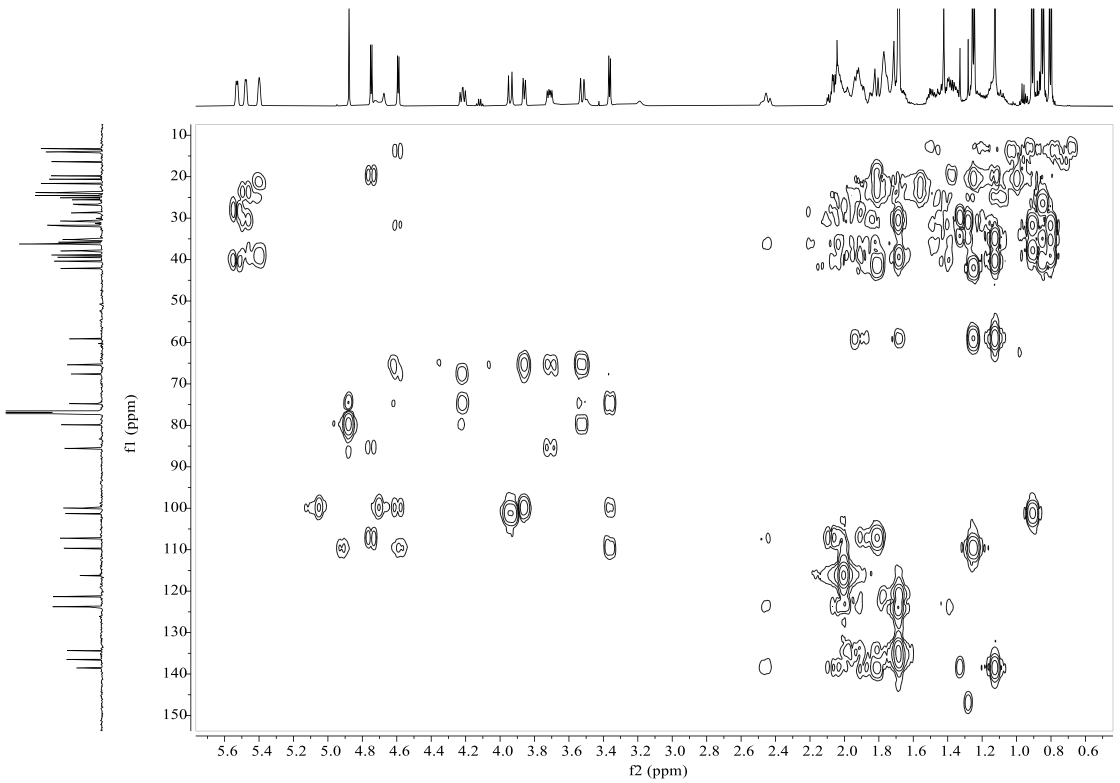

Figure S31 HMBC spectrum of Lemnadiolbourside A (4) (600 MHz; $CDCl_3$)

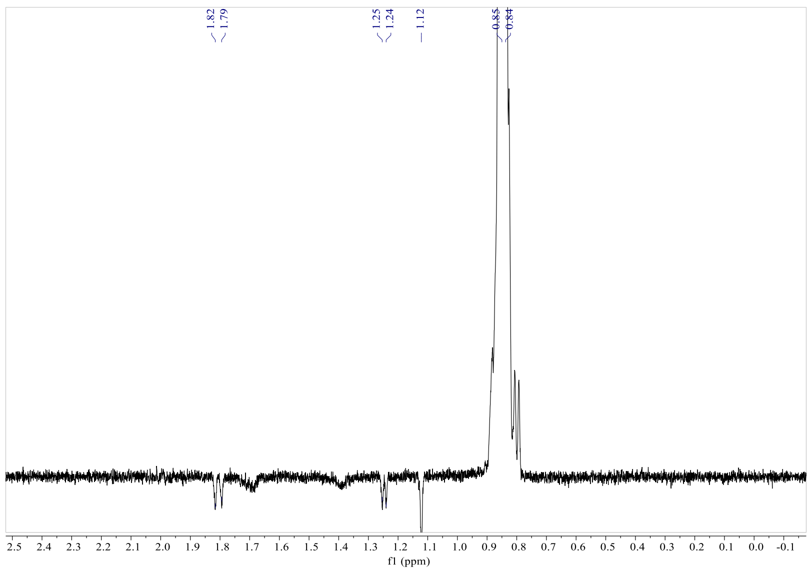
1.82
1.79
1.25
1.24
1.12
0.85
0.84
2.5 2.4 2.3 2.2 2.1 2.0 1.9 1.8 1.7 1.6 1.5 1.4 1.3 1.2 1.1 1.0 0.9 0.8 0.7 0.6 0.5 0.4 0.3 0.2 0.1 0.0 -0.1
f1 (ppm)

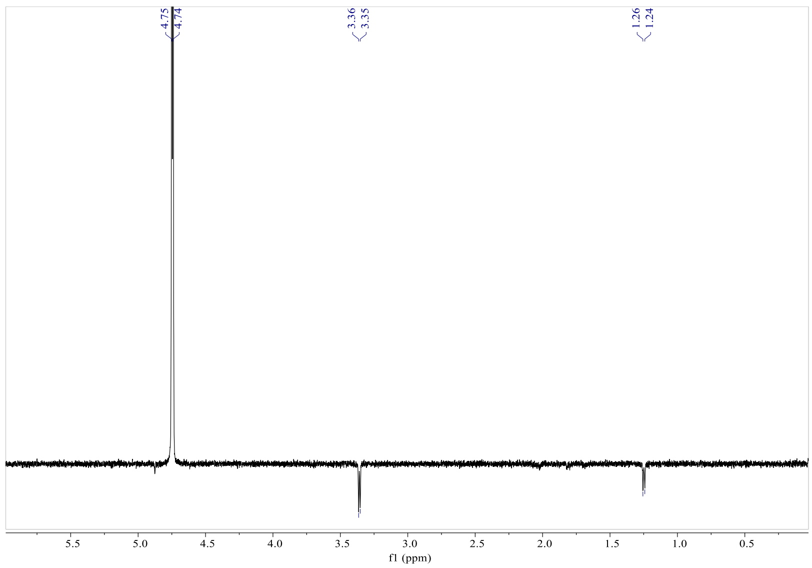
4.75
4.74
3.36
3.35
1.26
1.24
5.5 5.0 4.5 4.0 3.5 3.0 2.5 2.0 1.5 1.0 0.5
f1 (ppm)

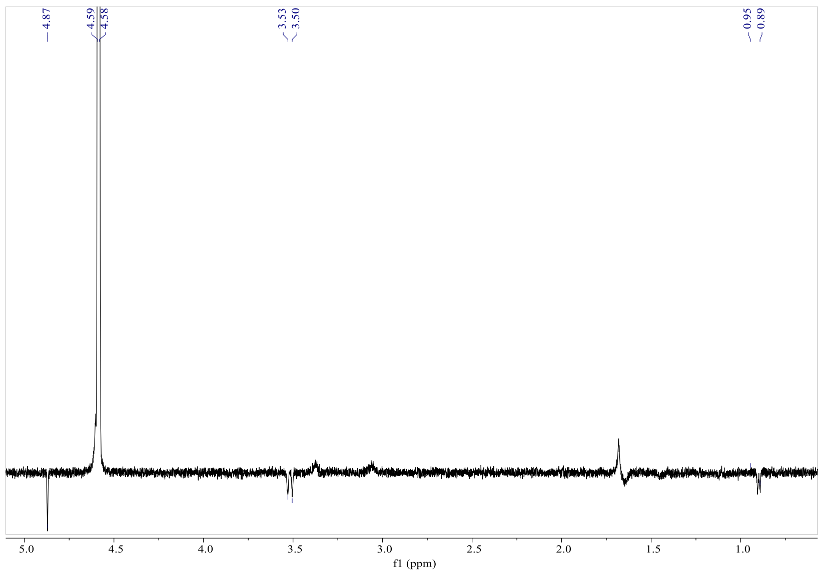
4.87
4.59
4.58
3.53
3.50
0.95
0.89
5.0 4.5 4.0 3.5 3.0 2.5 2.0 1.5 1.0
f1 (ppm)

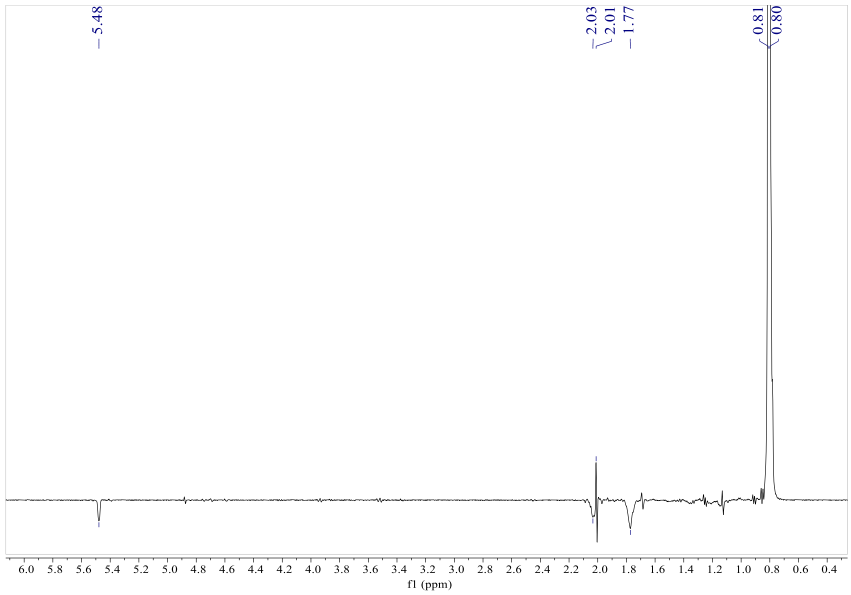

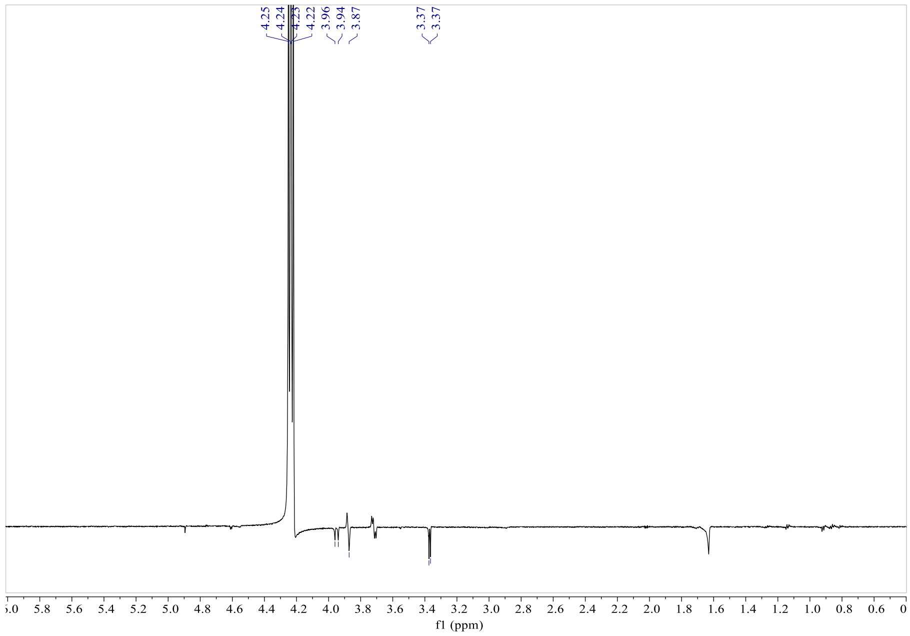

Figure S32 1D-NOE spectrum of Lemnadiolbourside A (4) (600 MHz; $CDCl_3$)

3.5 Spectroscopic data for Lemnadiolbourside B (5)

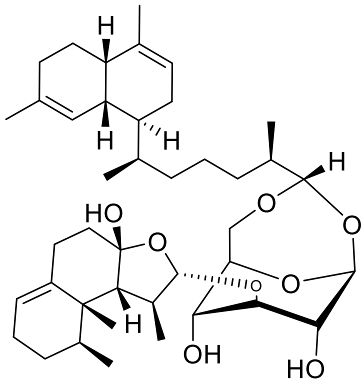

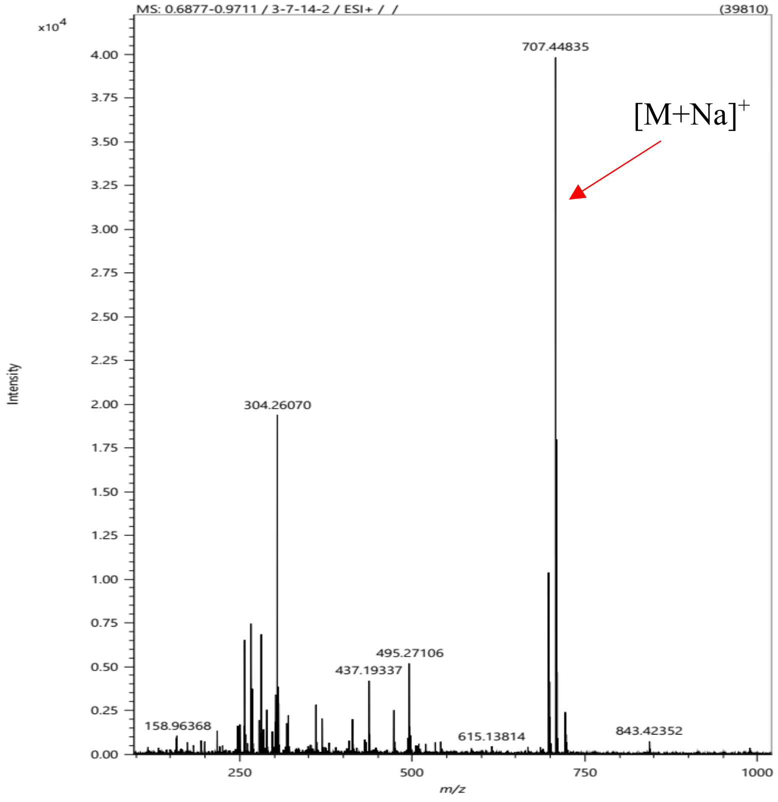

Figure S33 HRESIMS Spectrum of Lemnadiolbourside B (5)

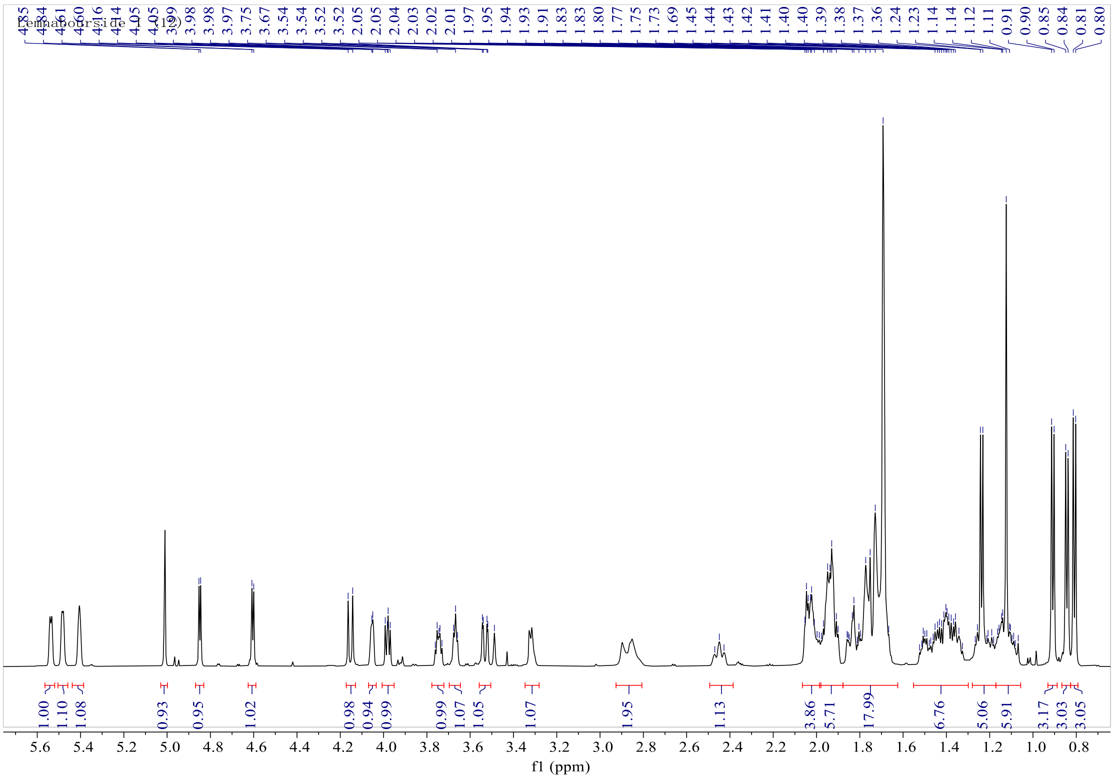

Figure S34 ^{1}H NMR Spectrum of Lemnadiolbourside B (5) (600 MHz; $CDCl_3$)

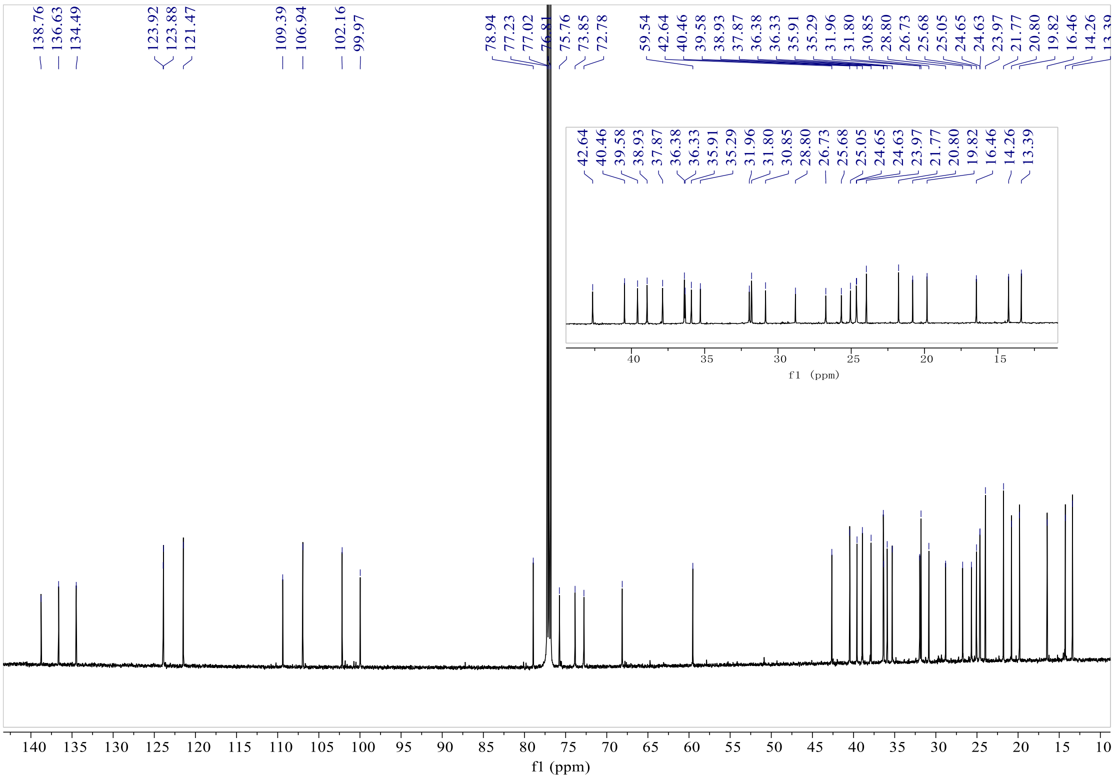

Figure S35 ^{13}C NMR Spectrum of Lemnadiolbourside B (5) (150 MHz; $CDCl_3$)

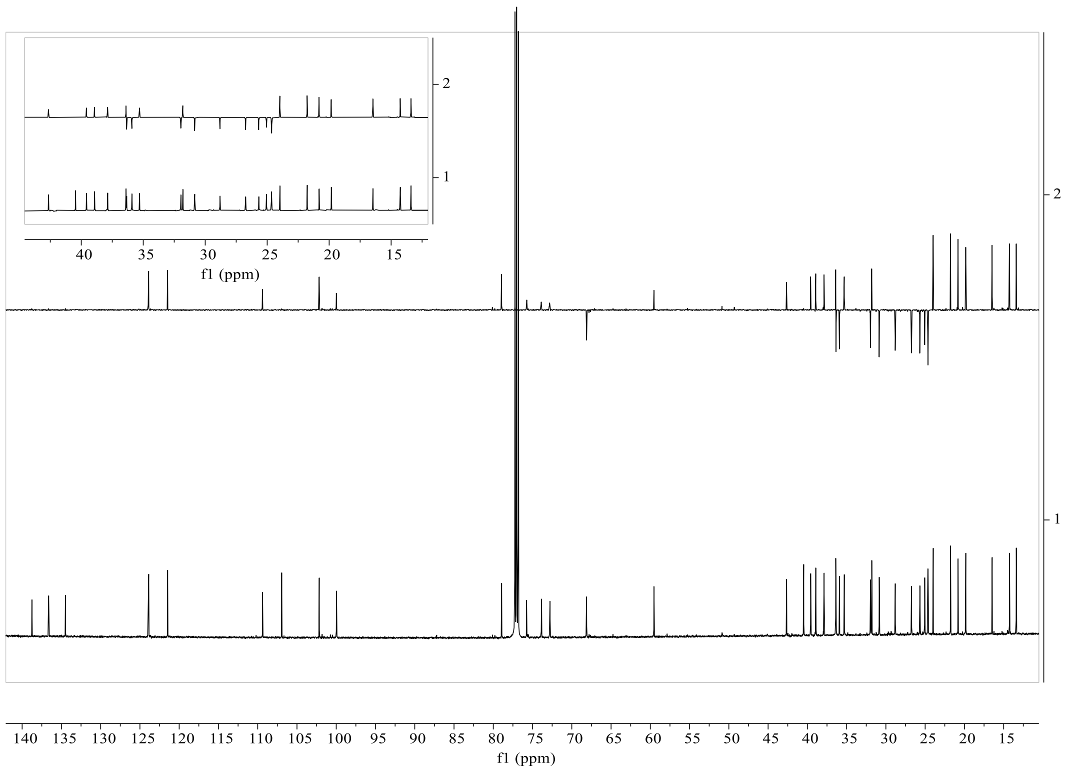

Figure S36 Dept 135° NMR Spectrum of Lemnadiolbourside B (5) (150 MHz; $CDCl_3$)

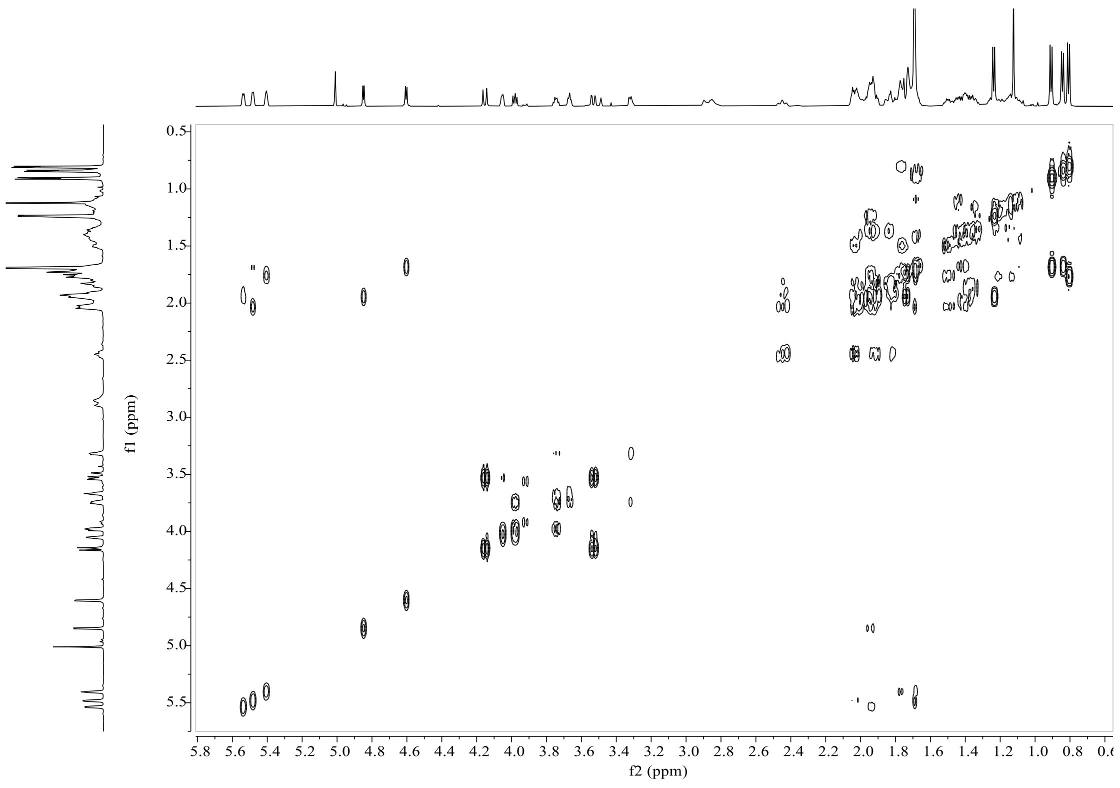

Figure S37 ^1H-^1H COSY spectrum of Lemnadiolbourside B (5) (600 MHz; $CDCl_3$)

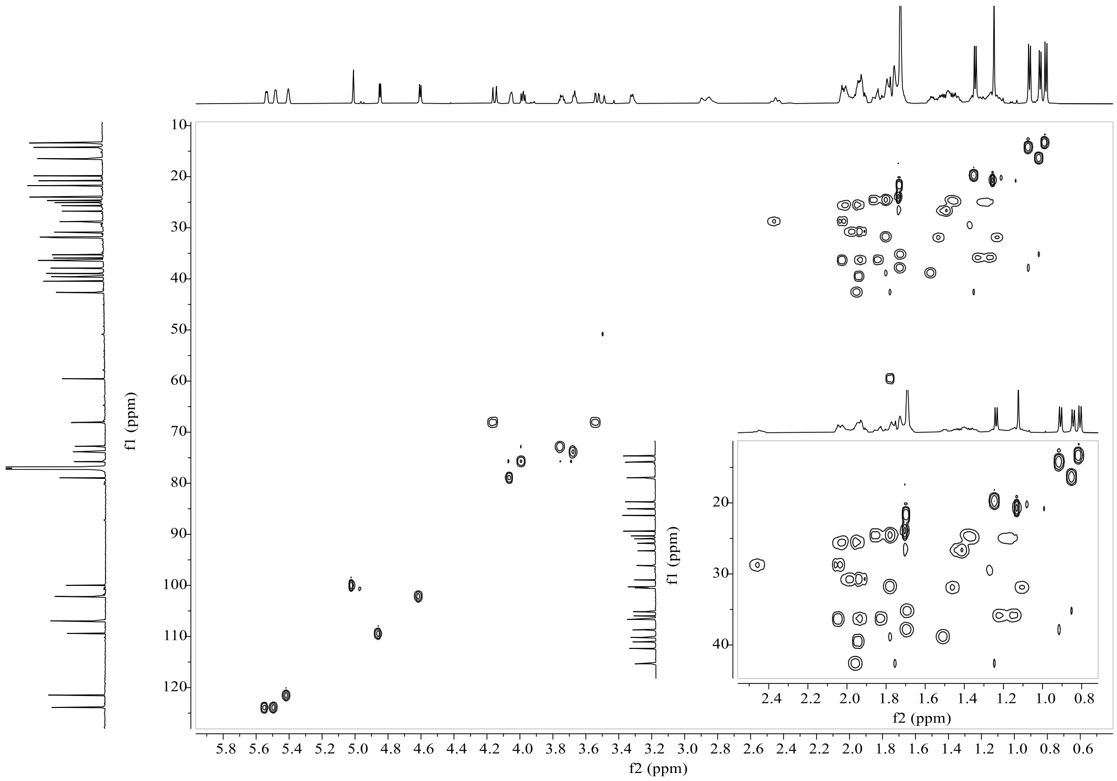

Figure S38 HSQC spectrum of Lemnadiolbourside B (5) (600 MHz; $CDCl_3$)

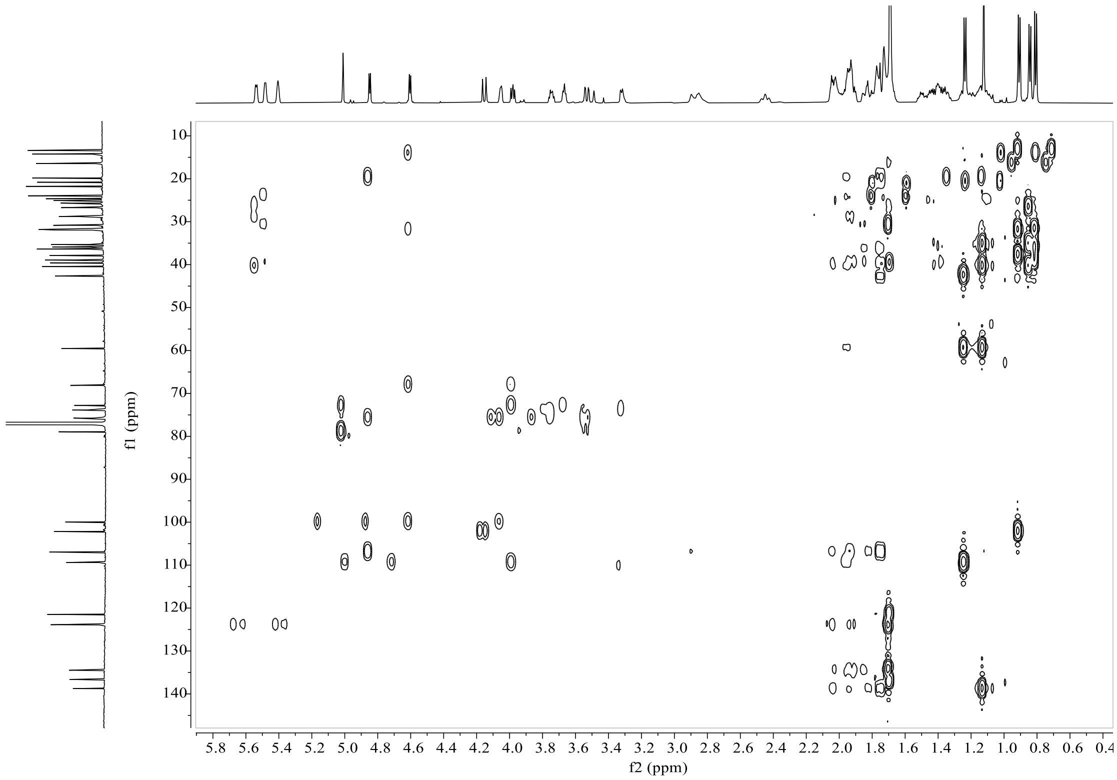

Figure S39 HMBC spectrum of Lemnadiolbourside B (5) (600 MHz; CDCl$_3$)

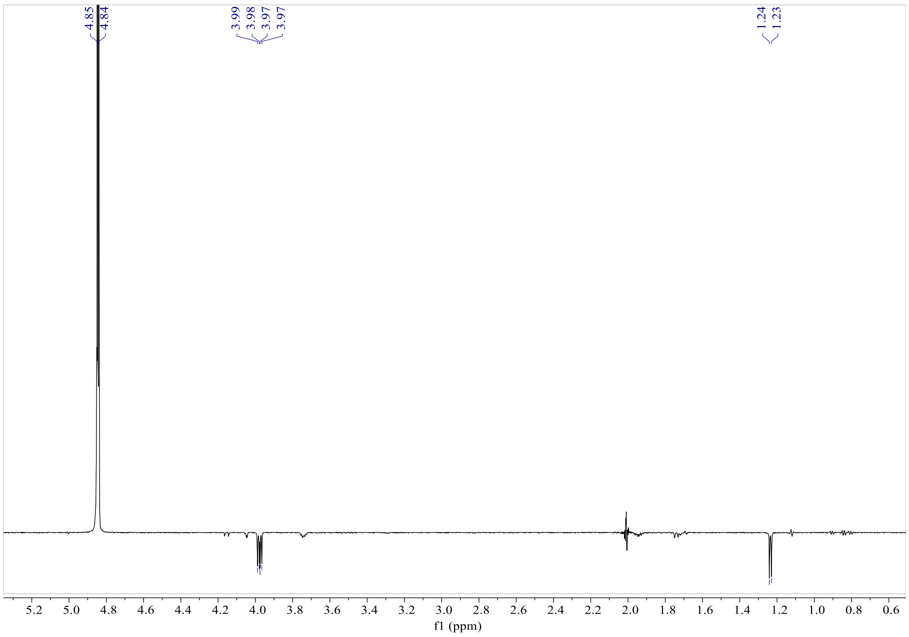

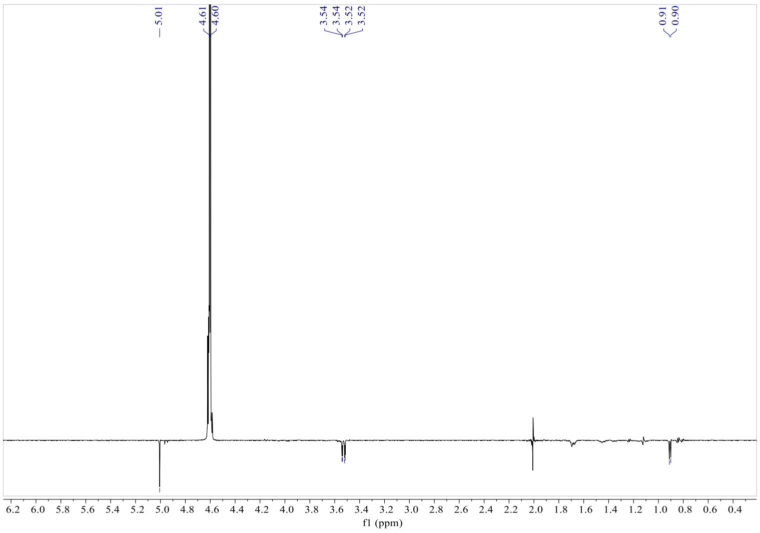

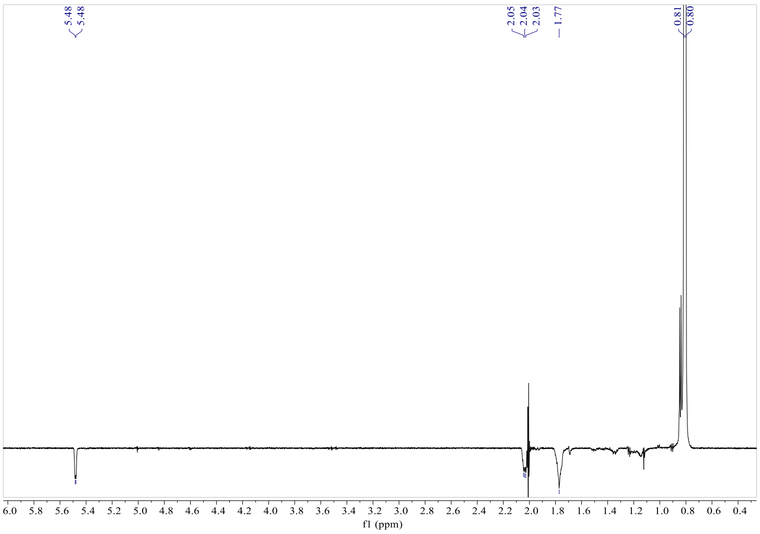

Figure S40 1D-NOE spectrum of Lemnadiolbourside B (5) (600 MHz; $CDCl_3$)

3.6 Spectroscopic data for Lemnadiolbourside C (6)

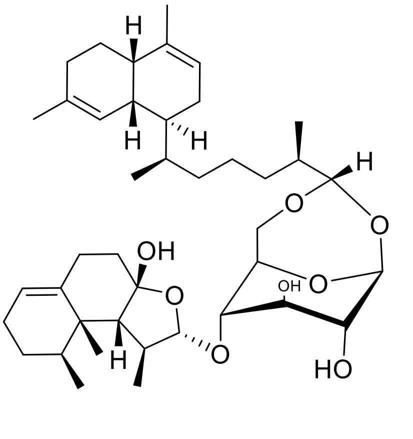

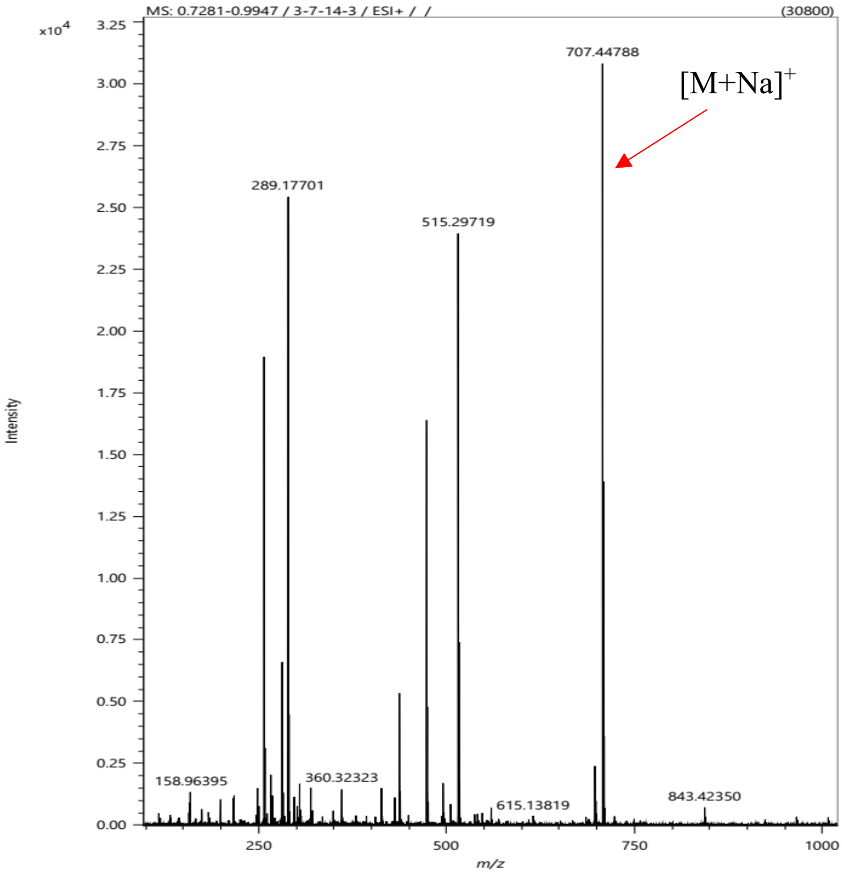

Figure S41 HRESIMS Spectrum of Lemnadiolbourside C (6)

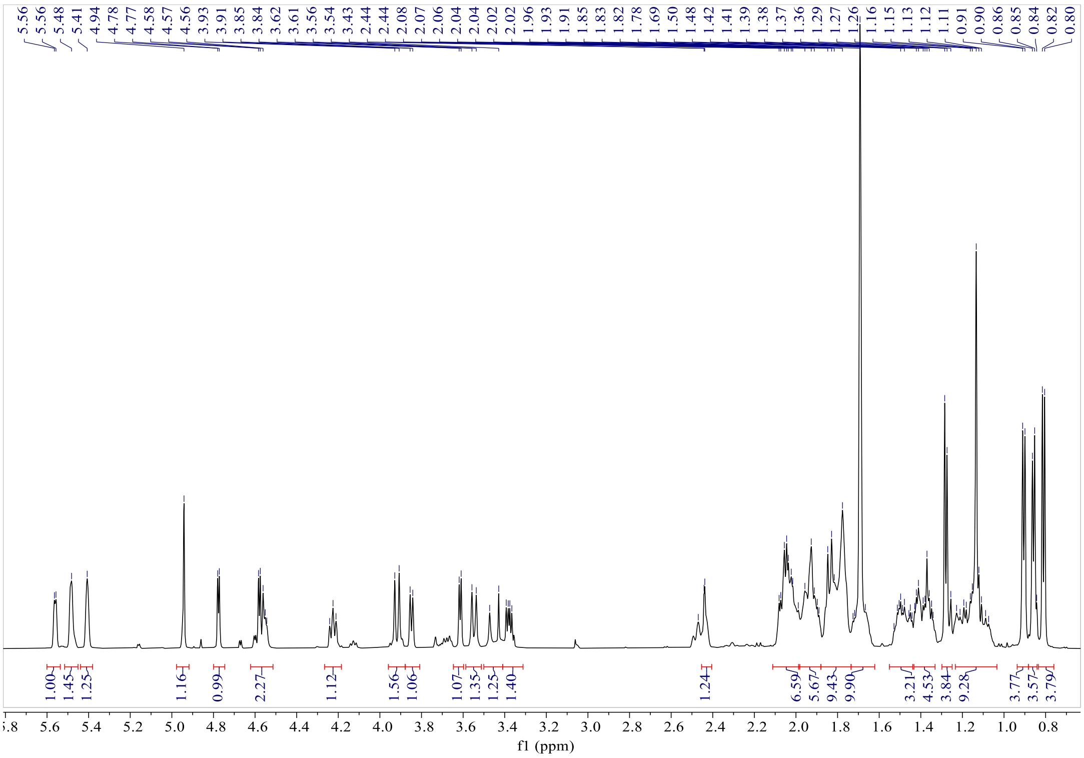

Figure S42 ^{1}H NMR Spectrum of Lemnadiolbourside C (6) (600 MHz; $CDCl_3$)

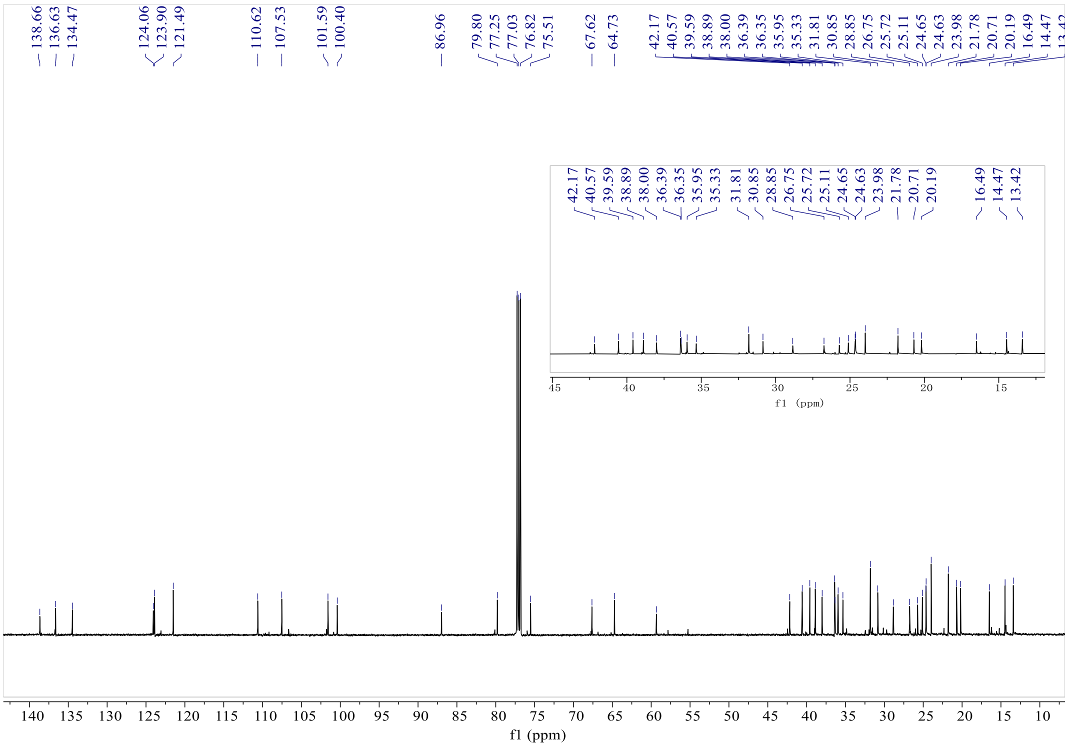

Figure S43 ^{13}C NMR Spectrum of Lemnadiolbourside C (6) (150 MHz; $CDCl_3$)

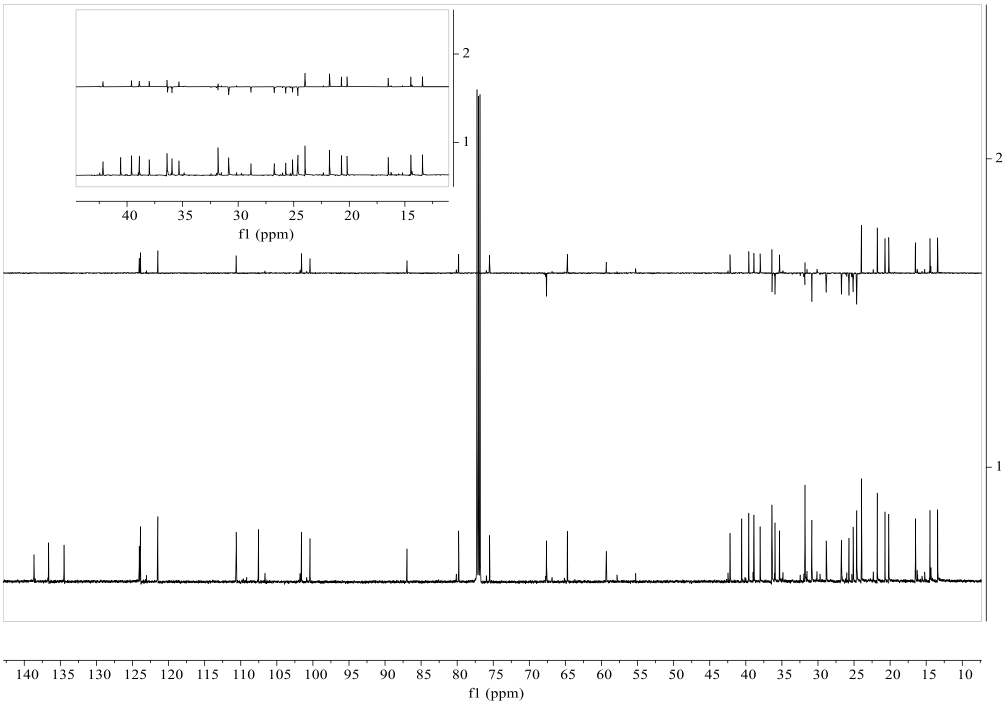

Figure S44 Dept 135° NMR Spectrum of Lemnadiolbourside C (6) (150 MHz; $CDCl_3$)

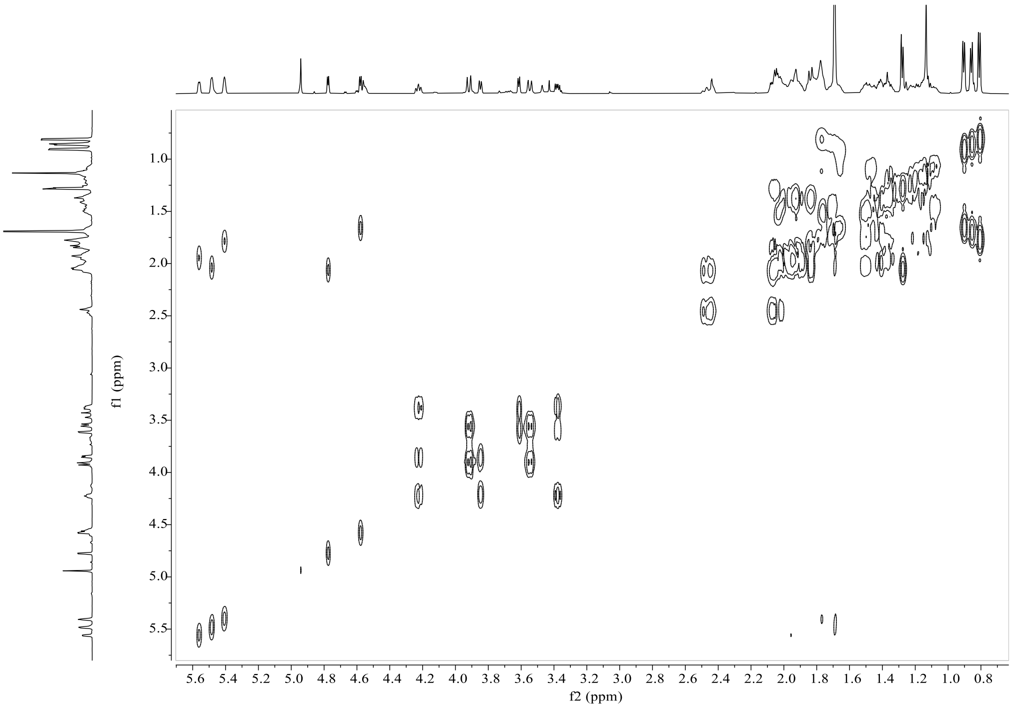

Figure S45 ^{1}H-^{1}H COSY spectrum of Lemnadiolbourside C (6) (600 MHz; $CDCl_3$)

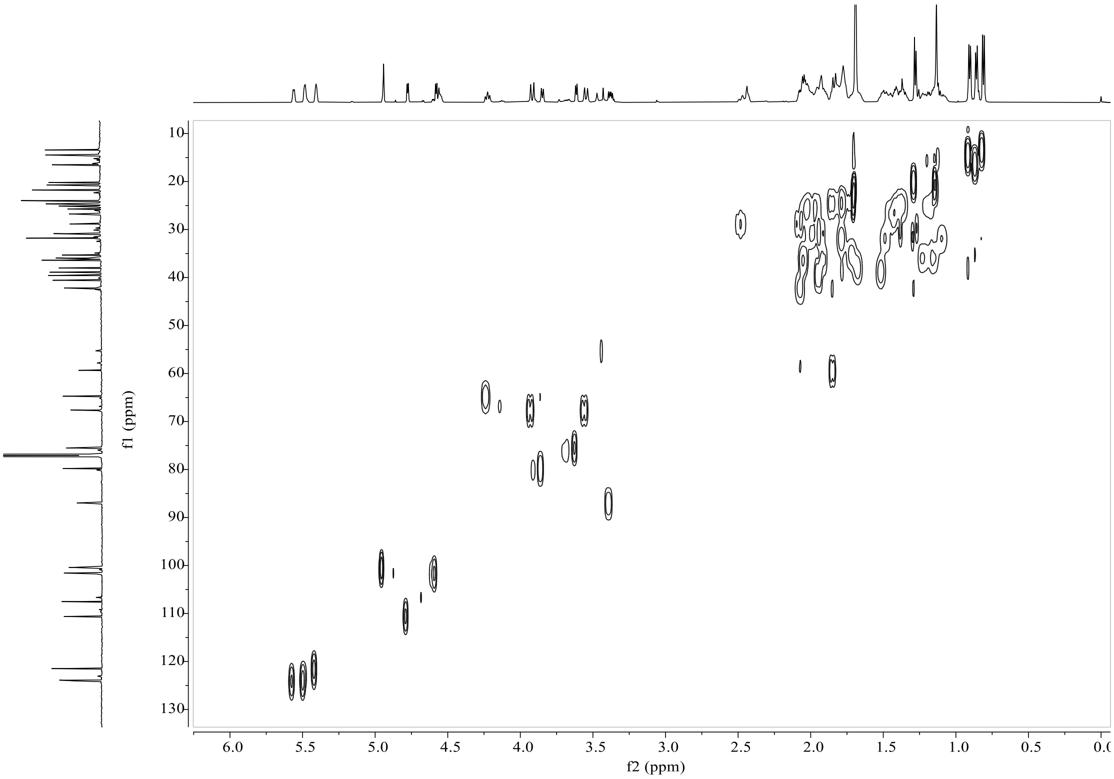

Figure S46 HSQC spectrum of Lemnadiolbourside C (6) (600 MHz; $CDCl_3$)

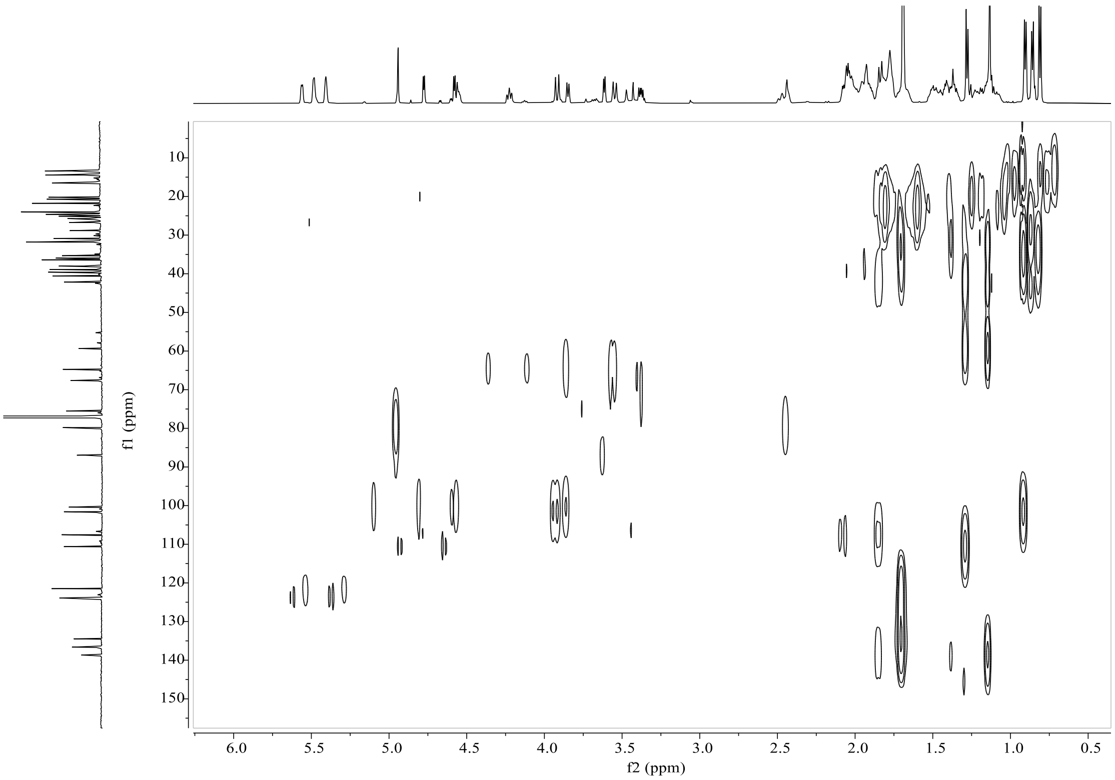

Figure S47 HMBC spectrum of Lemnadiolbourside C (6) (600 MHz; CDCl$_3$)

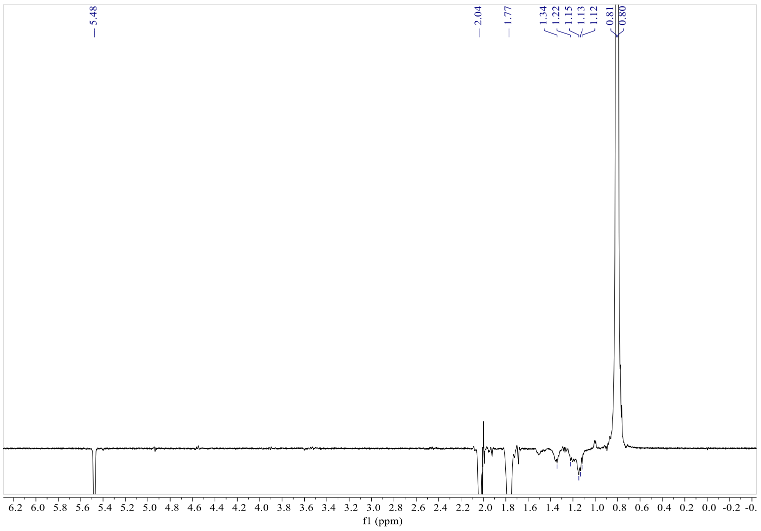

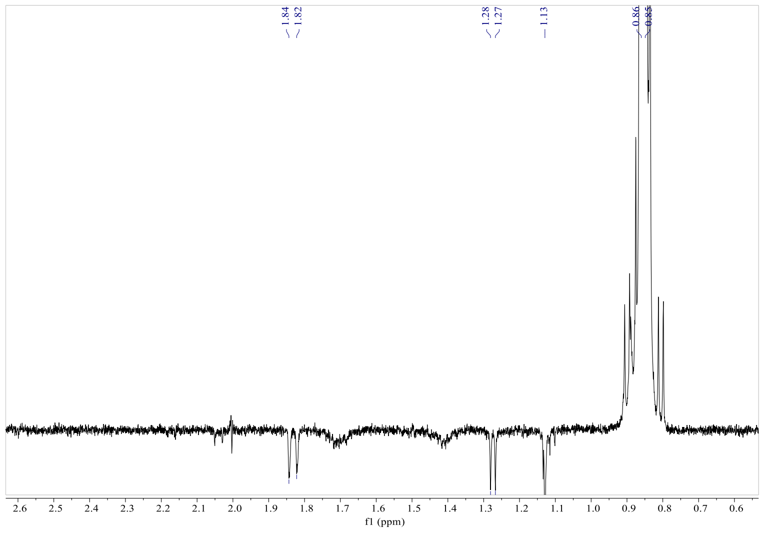
1.84
1.82
1.28
1.27
1.13
0.86
0.85
f1 (ppm)

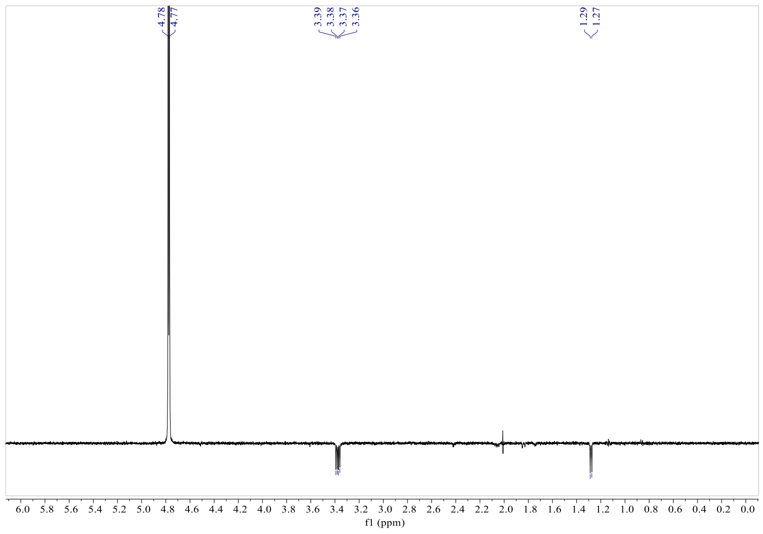
4.78
4.77
3.39
3.38
3.37
3.36
1.29
1.27
f1 (ppm)

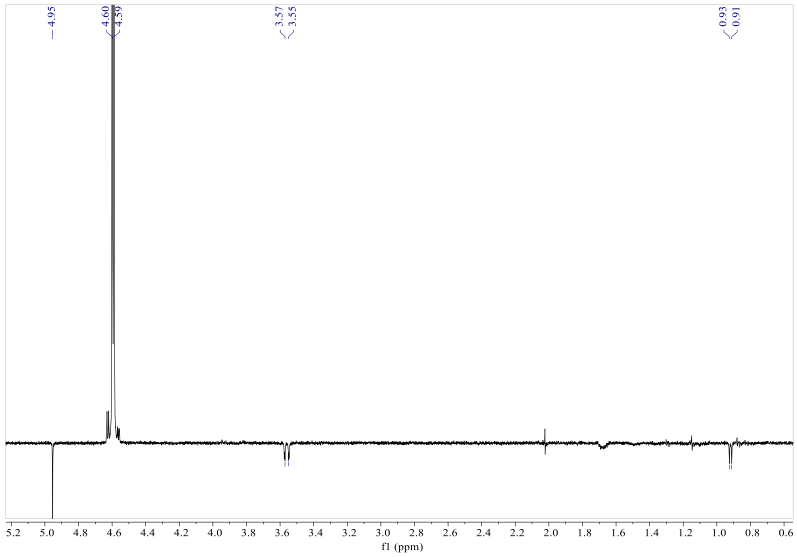
4.95
4.60
4.59
3.57
3.55
0.93
0.91
f1 (ppm)

Figure S48 1D-NOE spectrum of Lemnadiolbourside C (6) (600 MHz; $CDCl_3$)

4. NMR spectrums of the known isolated compounds

4.1 Spectroscopic data for compound 7

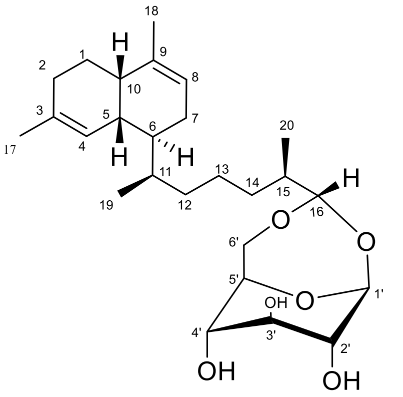

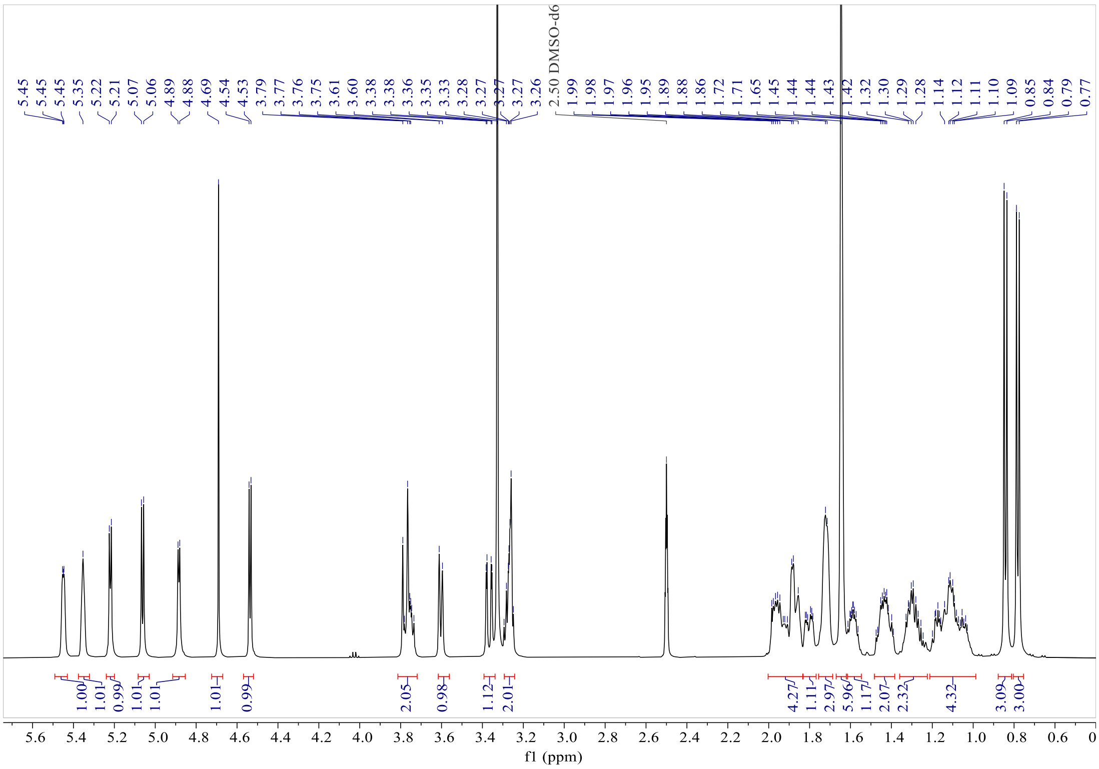

Figure S49 ^{1}H NMR Spectrum of compound 7 (600 MHz; DMSO-d_6)

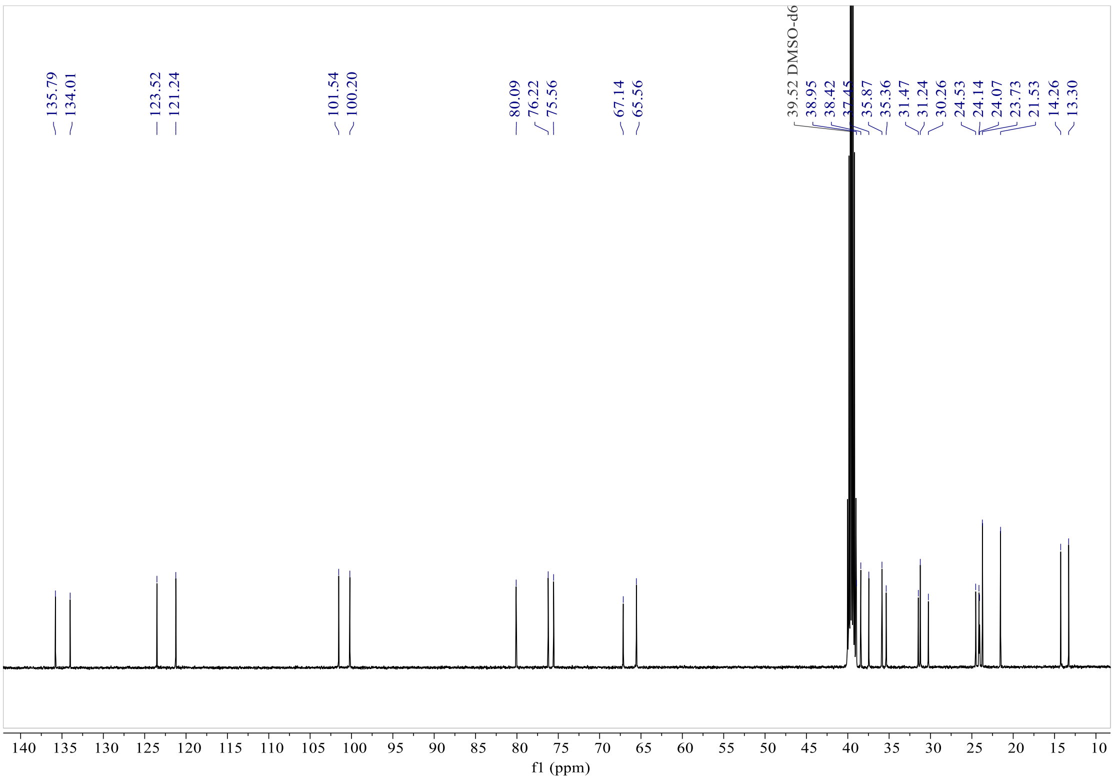

Figure S50 ^{13}C NMR Spectrum of compound 7 (150 MHz; DMSO-d$_6$)

4.2 Spectroscopic data for compound 8

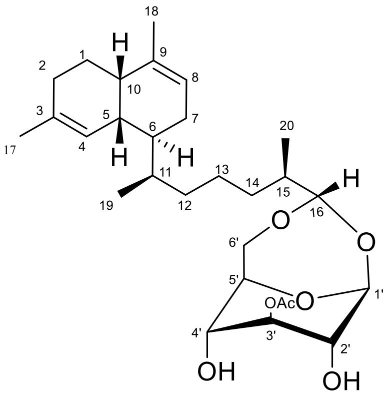

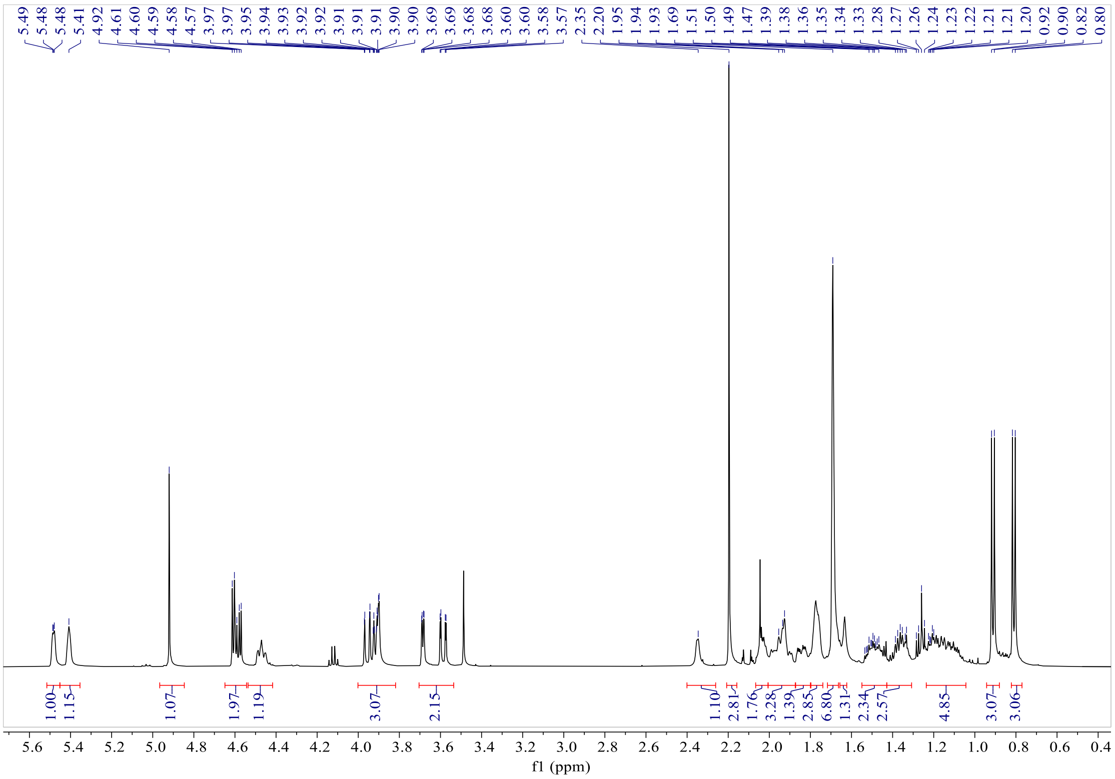

Figure S51 ^{1}H NMR Spectrum of compound 8 (600 MHz; $CDCl_3$)

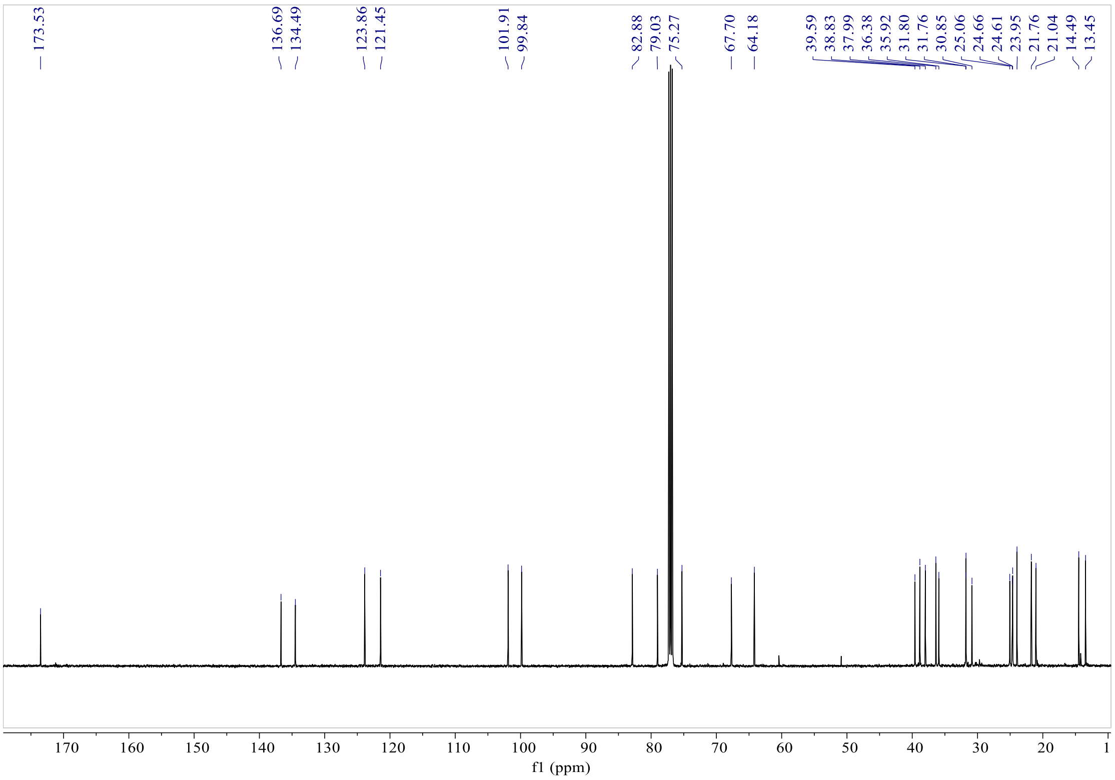

Figure S52 ^{13}C NMR Spectrum of compound 8 (150 MHz; $CDCl_3$)

4.3 Spectroscopic data for compound 9

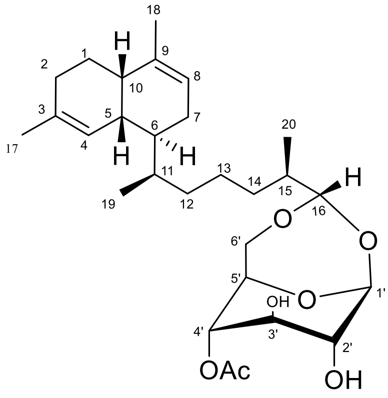

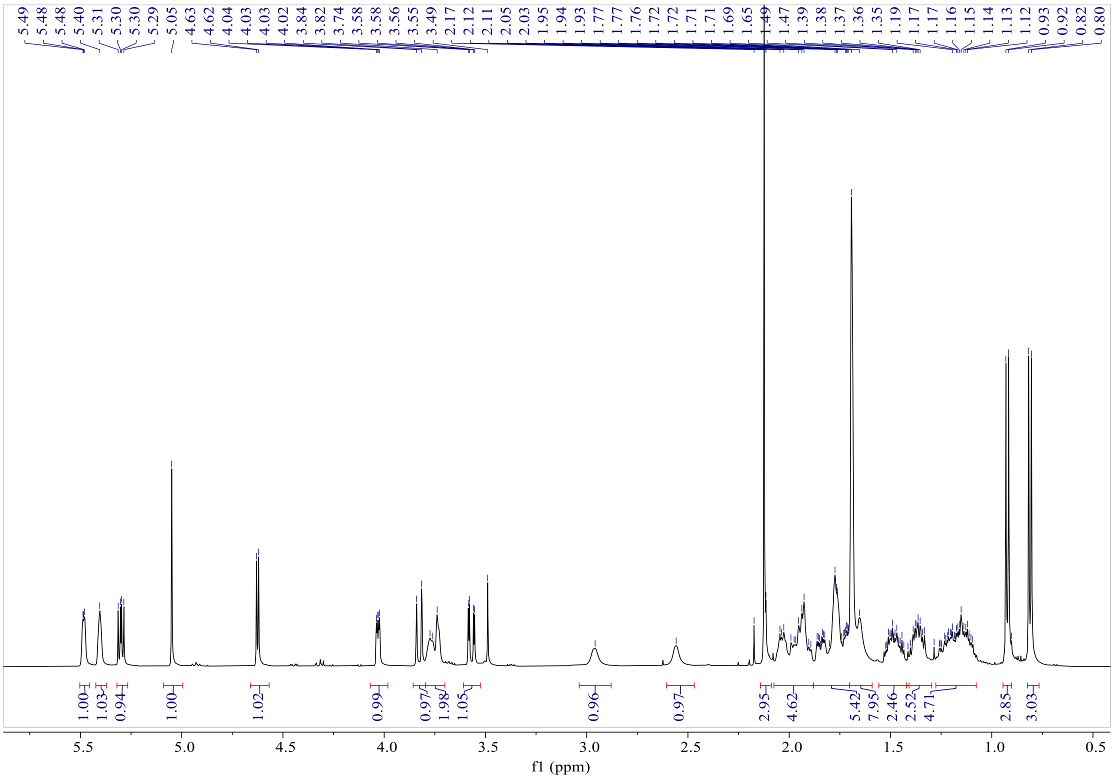

Figure S53 ¹H NMR Spectrum of compound 9 (600 MHz; CDCl₃)

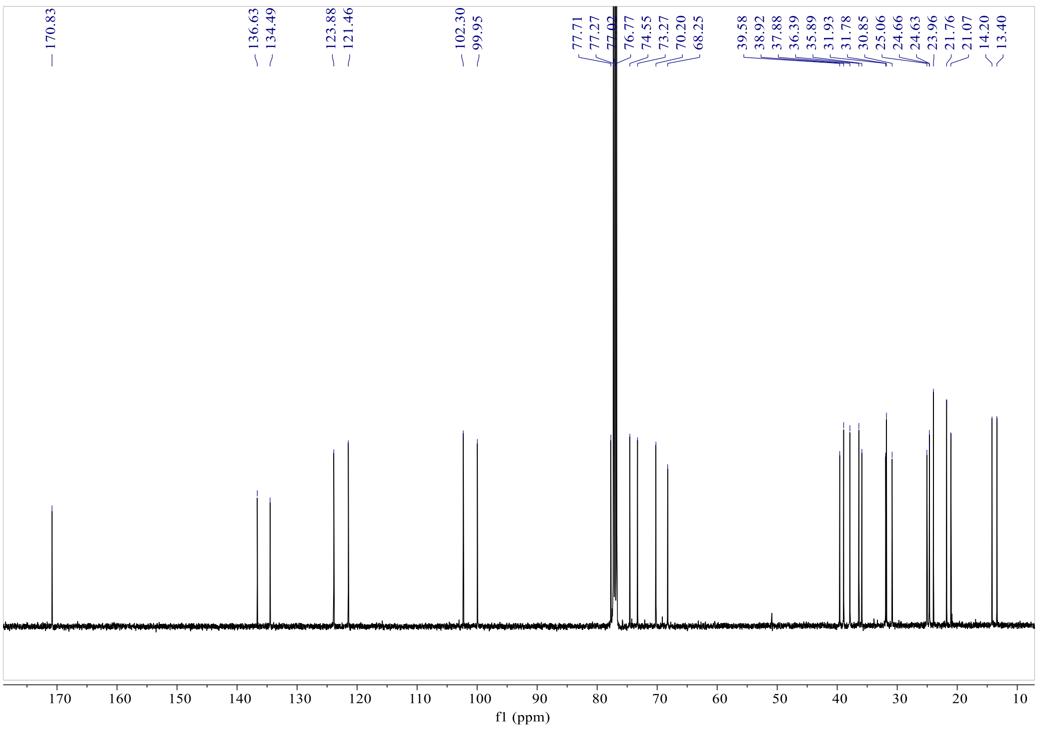

Figure S54 ^{13}C NMR Spectrum of compound 9 (150 MHz; $CDCl_3$)

2. ^{1}H NMR data for bicyclic diterpene aldehyde aglycon and bicyclic diterpene alcohol aglycon

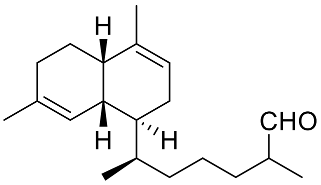

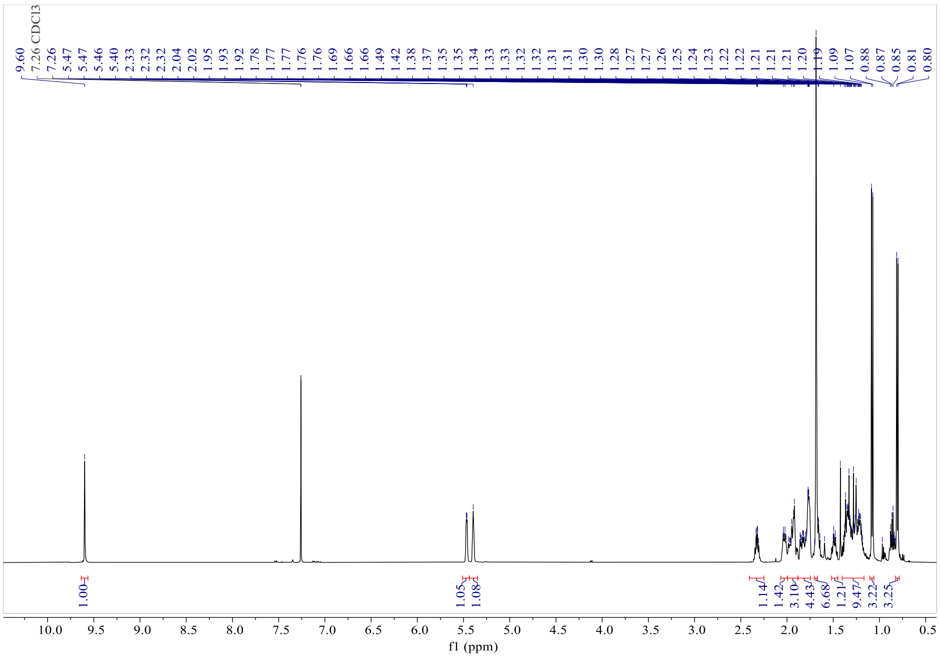

Figure S55 ^{1}H NMR Spectrum of bicyclic diterpene aldehyde aglycon (600 MHz; $CDCl_3$)

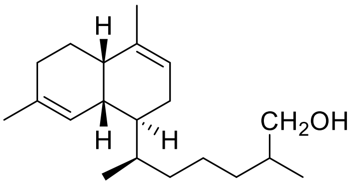

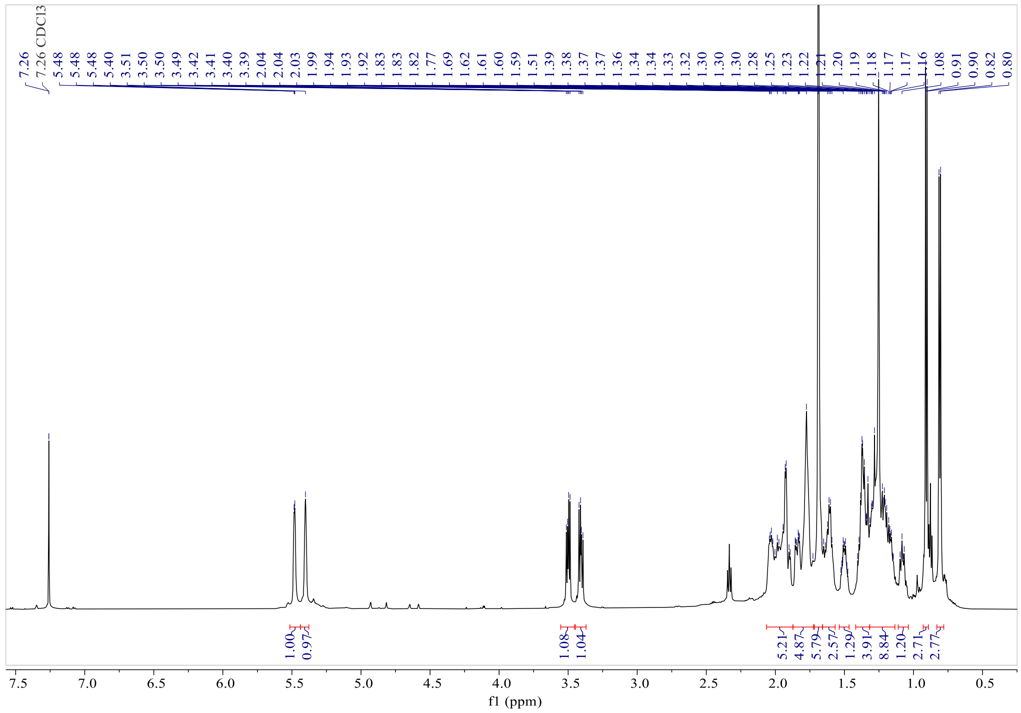

Figure S56 ^{1}H NMR Spectrum of aglycon of bicyclic diterpene alcohol aglycon (600 MHz; $CDCl_3$)

6. HPLC chromatograms of the sugar derivatives of compounds 1-6 and the standard D-glucose

D-GLU

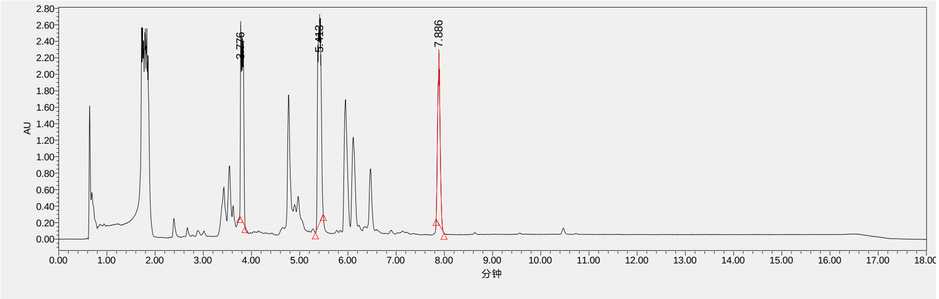

Compound 1

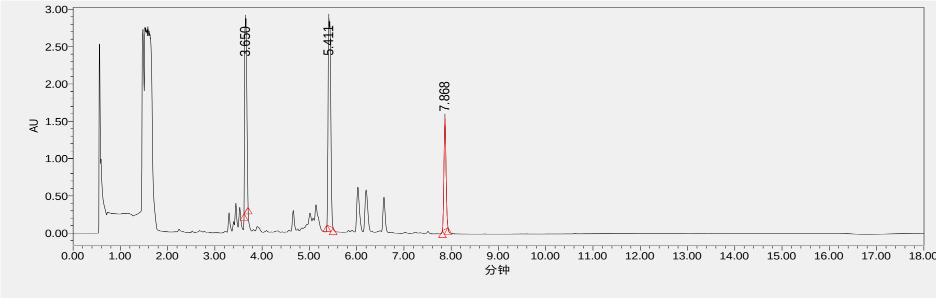

Compound 2

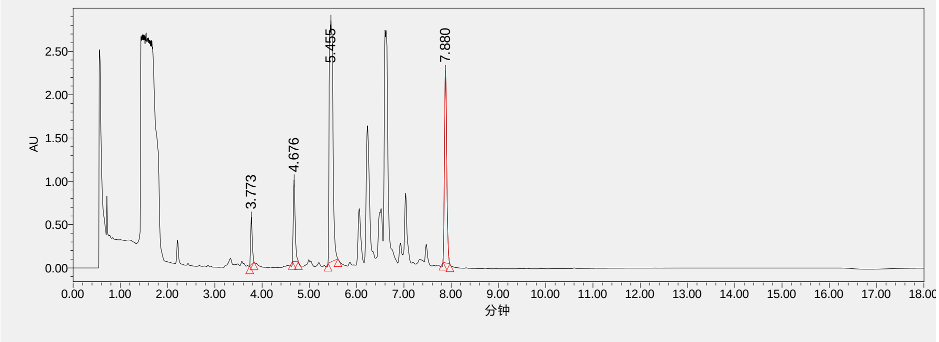

Compound 3

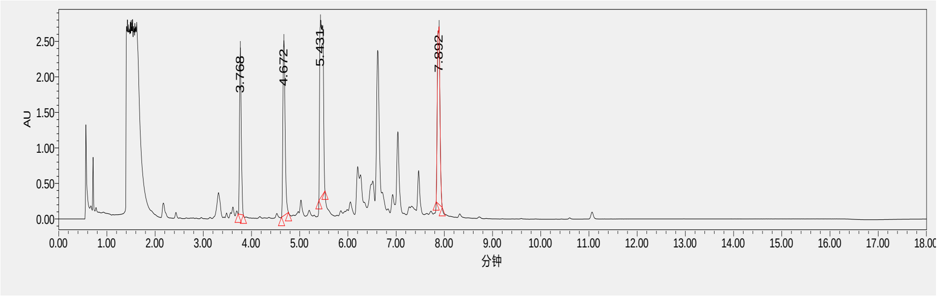

Compound 4

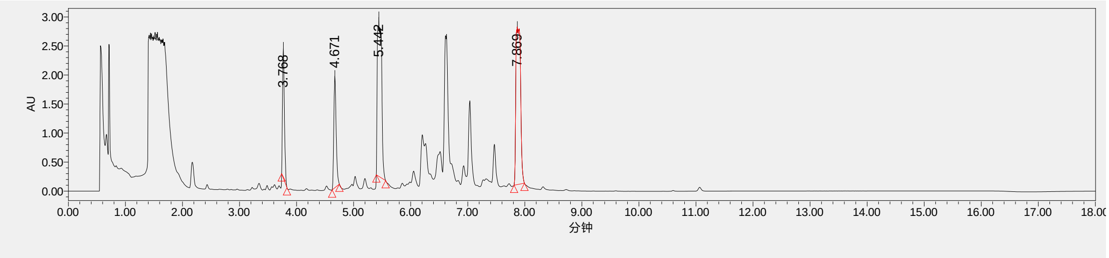

Compound 5

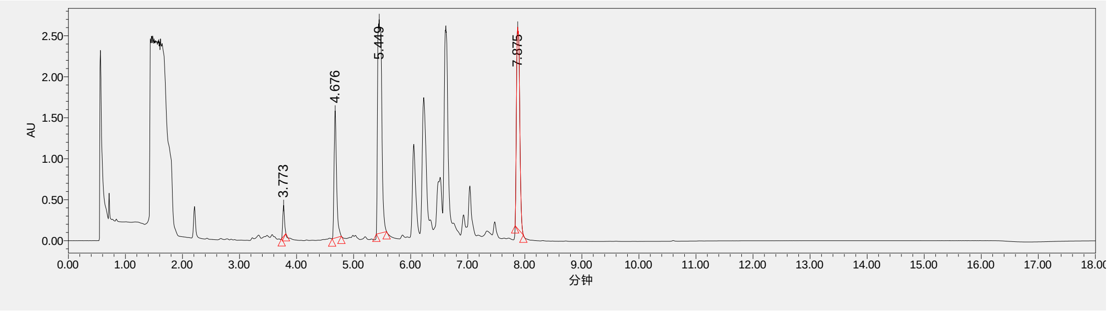

Compound 6

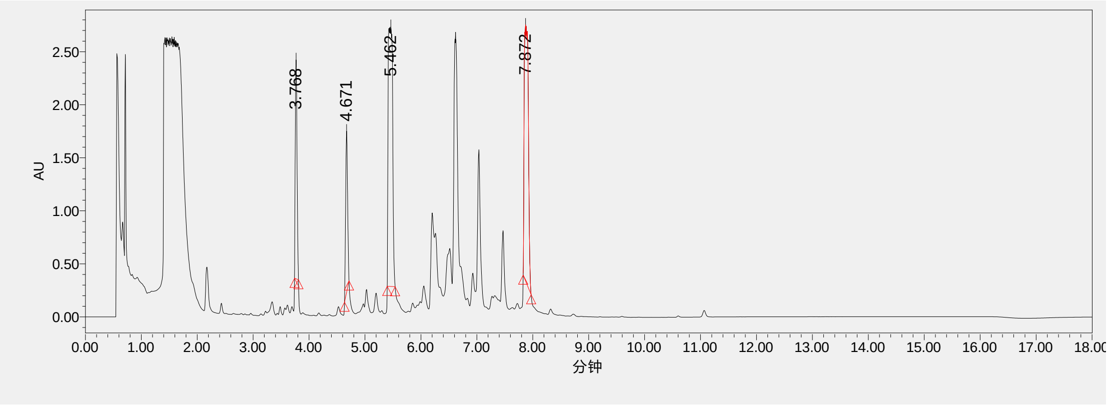

Figure S57 HPLC chromatograms of the sugar derivatives of compounds 1-6 and the standard D-glucose

7. Photos of *Lemnalia bournei* underwater and fresh sample after collected

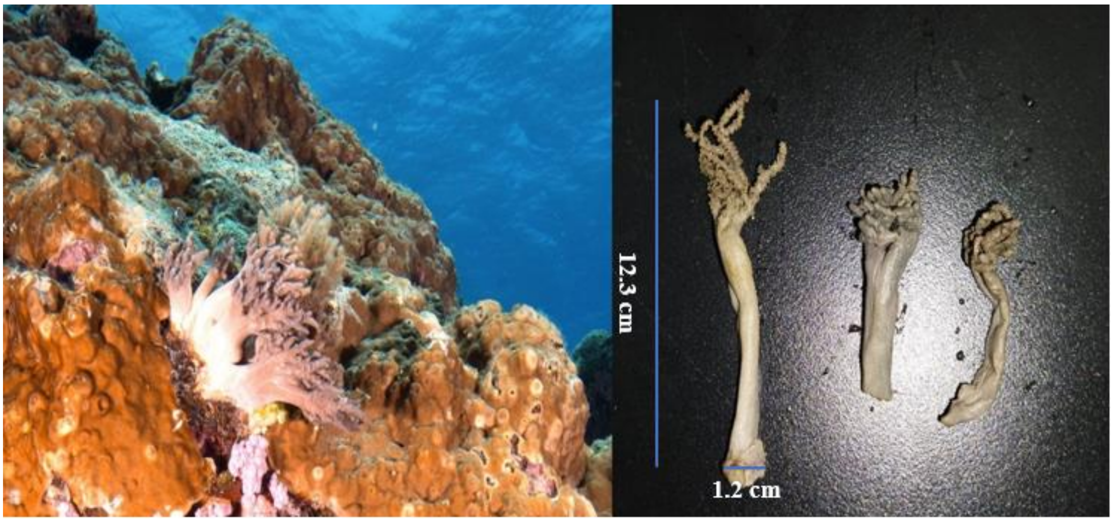

Figure S58 Photos of *Lemnalia bournei* underwater and fresh sample after collected

8. Photomicrographs of the spicules extracted from *Lemnalia bournei*

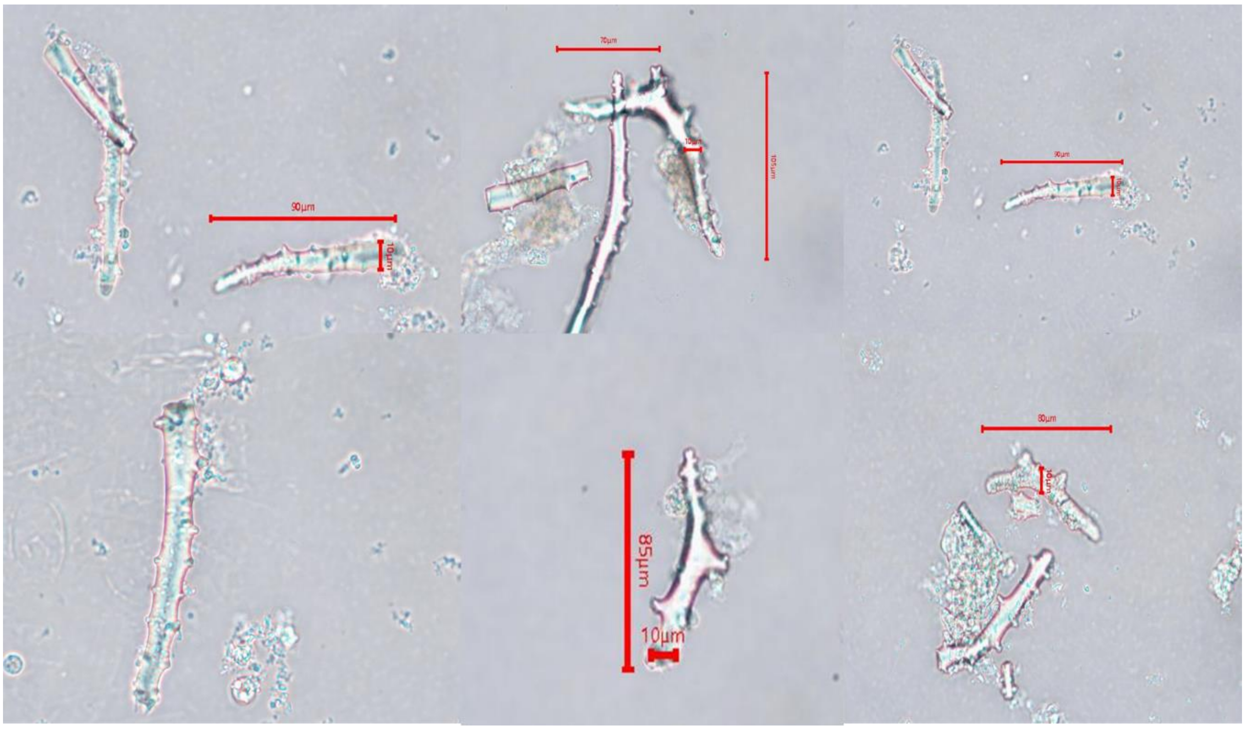

Figure S59 Photomicrographs of the spicules extracted from the soft coral. a) spicules from the tentacles; b) spicules from the cortex of the distal part of the stem; c) spicules from the cortex of the distal part of the stem
